# Individualized Exercise Training at Maximal Fat Oxidation Combined with Fruit and Vegetable-Rich Diet in Overweight or Obese Women: The LIPOXmax-Réunion Randomized Controlled Trial

**DOI:** 10.1371/journal.pone.0139246

**Published:** 2015-11-10

**Authors:** Florent Besnier, Victorine Lenclume, Patrick Gérardin, Adrian Fianu, Jérémy Martinez, Nadège Naty, Sylvaine Porcherat, Karim Boussaid, Stéphane Schneebeli, Eric Jarlet, Sarah Hatia, Georges Dalleau, Chantal Verkindt, Jean-Frédéric Brun, Marie-Paule Gonthier, François Favier

**Affiliations:** 1 INSERM, CIC 1410, Saint-Pierre, F-97410, France; 2 Unit of Diabetology, Endocrinology, Metabolic Diseases, University Hospital of La Réunion, Saint Pierre, La Réunion; 3 UMR DETROI, INSERM U1188, University of La Reunion, Sainte Clotilde, La Réunion; 4 Intercultural Determinants of Motricity and Sports Performance Research Group (DIMPS), University of La Réunion, Le Tampon, La Réunion; 5 Department of Clinical Physiology (CERAMM, U1046 INSERM), University Hospital of Montpellier, France, UMR9214 CNRS, « physiology and experimental medicine: heart–muscles », University of Montpellier, Montpellier, France; University of Tolima, COLOMBIA

## Abstract

**Objectives:**

Lifestyle combined interventions are a key strategy for preventing type-2 diabetes (T2DM) in overweight or obese subjects. In this framework, LIPOXmax individualized training, based on maximal fat oxidation [MFO], may be a promising intervention to promote fat mass (FM) reduction and prevent T2DM. Our primary objective was to compare three training programs of physical activity combined with a fruit- and vegetable-rich diet in reducing FM in overweight or obese women.

**Design and setting:**

A five months non-blinded randomized controlled trial (RCT) with three parallel groups in La Réunion Island, a region where metabolic diseases are highly prevalent.

**Subjects:**

One hundred and thirty-six non-diabetic obese (body mass index [BMI]: 27–40 kg/m^2^) young women (aged 20–40) were randomized (G1: MFO intensity; G2: 60% of VO_2_-peak intensity; G3: free moderate-intensity at-home exercise following good physical practices).

**Outcomes:**

Anthropometry (BMI, bodyweight, FM, fat-free mass), glucose (fasting plasma glucose, insulin, HOMA-IR) and lipid (cholesterol and triglycerides) profiles, and MFO values were measured at month-0, month-3 and month-5.

**Results:**

At month-5, among 109 women assessed on body composition, the three groups exhibited a significant FM reduction over time (G1: -4.1±0.54 kg; G2: -4.7±0.53 kg; G3: -3.5±0.78 kg, *p*<0.001, respectively) without inter-group differences (*p* = 0.135). All groups exhibited significant reductions in insulin levels or HOMA-IR index, and higher MFO values over time (*p*<0.001, respectively) but glucose control improvement was higher in G1 than in G3 while MFO values were higher in G1 than in G2 and G3. Changes in other outcome measures and inter-group differences were not significant.

**Conclusion:**

In our RCT the LIPOXmax intervention did not show a superiority in reducing FM in overweight or obese women but is associated with higher MFO and better glucose control improvements. Other studies are required before proposing LIPOXmax training for the prevention of T2DM in overweight or obese women.

**Trial Registration:**

ClincialTrials.gov NCT01464073

## Introduction

Over the past three decades, the burden of obesity has nearly doubled worldwide [1]. Indeed, according to a recent World Health Organization (WHO) report, 1.46 billion adults were overweight (body mass index [BMI]≥ 25 kg/m²) in 2008, and of these, 205 million men and 297 million women were obese (BMI≥ 30 kg/m²). In La Réunion Island, Favier and co-workers confirmed in the REDIA study that women have a higher prevalence of overweight or obesity than men (47% *vs* 43%, BMI > 25 kg/m² *p* = 0.002) [2].

The causal relationship between obesity, metabolic syndrome (MS) and conditions such as type-2 diabetes mellitus (T2DM), cardiovascular diseases (CVD) and cancer is now well documented. [3] Thus, in our setting, sedentary lifestyle and the high prevalence of obesity may partially explain the high prevalence of T2DM in the 30–69 age group for women (17.3%) [2,4]. In line with this picture, the literature dedicated to preventive strategies for obesity and related complications provides evidence that lifestyle interventions combining regular physical activity (PA) and diet were cost-effective [5–8]. As visceral adiposity is the milestone of cardiovascular complications, it is thus imperative to propose preventive measures based on fat mass (FM) reduction in obese people. However, despite the high level of proofs and indisputable usefulness, the level of prescription of strategies based on regular PA remains poor in this population. According to the American College of Sports Medicine, a dose–response is expected between the amount of PA and the intensity of weight reduction: “less than 150 minutes a week of PA promotes minimal weight loss, PA > 150 min/wk results in moderate weight loss of ~2–3 kg, PA between 225–420 min/week results in 5–7.5 kg weight loss”. Importantly, “PA improves weight loss when diet restriction is modest but not when diet restriction is substantial” [9]. In this framework, a growing body of evidence has shown that the benefits of PA in obese people are limited when it is not individualized, regular, and based on a “moderate intensity”. The concept of individualization of exercise training has led some experts to recommend the use of sub-maximal self-parameters for guiding the prescription of PA exercises [10,11]. Moreover, Salvadego and coll [12]. have proposed that PA prescription in obese people takes into account the "metabolic answer" to the effort, represented by the adaptation kinetics of several parameters including oxidative metabolism and exercise tolerance biomarkers, and not only the maximal oxygen consumption (VO2 peak) or the maximal heart rate (HR peak).

In this context, the threshold of maximal lipid oxidation has been sought [13]. This is usually measured by indirect calorimetry during an incremental exercise test. According to the balance of carbohydrate and lipid utilization during exercise [14], carbohydrates oxidation (CHO) increases proportionally to the intensity of exercise, whereas lipids oxidation reaches a maximum at an intensity defining the “LIPOXmax” and then decreases. LIPOXmax differs between individuals, and its measure allows the determination, for each subject, of the optimal intensity in an effort to achieve maximal lipid consumption during exercise sessions. So far, the use of indirect calorimetry enables the prescription of an individualized training program in order to optimize the oxidation of lipids. Several works have highlighted the efficiency of LIPOXmax training on FM loss, glucose control and muscular metabolism in obese or diabetic subjects [15–27]. However, in these studies the lack of control group did not allow to conclude whether the improvements are due to the LIPOXmax training or to the endurance exercise training *per se*. To the best of our knowledge, no study has yet compared the individualized LIPOXmax training to a standardized training guided by the percentage of VO2 peak, or by the Guideline of Good Physical Practices (GPP) in obese women.

Alongside individualized PA training programs, the WHO encourages the consumption of at least five portions of fruits and vegetables per day [28]. Micronutrients, fibre, and phytochemicals are associated with risk reductions of CVD [29] and T2DM [30]. Furthermore, FM loss is facilitated when a fruits and vegetables diet is combined with regular PA [9]. Unfortunately, obese subjects consume fewer vitamins and antioxidants of fruits and vegetables origin compared with non-obese subjects [31–33]. Thus, fruits and vegetables consumption must be strongly encouraged in this at-risk population.

The primary objective of the “LIPOXmax-Réunion” study was to compare three PA training programs combined with fruits and vegetables supplementation, in reducing FM in overweight or obese sedentary women. Secondary objectives were to assess changes in body composition, glucose and lipid profiles, and maximal fat oxidation measures.

## Methods

The protocol of the LIPOXmax-Réunion RCT and supporting CONSORT checklist are available as supporting information; see S1 Protocol (full protocol in French), S2 Protocol (brief English translation), and S1 CONSORT Checklist.

### Design and setting

The “LIPOXmax-Réunion” study was a non-blinded, randomized controlled trial (RCT) with three parallel groups. All participants were recruited in a single centre (CHU Sud-Réunion) between November 2011 and April 2012 and followed-up over five months, between December 2011 and September 2012.

### Participants

After providing written consent for participation, eligible overweight or obese women were enrolled in the study. The eligible criteria were, as follows: aged between 20 and 40, overweight or obese (BMI: 27 to 40 kg/m²), sedentary life style (< 2 hours of PA per week), clinically stable, namely non-diabetic (fasting plasma glucose < 7 mmol/L, HbA1c < 6.5%), and able to practise exercise training.

Non-inclusion criteria were hypertension (≥140/90 mmHg), contra-indication to PA training, cardiovascular and/or respiratory disease diagnosed by a cardiorespiratory exercise test, myopathy, cancer, acute and chronic inflammatory diseases, end-stage renal disease, digestive system surgery, previous treatment by steroids, thyroid hormone HRT, antidepressants or neuroleptics, pregnancy, participation in the previous month in a program of training or a diet.

### Ethics and funding

This research was approved by the Ethical Research Committee of the University of Bordeaux III and was registered with an approved ICMJE clinical trial registry: NCT01464073 ClinicalTrials.gov. https://clinicaltrials.gov/ct2/show/NCT01464073. This research was supported by grants of La Réunion island authorities (CPER 2012) and by the European Research and Development Fund (POCT FEDER 2007–2013).The sponsors played no role in determining the study design, data collection, analysis and interpretation, or writing of the report. The corresponding author had full access to all the data and had the final responsibility for the decision to submit for publication.

### Randomization

After inclusion, subjects were randomly assigned to one of the three groups: G1 (LIPOXmax); G2 (60% VO2max); G3 (GPP at-home, 30 minutes minimum per day at a moderate intensity).

Randomization was stratified on baseline BMI status defined as follows: stratum 1: 27.0 ≤ BMI < 30.0 kg/m², stratum 2: BMI ≥ 30.0 kg/m². The two lists of randomization were provided by the statistician of the Centre for Clinical Investigation (AF) before starting the research, using Ralloc program of Stata v10.0® (randomization by block with equal block size = 3, and balanced allocation ratio 1:1:1). The statistician gave the individual intervention’s assignment to the research coordinator who informed the participant.

### Intervention

#### Exercise training programs

All training sessions for G1 and G2 were performed in the hospital on cyclo-ergometers (CARE–Sprinter XP) and were supervised by a PA coach (JM). G1 (LIPOXmax) was conducted at a low-endurance intensity eliciting the maximal lipid oxidation measured by indirect calorimetry, four times a week for 55 minutes. G2 (60% VO2max) subjects exercised four times a week for 35 minutes at moderate-intensity, corresponding to 60% of the VO2 peak. The duration of PA was levelled between groups to an isocaloric exercise per session of 20 kJ.kg Fat Free Mass^-1^. G3 (GPP at-home) subjects were assigned a daily free practice of 30 minutes of moderate-intensity exercise. All the participants were given a pedometer (Dista Newfeel 400®), a HR monitor (Kalenji 300®) and a PA booklet where the duration (minutes), intensity (recorded on a Borg scale graded 1 to 10), and the type of exercise had to be noted. This booklet was checked twice a month.

#### Dietary intervention

To ensure a healthy balanced diet throughout the RCT, all subjects participated in a nutritional educational workshop conducted by a dietician prior to the start of the PA programs. Over the 5-month intervention period, all subjects received a free supplementation of five portions of fruits and vegetables a day.

#### Outcome measures

The primary outcome was the FM change expressed in kilograms and measured by Dual X-ray absorptiometry (DXA–GE Healthcare, Little Chalfont, United Kingdom) between the inclusion (M0) and the end of the intervention at the fifth month (M5). Secondary outcomes were measured at inclusion (M0), at the third- (M3) and at the fifth-month (M5). They included total body mass, FM, fat free mass (FFM), plasma lipids (total cholesterol, HDL-cholesterol, LDL-cholesterol, triglycerides), fasting plasma glucose (FPG), glycosylated haemoglobin (HbA_1_c), insulin resistance (as measured by the HOMA-IR index), and the exercise’s metabolic profile with the determination of the maximal fat oxidation (MFO).

#### Anthropometric measures

After a whole fasting night, total body mass and regional body composition were measured by DXA. Height was measured to the nearest 0.5 cm using a standardized height gauge. BMI was calculated as weight (kg)/height² (m).

#### Maximal cardiorespiratory exercise test

Cardiorespiratory fitness was measured during a cyclo-ergometer incremental test (Ergoline Bosch 500, Berlin, Germany). Oxygen (VO_2_) and carbon dioxide (CO_2_) were registered by a breath by breath analysis and heart rate (HR) was recorded continuously by a 12-lead electrocardiogram (Ergostik, Geratherm Medical AG, Geschwenda, Germany). After a warm upload of 20 Watts for two minutes, 15 Watts increments were applied each minute, up to exhaustion. Maximal oxygen consumption (VO2peak) was calculated in the last 30 seconds of the test. Maximal aerobic power output (MAP in W) was determined as the power developed at the latest accomplished workload.

#### Metabolic exercise test

After a whole fasting night, the women performed an exercise test on a cyclo-ergometer connected to the analyser for gas exchange measurements (VO_2_ and VCO_2_) and to the HR monitor. The test consisted of a progressive five- to six-minute steady state workload corresponding to 20%, 30%, 40%, 50%, 60% of MAP as generally used to individualize the increment of testing [13,17]. VO_2_, VCO_2_ and HR were recorded during the last two minutes of each stage in order to calculate the substrate oxidation flow rates [34].

#### Maximal Fat Oxidation

Substrate oxidation was calculated from the measurement of the respiratory exchange ratio (RER = VCO_2_/VO_2_) to determine whole body substrate oxidation. RER is the most widely used method for determination of fuel utilization. Fat oxidation rates were calculated from the gas exchange measurements according to the non-protein respiratory quotient technique using the following equation: Fat (mg.min^-1^) = -1.7012×VCO_2_ + 1.6946×VO_2_ (gas volume expressed in ml.min^-1^) [35].

The MFO is the point where fat oxidation induced by increasing workload reaches a maximum, followed by a decrease when CHO becomes the predominant fuel. The corresponding HR expressed as beats per minute (bpm) at MFO intensity was recorded individually and then applied to control the intensity of exercise training in G1 (LIPOXmax).

### Statistical analysis

#### Prior sample size calculation

The primary outcome measure was the individual FM change from the inclusion to the end of the follow-up, five months later (Δ_M5-M0_, in kg). To the best of our knowledge, LIPOXmax intervention, sustained over five months, had never been compared to other PA training programs in a population of young overweight or obese women; thus it was not possible to use an expected effect value in sample size calculation. As a consequence, our RCT was designed to detect a minimal FM difference of 1.5 kg (standard deviation: ± 2.0 kg) between G1 and G2. For multiple comparison purposes, α risk was set to 1.67% using Bonferroni correction (0.05:3), statistical power (1-β) to 80%, in bilateral hypothesis.

Under these conditions, 38 women per group had to be recruited and followed to satisfy the analysis.

This number was raised to 42 to anticipate a 10% loss due to protocol deviation or loss-to-follow-up, leading to the enrolment of a total of 126 women.

#### Statistical methods

Data were summarized by mean ± standard deviation (SD) or 95% confidence interval (95% CI), median (interquartile range) and percentages. Baseline inclusion characteristics were compared between groups using one-way ANOVA or Kruskal-Wallis non parametric test for quantitative variables, or using Chi2 test or Fisher exact test for qualitative variables, as appropriate. Baseline exercise tests at inclusion were compared between groups using one-way ANOVA or Kruskal-Wallis non parametric test, as appropriate. Changes in anthropometric characteristics were compared between groups using one-way ANOVA or Kruskal-Wallis non-parametric test, as appropriate, post-hoc analysis being performed with Student-T tests. Repeated ANOVA measures allowed estimating time effect (M0-M3-M5), group effect (G1-G2-G3) and the interaction time*group. Wilcoxon rank-sum test was used to analyse the differences between G1 and G2.

The primary outcome and the other five-month changes were analyzed between M5 and M0 within the group of subjects for whom these data were available. The longitudinal evolution of metabolic parameters was analyzed for the subjects for whom the M0, M3 and M5 data were complete. Missing observations were excluded. Except for the aforementioned post hoc analysis, the significance level was set to 5%. Analyses were carried out using SAS® version 9.2 (SAS Institute Inc., Cary, NC, USA).

## Results

### Selection of participants

**Fig 1:**
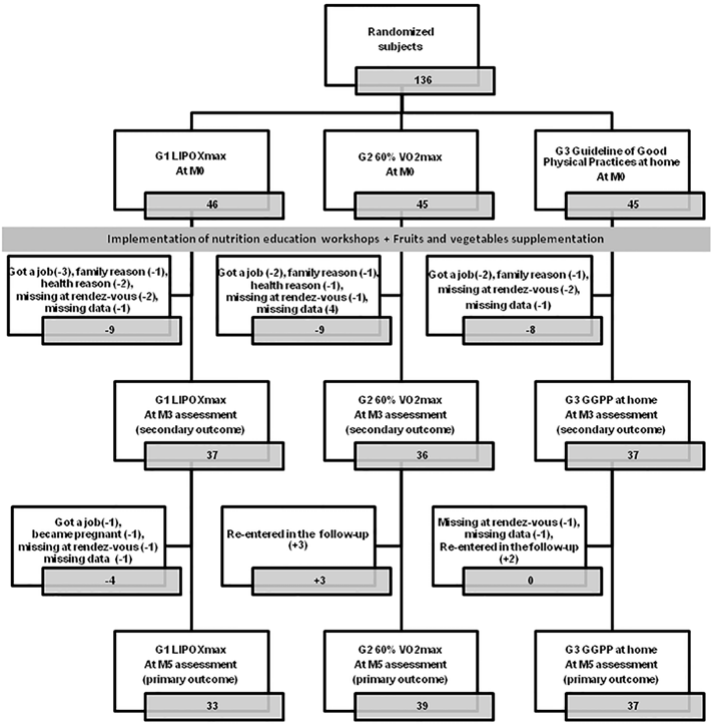
LIPOXmax Réunion Flow chart.

The LIPOXmax–Réunion flow chart depicting the distribution of the participants throughout the RCT is presented in Fig 1. Of the 156 women eligible for the study, 136 were enrolled and randomized between November 2011 and April 2012. Of these, 109 subjects were subsequently followed-up five months between December 2011 and September 2012. They were assessed at M0 and M5 (80.1% of all randomised subjects), and among them, 103 had satisfied the complete protocol with M0, M3 and M5 evaluations (75.7% of all randomised subjects). No selection bias was observed between randomized participants and non-participants, regardless of the definition of participation used (subjects assessed at M0-M5, or at M0-M3-M5).

### Baseline characteristics

The physical characteristics of subjects are presented in Table 1. Women were aged on average 30 years old. More than three-quarters of them were obese (BMI ≥ 30 kg/m²) and 86.6% had a high waist circumference according to the NCEP/ATP-III thresholds.

Overall, the percentage of FM was 47.1% and the one of FFM 51.3%. Eighty percent were insulin resistant (HOMA-IR index > 2.5). Mean of MFO was of 153.4 mg/min. At M0, there was no difference among the three groups in socio-demographic and anthropometric data (Tables 1 and 2). There were no differences between subjects who dropped out and subjects who participated in all of the longitudinal study, which could distort the basic characteristics (Fig 1). Analyses of bias of selection are available as supporting information; see S1–S4 Tables.

**Table 1:** Baseline inclusion characteristics for LIPOXmax-Réunion randomized controlled trial participants.

		All groups	G1: LIPOXmax	G2: 60% VO2max	G3: GPP at home	p-value
		N = 136	N = 46	n = 45	n = 45	
**Socio-demographic characteristics**						
Age (years)		30.1 ± 5.6	30.5 ± 5.9	29.0 ± 4.9	30.9 ± 5.8	0.186*
School level:						
Secondary school (pupils from 10 to 15)		8.8%	15.2%	8.9%	2.2%	0.134*
Upper forms (pupils from 15 to 18)		46.3%	47.8%	37.8%	53.3%	
A-level or Higher school		44.9%	37.0%	53.3%	44.4%	
Number of children at home ^†^		1.0 (1.0–2.0)	2.0 (1.0–2.0)	1.0 (1.0–2.0)	2.0 (1.0–2.0)	0.167*
Number of children ^‡^		2.0 (1.0–2.0)	2.0 (1.0–3.0)	1.0 (1.0–2.0)	2.0 (1.0–3.0)	0.181*
Recipient of CMU		64.0%	69.6%	62.2%	60.0%	0.609
Living in a couple		51.5%	54.3%	42.2%	57.8%	0.300
**Anthropometric characteristics**						
Height (cm)		161.5 ± 5.8	162.6 ± 4.6	160.6 ± 6.6	161.4 ± 6.1	0.263
Weight (kg)		86.6 ± 11.6	88.2 ± 11.5	85.5 ± 12.1	86.1 ± 11.5	0.518
BMI (kg/m²)		33.1 ± 3.5	33.3 ± 3.8	33.1 ± 3.5	33.0 ± 3.3	0.950*
< 30 kg/m²		24.3%	21.7%	24.4%	26.7%	0.860
≥ 30 kg/m²		75.7%	78.3%	75.6%	73.3%	
Waist size (cm)		97.9 ± 9.0	98.7 ± 9.4	96.3 ± 8.5	98.8 ± 8.9	0.342
< 88 cm		12.5%	6.5%	17.8%	13.3%	0.256*
≥ 88 cm		87.5%	93.5%	82.2%	86.7%	
Hips (cm)		121.0 ± 8.3	121.0 ± 8.5	121.1 ± 9.3	121.0 ± 7.2	0.996
Report Waist / Hips		0.8 ± 0.1	0.8 ± 0.1	0.8 ± 0.1	0.8 ± 0.1	0.302
Report Waist / Hips:	< 0.9	89.0%	87.0%	88.9%	91.1%	0.941*
≥ 0.9		11.0%	13.0%	11.1%	8.9%	
Fat mass (kg)		39.9 ± 7.9	40.2 ± 7.8	40.2 ± 8.5	39.3 ± 7.4	0.819
Fat mass (%)		47.1 ± 3.8	46.6 ± 3.8	47.9 ± 4.0	46.7 ± 3.6	0.195
**Anthropometric characteristics**						
Troncal Fat Mass (kg)		18.6 ± 4.6	18.8 ± 5.0	18.6 ± 4.4	18.3 ± 4.6	0.885
Android Fat Mass (kg)		3.4 ± 1.0	3.4 ± 0.9	3.4 ± 1.0	3.4 ± 1.0	0.933
Gynoid Fat Mass (kg)		7.0 ± 1.5	7.0 ± 1.4	7.2 ± 1.8	6.9 ± 1.3	0.542
Fat Free Mass (kg)		44.2 ± 4.8	45.4 ± 5.0	42.9 ± 4.3	44.3 ± 5.0	0.059*
Fat Free Mass (%)		51.3 ± 3.6	51.8 ± 3.6	50.6 ± 3.8	51.7 ± 3.4	0.206
Troncal Fat Free Mass (kg)		18.5 ± 2.3	19.0 ± 2.4	17.9 ± 1.9	18.6 ± 2.5	0.051
Android Fat Free Mass (kg)		2.9 ± 0.4	3.0 ± 0.4	2.8 ± 0.3	3.0 ± 0.4	0.050
Gynoid Fat Free Mass (kg)		6.9 ± 0.9	7.1 ± 0.8	6.8 ± 0.9	6.9 ± 0.9	0.234
**Blood pressure**						
Systolic blood pressure (mm Hg)		117.0 ± 9.9	117.8 ± 9.2	115.7 ± 11.4	117.5 ± 9.1	0.565
Diastolic blood pressure (mm Hg)		74.4 ± 8.5	74.8 ± 8.8	74.3 ± 8.7	74.3 ± 8.1	0.950
**Lipid profile**						
Total Cholesterol (mmol/L)		4.5 ± 0.8	4.5 ± 0.8	4.5 ± 0.9	4.6 ± 0.8	0.890
LDL Cholesterol (mmol/L)		2.8 ± 0.7	2.8 ± 0.8	2.8 ± 0.7	2.8 ± 0.7	0.929
HDL Cholesterol (mmol/L)		1.2 ± 0.3	1.3 ± 0.3	1.2 ± 0.3	1.3 ± 0.3	0.773
HDL-C/LDL-C ratio		0.48 ± 0.21	0.49 ± 0.25	0.47 ± 0.17	0.49 ± 0.19	0.699*
Triglycerides (mmol/L)		1.1 ± 0.5	0.9 ± 0.3	1.1 ± 0.6	1.2 ± 0.6	0.297*
**Glucose profile**						
HbA1c (%)		5.5 ± 0.3	5.5 ± 0.4	5.5 ± 0.3	5.4 ± 0.3	0.363*
Fasting plasma glucose (mmol/L)		5.0 ± 0.5	5.0 ± 0.4	4.9 ± 0.5	4.9 ± 0.5	0.535*
Insulin (m UI/L)		20.0 ± 11.3	20.8 ± 9.6	21.5 ± 13.7	17.6 ± 9.8	0.086*
HOMA-IR index		4.5 ± 2.8	4.7 ± 2.5	4.8 ± 3.5	3.9 ± 2.2	0.141*
Insulin resistant (HOMA-IR index > 2.5)		80.0%	84.4%	80.0%	75.6%	0.574

^†^ Data are medians and interquartile range.

^‡^ Data are medians and interquartile range. Otherwise, data are means ± SD or percentages. CMU: universal health coverage.

HOMA-IR index: Homeostasis Model Assessment estimated insulin resistance index. p-values refer to comparison between the three groups by one-way ANOVA or Kruskal-Wallis non parametric tests (*) for quantitative variables, and by Chi2 test or Fisher exact tests (*) for qualitative variables.

**Table 2:** Baseline exercise tests at inclusion for LIPOXmax-Réunion randomized controlled trial participants.

	All groups	G1: LIPOXmax	G2: 60% VO2max	G3: GPP at home	p-value
	n = 136	n = 46	n = 45	n = 45	
**Maximal exercise test**					
VO2max (L/min)	1.9 ± 0.3	1.9 ± 0.3	1.8 ± 0.2	1.9 ± 0.3	0.645
VO2max (mL/min/kg FFM)	42.5 ± 6.3	42.0 ± 6.7	43.3 ± 6.2	42.3 ± 6.1	0.632*
HRmax (bpm)	173.0 ± 11.3	170.5 ± 10.1	176.1 ± 12.0	172.5 ± 11.3	0.072*
RER	1.1 ± 0.1	1.1 ± 0.1	1.1 ± 0.1	1.1 ± 0.1	0.585*
Wmax (W)	136.0 ± 19.9	137.2 ± 21.6	132.6 ± 21.1	138.4 ± 16.2	0.429*
**Metabolic exercise test**					
HR at LIPOXmax (bpm)	120.9 ± 13.6	118.5 ± 11.6	121.3 ± 13.7	123.0 ± 15.3	0.263*
W at LIPOXmax (W)	43.4 ± 12.1	41.9 ± 11.9	42.8 ± 12.0	45.6 ± 12.5	0.397*
W at LIPOXmax (% Wmax)	32.2 ± 8.3	30.9 ± 8.7	32.5 ± 8.4	33.1 ± 7.6	0.402*
Borg at LIPOXmax (6–20)	11.2 ± 1.1	11.0 ± 1.2	11.2 ± 1.0	11.4 ± 1.1	0.089*
VO2 at LIPOXmax (ml/min)	843.5 ± 142.3	844.2 ± 113.5	825.9 ± 154.6	860.3 ± 156.5	0.522
VO2 at LIPOXmax (% VO2max)	45.7 ± 8.3	45.2 ± 7.4	45.3 ± 9.6	46.8 ± 8.0	0.621
**Maximal Lipid Oxidation**					
MFO (mg/min)	153.4 ± 42.9	151.6 ± 36.7	143.9 ± 38.4	164.6 ± 50.6	0.135*
MFO (mg/min/kg FFM)	3.5 ± 1.0	3.4 ± 0.9	3.4 ± 1.0	3.7 ± 1.0	0.170*

Data are means ± SD. FFM: Fat Free Mass. HR: Heart Rate. RER: Respiratory Exchange Ratio. W: Power. MFO: Maximal Fat Oxidation. p-values refer to comparisons between the three groups by one-way ANOVA or Kruskal-Wallis non parametric tests (*).

### Implementation of training programs

For G1 and G2, PA was levelled on the maximal and metabolic cardiorespiratory values. To obtain the same energy expenditure between each group (20 kJ.kg FFM^-1^), G1 training sessions were longer in duration than those for G2 (53 min *vs*. 37 min, p<0.001). The intensity of exercise was lower for G1 than for G2 (45% *vs*. 60% VO2max; 119 *vs*. 140 bpm; 42 *vs*. 76 Watts; p<0.001, respectively).

G3 women practised on average two different types of exercise. The examination of the PA booklets revealed that 80.6% of the women practised walking, 45.2% fitness or gymnastics, 48.4% outdoor biking, 29.0% dancing (Zumba), 19.4% swimming and 12.9% indoor biking. The weekly average number of PA sessions was 3.4 (±0.9 SD, min-max: 0.8–4.7). The weekly average time spent on physical exercises was 197.7 minutes (± 67.5 min. SD, min-max: 48.6–350.2 min.). Self-perceived intensity reported using the Borg scale ranged from three to five.

### Changes in anthropometric and body composition parameters

Anthropometric and body composition parameters were measured by DXA at M0 and M5. Accordingly, the evolution of DXA measures were analysed for the 109 subjects who had fully completed M5 data. Results are summarized in Table 3.

**Table 3:** Five-month changes in anthropometric and body composition in LIPOXmax-Réunion randomized controlled trial participants.

	G1: LIPOXmax	G2: 60% VO2max	G3: GPP at home	p-value	G1 *vs* G2	G1 *vs* G3	G2 *vs* G3
	n = 33	n = 39	n = 37				
**DXA characteristics**							
Weight (kg)	-5.0 (-6.5 to -3.4)	-5.4 (-6.8 to -4.0)	-3.5 (-5.0 to -2.1)	0.172	-	-	-
BMI (kg/m²)	-1.8 (-2.4 to -1.2)	-2.1 (-2.7 to -1.6)	-1.4 (-2.0 to -0.9)	0.194	-	-	-
Fat Free Mass (kg)	-0.8 (-1.2 to -0.3)	-0.7 (-1.1 to -0.3)	0.0 (-0.4 to 0.4)	**0.026**	0.820	**0.010**	0.032
Fat Free Mass (%)	2.2 (1.3 to 3.1)	2.7 (1.9 to 3.6)	2.6 (1.7 to 3.4)	0.703	-	-	-
Fat Mass (kg)	-4.1 (-5.4 to -2.7)	-4.7 (-5.9 to -3.5)	-3.5 (-4.8 to -2.3)	0.135*	-	-	-
Fat Mass (%)	-2.3 (-3.3 to -1.4)	-2.9 (-3.8 to -2.0)	-2.7 (-3.6 to -1.8)	0.663	-	-	-
Truncal Fat Mass (kg)	-2.4 (-3.3 to -1.5)	-3.0 (-3.8 to -2.2)	-2.1 (-2.9 to -1.3)	0.245	-	-	-
Android Fat Mass (kg)	-0.5 (-0.6 to -0.3)	-0.6 (-0.7 to -0.4)	-0.4 (-0.5 to -0.2)	0.169	-	-	-
Gynoid Fat Mass (kg)	-0.8 (-1.0 to -0.5)	-0.8 (-1.1 to -0.6)	-0.7 (-0.9 to -0.4)	0.441*	-	-	-
Truncal Fat Free Mass (kg)	-0.5 (-0.9 to -0.1)	-0.7 (-1.1 to -0.4)	-0.2 (-0.6 to 0.1)	0.148	-	-	-
Android Fat Free Mass (g)	-95.1 (-158.3 to -31.8)	-112.1 (-171.0 to -53.2)	-11.9 (-71.6 to 47.9)	**0.046**	0.702	0.059	0.020
Gynoid Fat Free Mass (g)	-139.5 (-268.2 to -10.8)	-157.3 (-277.2 to -37.3)	39.7 (-81.8 to 161.3)	0.100*	-	-	-

Data are changes from M0 to M5 (M5 minus M0): mean (95% confidence interval). M5: fifth month of intervention. *p values* refers to comparison between the three groups by one-way ANOVA model or Kruskal-Wallis non parametric test (*). If p<0.05, post-hoc analysis was performed with Student’s test to compare groups two-by-two at p = 0.0167 significance level according to Bonferroni’s method.

At M5, each group (G1, G2, G3) exhibited a significant decrease in total body weight over time (intra-group differences: -5.0±0.6; -5.4±0.7; -3.5±0.9 kg respectively, p<0.001), BMI (-1.8±0.2; -2.1±0.3; -1.4±0.3 kg/m², p<0.001) and FM (-4.1±0.5; -4.7±0.5; -3.5±0.8 kg, p<0.001). However, there were no significant inter-group differences with respect to these three parameters (p = 0.172; p = 0.194; p = 0.135, respectively). Of note, FM decreased significantly in the total body and main body segments in the three groups: -2.4±0.4 *vs* -3.0±0.4 *vs* -2.1±0.4 kg in the trunk, -0.5±0.1 *vs* -0.6±0.1 *vs* -0.4±0.1 kg in the android area, -0.8±0.1 *vs* -0.8±0.1 vs -0.7±0.1 kg in the gynoid area for G1, G2, G3, respectively. In contrast, FFM evolution over time was slightly different between groups (p = 0.026) and decreased for G1 and G2 (-0.8±0.2; -0.7±0.2 kg, respectively) while it remained stable for G3. Bonferroni correction revealed that change in FFM only differed between G1 and G3 (p = 0.010). As shown by the wide 95% CIs, over time the three groups exhibited a large variability in FM evolution (and on a broader spectrum, for all body characteristics) in response to the three different interventions.

### Changes in metabolic characteristics

Out of the 109 subjects assessed at M5, the analyses of metabolic characteristics were performed for the 103 subjects who had a complete longitudinal follow-up (M0, M3 and M5). These data are summarized in Table 4. Post hoc analyses have been performed to compare groups two by two (Tables 4 and 5).

**Table 4:** Longitudinal evolution of metabolic characteristics (month-0, month-3 and month-5) in LIPOXmax-Réunion randomized controlled trial participants.

	G1: LIPOXmax			G2: 60% VO2max			G3: GPP at home			Repeated-measures ANOVA		
	n = 33			n = 35			n = 35			p-values		
	M0	M3	M5	M0	M3	M5	M0	M3	M5	t	g	t x g
**Lipid profile**												
**Total Cholesterol (mmol/L)**	**4.5 ± 0.8**	**4.5 ± 0.9**	**4.4 ± 0.9**	**4.5 ± 0.8**	**4.5 ± 0.8**	**4.4 ± 0.8**	**4.5 ± 0.9**	**4.4 ± 0.8**	**4.3 ± 0.8**	**0.085**	**0.978**	**0.436**
**LDL Cholesterol (mmol/L)**	**2.8 ± 0.7**	**2.7 ± 0.7**	**2.7 ± 0.7**	**2.7 ± 0.7**	**2.8 ± 0.8**	**2.6 ± 0.7**	**2.8 ± 0.8**	**2.7 ± 0.7**	**2.5 ± 0.7**	**< 0.001**	**0.910**	**0.283**
**HDL Cholesterol (mmol/L)**	**1.2 ± 0.3**	**1.3 ± 0.3**	**1.3 ± 0.3**	**1.2 ± 0.3**	**1.3 ± 0.3**	**1.4 ± 0.3**	**1.3 ± 0.3**	**1.3 ± 0.2**	**1.4 ± 0.3**	**< 0.001**	**0.521**	**0.245**
**HDL-C/LDL-C ratio**	**0.46 ± 0.18**	**0.51 ± 0.21**	**0.52 ± 0.20**	**0.47 ± 0.18**	**0.51 ± 0.21**	**0.58 ± 0.24**	**0.50 ± 0.19**	**0.54 ± 0.25**	**0.63 ± 0.29**	**< 0.001**	**0.486**	**0.259**
**Triglycerides (mmol/L)**	**1.0 ± 0.3**	**1.0 ± 0.4**	**0.9 ± 0.4**	**1.1 ± 0.7**	**1.1 ± 0.4**	**1.0 ± 0.4**	**1.1 ± 0.6**	**0.9 ± 0.4**	**0.9 ± 0.4**	**0.150**	**0.492**	**0.492**
**Glucose profile**												
**HbA1c %**	**5.6 ± 0.4**	**5.4 ± 0.3**	**5.4 ± 0.4**	**5.6 ± 0.3**	**5.5 ± 0.3**	**5.4 ± 0.3**	**5.4 ± 0.3**	**5.3 ± 0.2**	**5.3 ± 0.2**	**< 0.001**	**0.149**	**0.161**
**Fasting plasma glucose (mmol/L)**	**5.0 ± 0.4**	**4.9 ± 0.3**	**4.9 ± 0.4**	**5.0 ± 0.5**	**5.0 ± 0.4**	**5.0 ± 0.5**	**4.9 ± 0.4**	**4.9 ± 0.4**	**4.9 ± 0.5**	**0.395**	**0.539**	**0.580**
**Insulin (m UI/L)**	**22.8 ± 9.7**	**18.2 ± 12.8**	**16.0 ± 9.0**	**20.4 ± 9.4**	**15.4 ± 6.6**	**15.0 ± 5.4**	**16.5 ± 7.0**	**15.4 ± 7.9**	**14.6 ± 6.6**	**< 0.001**	**0.186**	**0.015**
**HOMA-IR index**	**5.2 ± 2.5**	**4.0 ± 2.9**	**3.6 ± 2.3**	**4.5 ± 2.1**	**3.4 ± 1.5**	**3.4 ± 1.4**	**3.6 ± 1.6**	**3.4 ± 1.8**	**3.2 ± 1.5**	**< 0.001**	**0.164**	**0.011**
**Maximal Lipid Oxidation**												
**MFO (mg/min)**	**146.5 ± 37.1**	**212.8 ± 39.0**	**218.5 ± 49.7**	**145.1 ± 41.1**	**209.5 ± 38.7**	**192.7 ± 44.9**	**165.8 ± 52.1**	**191.3 ± 46.7**	**186.0 ± 48.4**	**< 0.001**	**0.262**	**< 0.001**
**MFO (mg/min/kg FFM) (n = 109)**	**3.3 ± 0.8**	**-**	**5.0 ± 1.2**	**3.3 ± 1.0**	**-**	**4.5 ± 1.0**	**3.8 ± 1.0**	**-**	**4.2 ± 0.9**	**< 0.001**	**0.691**	**< 0.001**

Data are means ± SD for 103 subjects were followed-up at M0, M3 and M5). For MFO (mg/min/kg FFM), data are mean ± SD for n = 109 (subjects who participated at M0 and M5), because fat free mass DXA was measured only at M0 and at M5. M3: third month of intervention. M5: fifth month of intervention.HOMA-IR index: HOmeostasis Model Assessment estimated insulin resistance index. MFO: Maximal Fat Oxidation. FFM: Fat Free Mass. Repeated-measures ANOVA p-values represent the main effects of time (t), g (group) and the interaction effect (time *group).

**Table 5:** Post-hoc analysis (Repeated–measures ANOVA P values) for longitudinal evolution of metabolic characteristics (month-0, month-3 and month-5).

	*All groups*			*G1 vs G2*			*G1 vs G3*			*G2 vs G3*		
	t	g	t x g	t	g	t x g	t	g	t x g	t	g	t x g
**Glucose profile**												
Insulin (m UI/L)	< 0.001	0.186	0.015	< 0.001	0.407	0.756	< 0.001	0.089	0.004	< 0.001	0.250	0.025
HOMA-IR index	< 0.001	0.164	0.011	< 0.001	0.405	0.563	< 0.001	0.084	0.002	< 0.001	0.199	0.042
**Maximal Lipid Oxidation**												
MFO (mg/min)	< 0.001	0.262	< 0.001	< 0.001	0.110	0.021	< 0.001	0.188	< 0.001	< 0.001	0.933	0.004
MFO (mg/min/kg FFM) (n = 109)	< 0.001	0.691	< 0.001	< 0.001	0.396	0.016	< 0.001	0.580	< 0.001	< 0.001	0.775	0.027

HOMA-IR index: HOmeostasis Model Assessment estimated insulin resistance index. MFO: Maximal Fat Oxidation. FFM: Fat Free Mass. Repeated-measures ANOVA P values represent the main effects of time (t), g (group) and the interaction effect (time *group). Post-hoc analysis (G1 vs G2, G1 vs G3, G2 vs G3) was performed to compare groups two-by-two at p = 0.0167 significance level according to Bonferroni’s method.

Total cholesterol, triglycerides and FPG levels were stable over time. HDL-C/LDL-C ratio improved in the three groups throughout the intervention with different patterns (rapid but slight and transient response in G1, increasing response with time in G2 and G3). HbA1c decreased over time without significant inter-group differences.

Glucose control improved throughout the intervention, the percentage of insulin-resistant women (HOMA-IR index > 2.5) falling from 80% to 60% between M0 and M5. Insulin level and HOMA-IR index decreased in each group (overall time-by-group interaction p = 0.014, p = 0.011, respectively). However, the reductions in insulin and HOMA-IR index were markedly larger for G1 when compared to G3 (G1*G3 time-by-group interactions, p = 0.004 for insulin and p = 0.002, for HOMA, respectively).

In line with these results, MFO expressed in mg.min^-1^increased significantly over time in each group (overall time-by-group interaction, p<0.001). As expected from the above data this change was larger for G1 than for G3 (time-by-group interaction G1*G3, p<0.001) while it was also significant between G2 and G3 (time-by-group interaction G2*G3, p = 0.004) but not between G1 and G2. Alternatively, MFO expressed in mg.min.kg FFM^-1^ increased significantly in each group (time-by-group interaction, p<0.001), and so defined, this change was larger for G1 than for G2 or G3. Additionally, G1*G2 and G1*G3 time-by-group interactions were both significant (p = 0.016 and p<0.001, respectively) while it was not significant between G2 and G3.

Importantly, we found no correlation between the indicators of insulin resistance and those of MFO.

## Discussion

To the best of our knowledge, in the context of combined lifestyle interventions used for preventing T2DM, the LIPOXmax-Réunion program is the first RCT that aimed at body weight reduction by targeting fat oxidation as the principal fuel of energetic expenditure in a population of young overweight or obese women. All other things being equal *(i*.*e*., baseline inclusion characteristics, baseline exercise data, fruits and vegetables diet), the findings reveal a very significant FM reduction over time with the three training programs, but an absence of superiority of the LIPOXmax intervention to decrease FM despite a better glucose control as indicated by larger decreases of insulin level and HOMA-IR index in this group, as compared with "60% VO2max" and "GPP at-home", the two other competitive programs tested in our trial. It is noteworthy that a five-month exercise regimen guided by the LIPOXmax did not result in superiority despite higher MFO values in the LIPOXmax group.

These outcomes are consistent with the current literature in the field, whose analyses suggest an inconstant benefit of LIPOXmax training. Thus, a recent meta-analysis of 15 studies exploring body composition changes after LIPOXmax training provides evidence that the range of FM reduction varied between 0 and -12.1 kg and that its pooled effect estimate was of −4.1 kg (95% CI: −5.8 to −2.3 kg; p<0.001) [26]. Such heterogeneity in efficacy was first attributed to the various origins of the populations targeted by LIPOXmax RCTs pooled in the meta-analysis. These included people as diverse as T2DM or MS subjects, obese teenagers, or subjects suffering a human immunodeficiency virus infection. Second, in the different RCTs, the implementation of the LIPOXmax program was recognized to vary due to the different durations of intervention, different amounts of weekly sessions, or training combined with diet or not. Thus, beyond theoretical promise, these data still make difficult the interpretation of LIPOXmax RCTs.

In our RCT, the fact of equalizing the endurance intensity in the LIPOXmax and VO2max groups with similar energy expenditures may have contributed to the same magnitude of FM reduction in both groups. Indeed, several trials have shown that isocaloric training groups with different intensities reported similar effects [36–39]. This has led some contributors to conclude that, when matched for energy cost, low- and high-intensity exercises are equally effective in reducing visceral FM. In contrast, low-intensity challenges proved more effective in using lipids and decreasing FM than high-intensity ones [40–42] Thus, the search for a balance between an acceptable energetic cost and the potential for long-term benefits of low-intensity programs, with the aim of targeting more specifically the "substrate of interest", has led experts to explore the concept of "steady state" exercises, which should theoretically allow both similar energy expenditure but higher lipid utilization under various intensities. In this emerging framework, high-intensity steady state (HISS) and also high-intensity intermittent exercises (HIIE) were shown to be more effective than traditional low to moderate steady state exercises (SSE) [43–46]. Interestingly, a recent review suggests that HIIE may have a greater impact on body composition compared with SSE [47]. Hence, one RCT has sought to compare the effects of HIIE and LIPOXmax training programs in T2DM obese patients matched on age, BMI and HbA1c [48]. Its results show evidence that HIIE and LIPOXmax exhibit distinct interesting patterns for DT2 and CVD prevention. On one hand, HIEE improved VO2 peak, reduced systolic blood pressure at rest and total cholesterol. On the other hand, LIPOXmax improved lipid oxidation, decreased FM and HbA1c. Thus the benefits of the two strategies could be synergistic and combined in the management of obese and diabetic subjects. This being said, the optimal intensity of PA should be guided by the parameters we wish to improve (FM loss, FFM increase, glucose control, VO2-peak improvement, control of CVD risk factors).

In our RCT, we observed that FFM decreased in both the LIPOXmax and VO2max groups while it remained stable in the "GPP at-home" control group. We hypothesize that the participants enrolled in the control group may have varied their daily PA practice at points to mobilize other muscle groups to maintain their lean body mass. This was confirmed by the study of PA booklets in the "GPP at-home group" which revealed a good observance of a moderate-intensity and varied PA (i.e., on average more than two types of PA per woman). We believe that this broad diversity of PA may have partially explained the conservation of FFM.

It is also important to emphasise that among the 80% of insulin-resistant (HOMA-IR index > 2.5) obese women at inclusion enrolled in our RCT, a quarter were no longer insulin resistant at the end of the intervention regardless of training program. This finding may be attributed to the benefit of a combined lifestyle intervention whatever the nature of the PA practised, as suggested by the absence of correlation between the level of insulin resistance and values of MFO indicators. However, the improvement in glucose control (measured both on insulin level and HOMA-IR index) and the increase in MFO indicators were much more pronounced in the LIPOXmax group than in the "GPP at-home" group (-6.7 vs -1.6 mUI/L; -1.6 vs -0.3; +72.0 vs +18.6 mg.min^-1^ or +1.7 vs +0.5 mg.min.kg FFM^-1^ respectively for insulin, HOMA-IR index and MFO). Moreover, MFO values expressed in mg.min.kg FFM^-1^ increased more in the LIPOXmax group than in the VO2max group, confirming the expected effect of our targeted intervention on lipid metabolism. Such an improvement in glucose control under the LIPOXmax challenge is in line with previous findings after endurance training in obese or MS subjects [18,22], which is explained by increased lipid oxidation in the muscles of obese subjects during exercise [49]. Subsequently, regular and moderate PA is usually prescribed in obese subjects at-risk of T2DM based on the assumption that lipid oxidation could have a central role in glycemic levels [50].

According to our results, the kinetics is not linear and most of the benefits are obtained within 3 months. In fact, it is not surprising: first, the two periods are not equal, the first one (M0 to M3) being longer than the second one. Then, after initial weight loss, increasing volume of physical activity appears to be important to prevent weight regain [9]. To increase FM loss between M3-M5 it would have been better to adjust PA program in each group (duration and re-evaluation of the adapted intensity).

Finally, based on the lesson issued from the “GPP at-home group”, our findings should encourage the health professionals to continue to prescribe some PA for muscular strengthening purpose, while in turn, the lesson issued from the LIPOXmax group could give an impetus to target a better glucose control through low-intensity exercises in the future studies.

However, in light of new evidence linking the intensity of lipid oxidation to FM loss [51–53], our inconclusive RCT may have some limitations. First, we may have slightly overestimated the precision required in sample size calculation (±2.0 SD) given that we observed dispersion in FM reduction estimates (95% CI, -2.7 to +5.4 kg) between the LIPOXmax and VO2max groups. This has led to a dramatic fall of statistical power from 80% to 33%. This approximation in SD estimation was due to the lack of previously published work evaluating the LIPOXmax program in young, overweight or obese women. Second, the beneficial effect appears to level off at 5 months and most of the gain of LIPOXmax intervention was obtained within 3 months, so that we may think that the duration of the intervention (five months) may have been too short to demonstrate any benefit on FM and a fortiori on fasting glucose profile. Third, five of the women in the VO2max group may indeed have worked in the LIPOXmax zone out of a range of intensities that elicit a minimum of 90% of MFO. These women exhibited a -4.4 kg (±1.4 SD) FM reduction at M5. This observation may have flattened the contrast between LIPOXmax and VO2max groups in FM reduction. A sensitivity analysis excluding these women did not change the overall meaning of our results.

In conclusion, our RCT does not support the superiority of the LIPOXmax training on 60%VO2max or GPP-at-home for FM reduction in young overweight or obese women. Nevertheless, our findings suggest a potential benefit of a combined lifestyle intervention based on LIPOXmax to improve glucose and lipid metabolism. More than ever, deciphering the mechanisms and long-term outcomes of MFO exercises is required before promoting the usefulness of such a strategy in helping to prevent T2DM.

## Supporting Information

S1 CONSORT Checklist (PDF)

S1 Protocol Full protocol in French. (PDF)

S2 Protocol Brief English translation. (PDF)

S1 Table Analysis of bias of selection for baseline inclusion characteristics among participants and non-participants at M5. (DOC)

S2 Table Analysis of bias of selection for exercise tests at inclusion among participants and non-participants at M5. (DOC)

S3 Table Analysis of bias of selection for baseline inclusion characteristics among participants and non-participants at M3 and M5. (DOC)

S4 Table Analysis of bias of selection for exercise tests at inclusion among participants and non-participants at M3 and M5. (DOC)

## Abbreviations

BMI: body mass index
CHO: carbohydrates oxidation
CO2: carbon dioxide production
CVD: cardiovascular diseases
FFM: fat free mass
FM: fat mass
FPG: fasting plasma glucose
GPP: guideline of good physical practices
HbA1c: glycosylated haemoglobin
HIIE: high-intensity intermittent exercises
HISS: high-intensity steady state
HR: heart rate
MAP: maximal aerobic power
MFO: maximal fat oxidation
PA: physical activity
RCT: randomized controlled trial
RER: respiratory exchange ratio
SSE: steady state exercises
T2DM: type-2 diabetes mellitus
VO2: oxygen consumption
WHO: World Health Organization
