# Supplementary material for: Individualized Exercise Training at Maximal Fat Oxidation Combined with Fruit and Vegetable-Rich Diet in Overweight or Obese Women: The LIPOXmax-Réunion Randomized Controlled Trial
Source: PLoS One. 2015 Nov 10;10(11):e0139246. doi: 10.1371/journal.pone.0139246 (PMC4640859; doi:10.1371/journal.pone.0139246)
Supplement: S1 Protocol — (PDF) [file pone.0139246.s002.pdf]

Evaluation de l'efficacité d'un programme d'activité physique individualisé, associé à une alimentation équilibrée enrichie en fruits et légumes, sur l'évolution de la masse grasse chez des femmes en surpoids ou obèses, dans le cadre de la prévention du diabète de type 2 à la Réunion

LIPOXmax-Réunion

Code promoteur : 2011/CHR/01

PROTOCOLE DE RECHERCHE BIOMEDICALE

Version n°2.1 du 10.10.2011

Numéro ID_RCB : 2011-A00687-34
Numéro d'enregistrement dans Clinicaltrials.gov : NCT01464073

Cette recherche biomédicale a obtenu le financement du Contrat de Plan Etat Région

Promoteur :

Centre Hospitalier Régional
Site du Groupe Hospitalier Sud Réunion
97 448 Saint Pierre cedex

Investigateur Principal :

Dr François FAVIER : Centre d'Investigation Clinique - Epidémiologie Clinique, médecin attaché au Service d'endocrinologie – maladies métaboliques – diabétologie. CHR site du GHSR – 97448 Saint Pierre Cedex Tel 0262 35 90 00/54326

Méthodologiste et Centre de Méthodologie et de Gestion des données :

Dr François FAVIER
Adrian FIANU
Centre d'Investigation Clinique - Epidémiologie Clinique de la Réunion

Investigateurs associés

Dr Stéphane SCHNEEBELI : Service d'endocrinologie - maladies métaboliques - diabétologie CHR - GHSR Saint Pierre
Dr Eric JARLET : Service d'endocrinologie - maladies métaboliques - diabétologie CHR - GHSR Saint Pierre
Dr Patrick GERARDIN : Centre d'Investigation Clinique - Epidémiologie Clinique de la Réunion CHR - GHSR Saint Pierre

Unité de sécurité et vigilance de la recherche clinique :

Centre de Pharmacovigilance,
Hôpital Pellegrin CHU
Place Amélie Raba-Léon,
33076 Bordeaux Cedex

HISTORIQUE DES MISES A JOUR DU PROTOCOLE

VERSION	DATE	RAISON DE LA MISE A JOUR
1.0	31.05.2011	Soumission au CPP et à l'Afssaps
2.0	11.08.2011	2ème soumission au CPP : réponses au complément d'information demandé par le CPP
2.1	10.10.2011	Amendement N°1 : Ajout d'une bandelette urinaire à la pré-inclusion, M3 et M5 afin de rechercher une éventuelle grossesse

PRINCIPAUX CORRESPONDANTS

Investigateur Principal
Docteur François Favier
CIC-EC et Service d'endocrinologie,
maladies métaboliques, diabétologie du GHSR
GHSR BP 350
97448 St Pierre Cedex
Tél. : 02 62 35 90 00/54326
Fax : 02 62 35 93 78
Courriel : francois.favier@chr-reunion.fr

Florent Besnier
Professeur en Activités Physiques Adaptées (APA)
Chargé de projets
CIC-EC de la Réunion
CHR – GHSR BP 350
Saint Pierre

Chef de Projets - Promoteur
Fidéline Filleul
Tél : 02 62 90 62 84 - Fax : 02 62 90 69 21
Courriel : fideline.filleul@chr-reunion.fr

Promoteur
Centre Hospitalier Régional - Site du GHSR
97 448 Saint Pierre cedex

Responsable de la recherche au niveau du promoteur
Joaquin Martinez
Directeur Adjoint chargé de la Recherche
Tél : 02 62 71 98 52 Fax : 02 62 71 98 34
joaquin.martinez@chr-reunion.fr

Attachée de Recherche Clinique Manager
Vanessa BASQUE
CHR de la Réunion – Site du GHSR
Bd François Mitterrand – BP 350
97448 Saint Pierre Cedex
Tél. : 02 62 35 90 00 (Poste 54813) - Fax : 02 62 35 97 21
vanessa.basque@chr-reunion.fr

Unité de sécurité et vigilance de la recherche clinique :
Centre de Pharmacovigilance,
Hôpital Pellegrin CHU
Place Amélie Raba-Léon,
33076 Bordeaux Cedex
Tel : 05 57 57 46 64 - Fax : 05 56 79 49 26
anne.gimbert@pharmaco.u-bordeaux2.fr
francoise.haramburu@u-bordeaux2.fr
ghada.miremont@u-bordeaux2.fr

Partenaires scientifiques de la recherche

Marie-Paule Gonthier
MCF Université de la Réunion
Laboratoire de Biochimie et
Génétique Moléculaire (LBGM)
groupe GEICO
Moufia Saint Denis

Jean Frédéric Brun
PH Centre d'Exploration et de
Réadaptation des Anomalies
Musculaires et Métaboliques
(CERAMM) CHU Lapeyronie
Montpellier

Mathieu Desplan
AHU Unité CERAMM
CHU Lapeyronie
Montpellier

Chantal Verkindt
MCF Université de la Réunion
Centre Universitaire de Recherche
en Activité Physique et Sportive (CURAPS)
équipe Dimps EA 4075
Le Tampon

Christian Lefebvre d'Hellencourt
MCF Université de la Réunion
LBGM groupe GEICO
Moufia Saint Denis

Georges Dalleau
Professeur Université de la Réunion
CURAPS équipe Dimps EA 4075
Le Tampon

Jacques Mercier
PU-PH Unité CERAMM
CHU Lapeyronie
Montpellier

Centre de Méthodologie et de Gestion des données
Dr François Favier méthodologiste
Adrian Fianu statisticien
Karim Boussaïd data manager
Nadège Naty coordinatrice d'enquête
CIC-EC de la Réunion

SOMMAIRE

1. RESUME DE LA RECHERCHE **8**
ABSTRACT **12**
2. JUSTIFICATION SCIENTIFIQUE ET DESCRIPTION GENERALE **14**
2.1. ETAT ACTUEL DES CONNAISSANCES 14
2.1.1. Sur la pathologie 14
2.1.2. Sur les procedures de référence et à l'etude 16
2.2. JUSTIFICATION DES CHOIX METHODOLOGIQUES 18
2.3. PROBLEMATIQUE 19
2.4. HYPOTHESES DE LA RECHERCHE ET RESULTATS ATTENDUS 20
2.5. RAPPORT BENEFICE / RISQUE 20
2.6. RETOMBEES ATTENDUES 20
3. OBJECTIFS DE LA RECHERCHE **21**
3.1. OBJECTIF PRINCIPAL 21
3.2. OBJECTIFS SECONDAIRES 21
4. CONCEPTION DE LA RECHERCHE **21**
4.1. SCHEMA DE LA RECHERCHE 21
4.2. METHODES POUR LA RANDOMISATION 21
5. CRITERES D'ÉLIGIBILITE **22**
5.1. CRITERES D'INCLUSION 22
5.2. CRITERES DE NON INCLUSION 22
5.3. MODALITES DE RECRUTEMENT 23
6. PROCEDURE DE LA RECHERCHE **23**
6.1. PROCEDURE A L'ETUDE 23
6.2. PROCEDUREs DE COMPARAISON 23
6.3. PRODUITS ALIMENTAIRES (FRUITS ET LEGUMES) 24
7. CRITERES DE JUGEMENT **24**
7.1. CRITERE DE JUGEMENT PRINCIPAL 24
7.2. CRITERES DE JUGEMENT SECONDAIRES 25
8. DEROULEMENT DE LA RECHERCHE **25**
8.1. CALENDRIER DE LA RECHERCHE 25
8.2. TABLEAU RECAPITULATIF DU SUIVI PATIENT 26
8.3. RECRUTEMENT DES PERSONNES 28
8.4. VISITE DE PRE-INCLUSION 28
8.5. VISITE/DEMARCHE DE RANDOMISATION 29
8.6. VISITE D'INCLUSION 29
8.7. VISITES DE SUIVI M_3 29
8.8. VISITE DE SUIVI M_5 30
8.9. VISITE DE FIN DE LA RECHERCHE 30

8.10. REGLES D'ARRET DE LA RECHERCHE 30
8.11. INDEMNISATION EVENTUELLE DES SUJETS 31
8.12. COLLECTION BIOLOGIQUE 31

9. GESTION DES ÉVÉNEMENTS INDÉSIRABLES ET DES FAITS NOUVEAUX 31

9.1. DEFINITIONS 31
9.2. DESCRIPTION DES EVENEMENTS INDESIRABLES GRAVES ATTENDUS 32
9.3. CONDUITE A TENIR EN CAS D'EVENEMENT INDESIRABLE OU DE FAIT NOUVEAU 32
9.4. DECLARATION ET ENREGISTREMENT DES EIG INATTENDUS ET DES FAITS NOUVEAUX 33

10. ASPECTS STATISTIQUES 33

10.1. CALCUL DE LA TAILLE D'ETUDE 33
10.2. METHODES STATISTIQUES EMPLOYEES 34

11. SURVEILLANCE DE LA RECHERCHE 35

11.1. CONSEIL SCIENTIFIQUE 35
11.1.1. Composition 35
11.1.2. Rythme des réunions 35
11.1.3. Rôle 35
11.2. CENTRE DE METHODOLOGIE ET DE GESTION DES DONNEES 36
11.2.1. Composition 36
11.2.2. Rythme des réunions 36
11.2.3. Rôle 36
11.3. CENTRE INVESTIGATEUR COORDONNATEUR 36

12. DROITS D'ACCES AUX DONNEES ET DOCUMENTS SOURCE 37

12.1. ACCES AUX DONNEES 37
12.2. DONNEES SOURCE 37
12.3. CONFIDENTIALITE DES DONNEES 37

13. CONTROLE ET ASSURANCE QUALITE 38

13.1. CONSIGNES POUR LE RECUEIL DES DONNEES 38
13.2. SUIVI DE LA RECHERCHE 38
13.3. CONTROLE DE QUALITE 38
13.4. GESTION DES DONNEES 38
13.5. AUDIT ET INSPECTION 39

14. CONSIDERATIONS ETHIQUES ET REGLEMENTAIRES 39

15. CONSERVATION DES DOCUMENTS ET DES DONNEES RELATIVES A LA RECHERCHE 40

16. REGLES RELATIVES A LA PUBLICATION 41

16.1. COMMUNICATIONS SCIENTIFIQUES 41
16.2. COMMUNICATION DES RESULTATS AUX PATIENTS 41
16.3. CESSION DES DONNEES 41

REFERENCES BIBLIOGRAPHIQUES 42

ANNEXES 49

ANNEXE 1 : CENTRE INVESTIGATEUR 49
ANNEXE 2 : NOTE D'INFORMATION ET FORMULAIRE DE CONSENTEMENT 50
ANNEXE 3 : INFORMATION DANS LA PRESSE 56

PAGE DE SIGNATURE DU PROTOCOLE

«Evaluation de l'efficacité d'un programme d'activité physique individualisé, associé à une alimentation équilibrée enrichie en fruits et légumes, sur l'évolution de la masse grasse chez des femmes en surpoids ou obèses, dans le cadre de la prévention du diabète de type 2 à la Réunion»

LIPOXmax-Réunion

Code promoteur : 2011/CHR/01

Promoteur

Centre Hospitalier Régional
Site du Groupe Hospitalier Sud Réunion
97 448 Saint Pierre cedex

à Saint Pierre, le : 31.05.2011

Le Directeur Général du CHR La Réunion
Michel CALMON
Et par délégation, le Directeur Adjoint chargé de la Recherche

Joaquin MARTINEZ

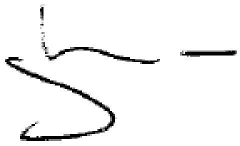

Investigateur principal

François Favier
Centre d'Investigation Clinique
Centre Hospitalier Régional
Site du Groupe Hospitalier Sud Réunion BP 350
97448 Saint Pierre Cedex
La Réunion
Tel : 02 62 35 90 00/59829
Fax : 0262 35 93 78
Courriel : francois.favier@chr-reunion.fr

à Saint Pierre, le : 31.05.2011

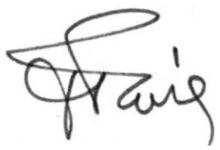

LISTE DES ABREVIATIONS

FC : fréquence cardiaque (batt.min^{-1}).

VO$_{2max}$: débit maximal d'O_2 qui peut être consommé par l'organisme (L.min^{-1}).

LIPOXmax : débit maximal d'oxydation des lipides (mg.min^{-1}).

PC : point de croisement des substrats énergétiques ou *cross over concept*.

RBP : recommandations de bonnes pratiques en activité physique.

PNNS : Programme National Nutrition Santé

RAE : réentraînement à l'effort.

1. RESUME DE LA RECHERCHE

**PROMOTEUR**	Centre Hospitalier Régional<br>Site du Groupe Hospitalier Sud Réunion<br>97 448 Saint Pierre cedex
**INVESTIGATEUR PRINCIPAL**	François FAVIER<br>CIC-EC de la Réunion<br>Service d'endocrinologie –<br>maladies métaboliques – diabétologie<br>Centre Hospitalier Régional<br>Site du Groupe Hospitalier Sud Réunion<br>BP 350 97448 Saint Pierre Cedex<br>Tel : 0262 35 90 00/54326<br>Fax : 0262 35 93 78<br>Courriel : francois.favier@chr-reunion.fr
**TITRE**	Evaluation de l'efficacité d'un programme d'activité physique individualisé, associé à une alimentation équilibrée enrichie en fruits et légumes, sur l'évolution de la masse grasse chez des femmes en surpoids ou obèses, dans le cadre de la prévention du diabète de type 2 à la Réunion
**JUSTIFICATION / CONTEXTE**	A la Réunion, en 2000 l'enquête Rédia a montré une prévalence du diabète de type 2 chez les 30-69 ans supérieure à 17%, une prévalence de l'obésité de 20% chez les femmes et de 10% chez les hommes. Cette prévalence élevée chez les femmes justifie de mettre en place un essai d'intervention les concernant ; dans notre expérience à la Réunion, les femmes participent plus volontiers que les hommes à la recherche, et ont une meilleure observance. Enfin, dans un essai d'intervention sur le surpoids et l'obésité chez l'adulte, basé sur l'activité physique, il est préférable d'inclure des patientes âgées de 20 à 40 ans, stables sur le plan anthropométrique. Selon les données de la littérature traitant des stratégies de prévention et/ou de traitement des maladies métaboliques, l'activité physique régulière associée à une alimentation équilibrée et un apport de fruits et légumes, permet de diminuer la masse grasse et les complications de l'obésité, complications qui sont en partie liées à l'état inflammatoire et au stress oxydatif qui accompagnent l'obésité. Cependant la prescription d'une activité physique régulière visant à favoriser la perte de poids et l'amélioration des paramètres biologiques qui sont liés à l'obésité, n'est généralement pas individualisée et ne fait pas l'objet d'un consensus sur l'intensité de l'activité physique. Les recommandations internationales recouvrent une large gamme d'intensité d'exercice (entre 40% et 60 % de la consommation maximale d'oxygène). Le groupe de Jacques Mercier (CERAMM-CHU Lapeyronie à Montpellier) a montré l'intérêt de l'entraînement effectué à une intensité d'effort relativement basse, correspondant au débit maximal d'oxydation lipidique (LIPOXmax), et ses effets sur la masse grasse, la sensibilité à l'insuline et le métabolisme musculaire, chez des personnes en surpoids ou obèses
**OBJECTIFS**	Objectif principal : évaluer l'efficacité à 5 mois de trois programmes de réentraînement à l'effort associés à une alimentation équilibrée supplémentée en fruits et légumes, sur la masse grasse chez des femmes de 20-40 ans en surpoids ou obèses.<br>Objectifs secondaires : étudier les effets sur le stress-oxydatif et le statut inflammatoire, comparer l'efficacité des 3 programmes de réentraînement sur les modifications de la composition corporelle, la sensibilité à l'insuline, l'utilisation des lipides à l'effort, la qualité de vie, décrire la faisabilité en population, l'observance.

**SCHEMA DE LA RECHERCHE**	Essai d'intervention, de supériorité, sans insu randomisé en trois groupes parallèles : bras 1 intensité d'effort LIPOXmax, bras 2 intensité d'effort 60% du VO_{2max}, bras 3 intensité d'effort selon les Recommandations des Bonnes Pratiques (30 minutes à intensité modérée), avec alimentation équilibrée supplémentée en fruits et légumes dans les trois bras.
**CRITERES D'INCLUSION**	- Femmes<br>- Agées de 20 à 40 ans<br>- 27 ≤IMC ≤ 40 kg/m²<br>- Glycémie < 1,26g/l ;<br>- HbA1c < 6,5%<br>- Considérées comme cliniquement stables<br>- Aptes à un programme de réentraînement à l'effort<br>- Sédentaires (moins de 2 heures d'activités physiques par semaine)<br>- Patientes ayant signé un consentement de participation à l'étude<br>- Sans activité professionnelle<br>- Affiliées à un régime de Sécurité Sociale
**CRITERES DE NON INCLUSION**	- HTA sévère (PAS ≥180 mmHg et/ou PAD ≥110 mmHg)<br>- Patiente hypertendue (≥140/90 mmHg) non contrôlée par un traitement médical<br>- Patiente hypertendue dont le traitement médical comprend des bétabloquants ou des inhibiteurs calciques<br>- Contre-indication absolue et relative à l'épreuve d'effort (ACC/AHA, 2002) et/ou à l'entraînement physique<br>- Incapacité de réaliser l'épreuve d'effort maximale et/ou l'épreuve d'effort métabolique<br>- Pathologie cardiovasculaire et/ou respiratoire non équilibrée révélée par l'épreuve d'effort<br>- Port de stimulateur ou de défibrillateur cardiaque<br>- Troubles du rythme ventriculaire sévères non stabilisés sous traitement<br>- Evènements cardio-vasculaires récents (décompensation cardiaque ou traitement par drogues inotropes positives ou angioplastie, datant de moins de 10 jours, chirurgie cardiaque datant de moins de 3 mois, maladie valvulaire nécessitant une correction chirurgicale, myopéricardite en évolution, troubles du rythme ventriculaire sévères non stabilisés sous traitement)<br>- Myopathie connue et documentée<br>- Affections cancéreuses<br>- Maladie aiguë et chronique inflammatoire<br>- Insuffisance rénale chronique<br>- Patientes opérées du tube digestif<br>- Pathologie associée évolutive entraînant une altération importante de l'état général<br>- Patiente sous corticoïdes, hormones thyroïdiennes, antidépresseurs ou neuroleptiques<br>- Grossesse en cours ou désir de grossesse avant la visite de fin d'étude.<br>- Déficit mental empêchant la compréhension du consentement éclairé et des protocoles<br>- Participation à un autre protocole de recherche<br>- Participation dans le mois précédent à un programme de réentraînement à l'effort ou suivi d'un régime amaigrissant.

**PROCEDURES DE LA RECHERCHE**	Toutes les participantes auront une éducation nutritionnelle initiale et bénéficieront d'une supplémentation gratuite en fruits et légumes à raison de 5 par jour tout au long de l'essai.<br>A l'issue du bilan d'inclusion, les participantes seront randomisées en trois groupes :<br>**Groupe 1 :** groupe LIPOXmax. Les sujets réaliseront une activité physique à raison de 4x1 heure par semaine selon l'intensité optimale pour l'oxydation des lipides (LIPOXmax) mesurée par calorimétrie indirecte.<br>**Groupe 2 :** groupe activité physique standard (60% du VO_{2max} mesuré par épreuve d'effort cardiorespiratoire maximale). Les sujets réaliseront 4 séances par semaine et la durée des séances devra être ajustée de manière à ce que les groupes 1 et 2 aient la même dépense énergétique totale par séance.<br>**Groupe 3 :** groupe témoin. Les sujets recevront initialement les recommandations de bonnes pratiques médicales pour l'activité physique (réaliser au minimum 3h30min d'exercice par semaine a une intensité dite « modérée »).<br>La durée du suivi de l'essai est de 5 mois au total ; pour les groupes 1 et 2 : les activités physiques se dérouleront en salle sur vélo ergométrique contrôlées par un éducateur sportif, avec l'objectif d'une autonomisation progressive; pour le groupe 3 : l'activité physique sera contrôlée par un suivi téléphonique régulier (bimensuel), tenue d'un cahier d'activités physiques avec les caractéristiques de chaque séance et port d'un podomètre lors des séances de marche.
**CRITERES DE JUGEMENT**	**Critère de jugement principal.** L'efficacité de l'intervention sera jugée sur l'évolution entre M_0 et M_5 de la masse grasse, en kilogrammes, mesurée par absorptiométrie biphotonique.<br>**Critères de jugement secondaires de l'étude.** L'efficacité de l'intervention sera également jugée sur l'évolution entre M_0 et M_5 :<br>- du statut inflammatoire et antioxydant et autres paramètres biologiques (glycémie, HbA1c, insulinémie, cholestérolémie, HDL/LDL, triglycéridémie).<br>- de la composition corporelle (poids, IMC, tour de taille, tour de hanches, masse musculaire)<br>- de l'utilisation des lipides à l'effort<br>- de la qualité de vie<br>- du recueil des éventuels effets secondaires (douleurs, fatigue,…)<br>- de la faisabilité en population (adhésion), et de l'observance.
**TAILLE D'ETUDE**	126 sujets répartis en trois groupes de 42 sujets chacun. Pour mettre en évidence une différence d'évolution moyenne de masse grasse de 1,5 kg avec un écart-type de 2,0 kg, un risque de première espèce de 1,67 % (5% / 3), une puissance de 80%, il est nécessaire d'inclure en situation bilatérale 38 sujets par groupe. En raison d'un taux de données manquantes (abandon, perdus de vue) estimé à 10%, il est prévu d'inclure 42 sujets par groupe, soit 126 au total.
**NOMBRE PREVU DE CENTRES**	étude monocentrique
**DUREE DE LA RECHERCHE**	Inclusion : 2 mois<br>Durée de participation de chaque personne : 5 mois<br>Saisie des données, analyse et publication(s) : 17 mois<br>Durée totale de la recherche : 2 ans

**ANALYSE STATISTIQUE DES DONNEES**	Analyse unique, en intention de traiter (selon le groupe attribué par la randomisation). Risque global de première espèce = 5 %. Formulation bilatérale des tests statistiques. Méthodes statistiques utilisées : en analyse bivariée (ANOVA à 1 facteur, test du Chi2 de Pearson ou test exact de Fisher), en analyse multivariée (ANCOVA, régression logistique). Représentation graphique des évolutions sur la durée de suivi. Test des tendances après prise en compte du caractère longitudinal des données (M_0-M_3-M_5) par équations d'estimation généralisées (EEG).
**RETOMBEES ATTENDUES**	Les retombées attendues sont :<br>-la mise en place d'un programme de prévention pérenne visant à favoriser la consommation de fruits et légumes et une activité physique régulière individualisée, prescrite et contrôlée.<br>-la démonstration de l'intérêt et de la faisabilité de la prescription individualisée d'une activité physique.<br>Ce projet de recherche-action insiste, notamment au cours des 2 derniers mois, sur la transmission de connaissances et compétences relatives à la gestion de son activité physique (utilisation des cardiofréquencemètres, principes d'échauffement, d'hydratation…). De sorte que les sujets participants à l'étude soient autonomes en fin d'étude (M_5) et deviennent les relais d'information sur l'activité physique adaptée, dans leur entourage.<br>Nous envisageons une diffusion au niveau régional des résultats de l'étude et du programme d'activité physique qui se montrera le plus efficace : médecins, institutions de santé publique, associations de lutte contre l'obésité, le diabète et les maladies cardio-vasculaires, réseaux de prévention …<br>-la démonstration de l'intérêt et de la possibilité de supplémenter en fruits et légumes l'alimentation des réunionnais, et la facilitation de l'observance de cette consommation lorsqu'il n'y a pas de barrière financière (fruits et légumes fournis gratuitement pendant l'essai).<br>Les retombées sont pour la santé mais également sur le plan de l'économie locale : intérêt de développer l'agriculture vivrière, les circuits courts, les jardins familiaux, l'information sur les fruits et légumes.

ABSTRACT

This research has been registered in http://www.clinicaltrials.gov/ the *date* under the n° *numéro.*

Evaluation of the effectiveness of individualized exercise program, combined with a balanced diet rich in fruits and vegetables, on the evolution of body fat in overweight or obese women, as part of Type 2 diabetes prevention in La Réunion island : LIPOXmax-Réunion

CHR de la Réunion is the sponsor of this research

This research will be conducted with the support of CPER

Background: in Reunion Island, in 2000 the REDIA study showed a prevalence of type 2 diabetes among 30-69 years old people above 17%, a prevalence of obesity among women of 20% and 10% for men. According to the literature upon preventive strategies and/or treatment of metabolic diseases, regular physical activity associated with a balanced diet and an intake of fruits and vegetables, can reduce body fat mass and prevent the complications related to obesity. These complications are partly related to the inflammatory and oxidative stress that accompanies obesity. A diet rich in fruits and vegetables, may improve the inflammatory and oxidative status. However there is no consensus on the intensity of regular physical activity in the practices of prevention and care. These parameters are generally not individualized. International recommendations cover a wide range of exercise intensity (between 40% and 60% of maximal oxygen uptake). The research group of Jacques Mercier (CERAMM Lapeyronie-CHU Montpellier) showed the benefit of the training conducted at a relatively low intensity of effort, corresponding to the maximum rate of lipid oxidation (LIPOXmax), and its effects on the fat mass, the insulin sensitivity and the muscle metabolism, in overweight or obese people.

Main objective: to study the effects of three programs of rehabilitation training associated with a diet supplemented with fruits and vegetables, on reducing body fat in overweight or obese people.

Secondary objectives: to study the effects on the oxidative stress and the inflammatory status, compare the efficacy of these 3 programs of physical training on body composition modifications, insulin sensitivity, lipid utilization during exercise and quality of life, describe feasibility in population (compliance, motivation).

Abstract: according to data from the literature, a balanced diet combined with moderate exercise helps to fight against biological complications related to obesity and to reduce the incidence of type 2 diabetes by promoting fat loss. But there is no consensus on the intensity of physical activity prescribed. Have been shown the benefits of training performed at an individualized intensity of effort, relatively low, corresponding to the maximum rate of lipid oxidation measured by indirect calorimeter (LIPOXmax) on fat loss, sensitivity to insulin and muscle metabolism in obese or diabetic patients. However, an individualized LIPOXmax exercise training has not yet been compared to a standardized training conventionally used in the treatment of obese subjects. Furthermore, no study has yet evaluated the impact of LIPOXmax training on inflammatory and antioxidant status among overweight and obese patients. We propose to compare among overweight or obese patients, the effects of an individualized training at an intensity corresponding to LIPOXmax, the effects of a standardized training to 60% of VO_{2max}, and the effects of a moderate and regular physical activity prescribed according to guidelines of Good Medical Practices.

At M0 participants will have anthropometric and functional examinations and laboratory tests, and will follow nutritional education sessions. They will be then randomized into 3 arms: arm 1 LIPOXmax physical activity, arm 2 60% VO_{2max} physical activity, arm 3 physical activity according to guidelines of Good Medical Practices. Participants of the arms 1 and 2 will practice under the control of a sports instructor; participants of the arm 3 will be regularly monitored by telephone. The procedure will last 5 months. At M3 and M5 anthropometric and biological examinations will be replicated and participants will answer to questionnaires on quality of life and experience of physical activity during and after the intervention.

Primary outcome: body fat mass reduction, in kilograms and in % of total weight.

Secondary outcomes: improvement of biological parameters (markers of inflammation and antioxidant status, blood glucose, HbA1c, insulin, cholesterol, HDL / LDL, triglycerides); improvement in body

composition (weight, BMI, waist circumference, hip circumference, muscle mass), and in lipid utilization during exercise; improvement of the quality of life; demonstration of the feasibility in population (adherence, compliance).

Study design: controlled, randomized, of superiority, not blind trial in 126 people divided into three arms of 42 people per arm: arm 1 LIPOXmax intensity of effort, arm 2 60% of VO_{2max} intensity of effort, arm 3 intensity of effort according to the Guidelines of Good Medical Practices.

Inclusion criteria: women, 20-40 years old, 27 ≤ BMI ≤40 kg / m², sedentary, considered clinically stable, able to rehabilitation training, having signed an informed consent, without professional activity.

Exclusion criteria: blood glucose ≥ 1.26 g / l, HbA1c ≥ 6.5%, severe hypertension (SBP ≥ 180 mmHg and / or DBP ≥ 110 mmHg), hypertension (≥140/90 mmHg) untreated or treated by beta blocker or calcium blocker, absolute and relative contraindication to the maximal exercise test (ACC / AHA 2002) and / or physical training, inability to achieve the maximum exercise test and / or the metabolic exercise test by indirect calorimeter, uncompensated cardiovascular and / or respiratory disease revealed by exercise test, pacemaker or defibrillator, recent cardiovascular events (heart failure, treated by positive inotropic drugs, angioplasty within the last 10 days, cardiac surgery within the last 3 months, valvular disease requiring surgical correction, evolving myopericarditis, severe ventricular arrhythmias non stabilized under treatment), known and documented myopathy, cancer, acute and chronic inflammatory disease, end stage renal disease, digestive system operation, treated by corticoids, thyroid hormone, antidepressant or neuroleptics, pregnancy, mental deficiency that prevents the understanding of informed consent and protocol, participation to another research protocol, attendance in the previous month to a program of rehabilitation training or a diet, associated evolutionary disease causing significant impairment of general condition.

Intervention: the 126 subjects will undergo the following exams: weight, height, waist and hip circumference, blood pressure, body fat measurement by impedancemetry and biphotonic absorptiometry, maximal cardiopulmonary exercise and effort metabolic test (by indirect calorimeter to determine the LIPOXmax and the crossing points of the substrates), laboratory tests (blood glucose, HbA1c, insulin, cholesterol total, HDL, LDL, triglycerides, CRP, albumin, creatinine, urinary parameters, antioxidant status, inflammatory markers); questionnaires about medical history and sociodemographic data; food survey (frequency questionnaire, quantization by photos), questionnaires on physical activity (Baecke amended), quality of life (SF-36), experience of physical activity. All the participants will have an initial nutritional education session and will receive a free supplement of 5 fruits and vegetables per day during all the intervention, and random urine assays will be performed to monitor the consumption of fruits and vegetables. Participants will be randomized into three arms. Arm 1: LIPOXmax arm. Subjects will perform a physical activity 4x1 hour per week at the optimal intensity for fat oxidation (LIPOXmax) measured by indirect calorimeter. Arm 2: physical activity standard arm (60% of VO_{2max} measured by cardio respiratory exercise test). Subjects will realize 4 sessions per week and duration of sessions will be adjusted so that arms 1 and 2 have the same total energy expenditure by session. Arm 3: control arm. The subjects initially receive the recommendations of good medical practice for physical activity (achieving at least 3h30min per week of "moderate" exercise). The intervention will last 5 months in total. For arms 1 and 2: physical activity on ergo meter bicycle controlled by a sports trainer, with the goal of gradual empowerment. For arm 3: self-directed activities with regular telephone follow-up, holding of a book on physical activities and use of a pedometer during walking sessions. Indemnization: all the participants will receive a monthly allowance for the constraints due to their participation.

Number of subjects: to highlight a difference in average change of 1.5 kg fat mass, with a standard deviation of 2.0 kg, a first species risk of 1.67% (5% / 3), a power of 80%, it is necessary to include 38 subjects. In such circumstances it will be possible to demonstrate a theoretical minimum significant difference between arms of: - 2.3% on changes at 5 month of average percentage of body fat (SD = 3%) and - 3.8 kg on changes at 5 months of average weight (SD = 5 kg). Due to missing data (dropouts, lost) rate estimated at 10%, it is planned to include 42 subjects per arm, or 126 in total.

Statistical analysis: single analysis in intention to treat (arms assigned by the randomization). Overall first species risk = 5%. Bilateral formulation of statistical tests. Statistical methods used: bivariate analysis (one factor ANOVA, Pearson Chi2 test or Fisher test), multivariate analysis (ANCOVA, logistic regression). Taking into account the longitudinal data (M0-M3-M5) with generalized estimating equations (GEE). Graphic representation of the evolutions over the study.

2. JUSTIFICATION SCIENTIFIQUE ET DESCRIPTION GENERALE

2.1. ETAT ACTUEL DES CONNAISSANCES

2.1.1. SUR LA PATHOLOGIE

Le diabète : épidémiologie et facteurs de risques

Le diabète représente une véritable épidémie mondiale qui concerne près de 220 millions de personnes dont plus de 90% de diabète de type 2 (DT2). En 2025, l'Organisation Mondiale de la Santé (OMS) estime que 330 millions de personnes en seront atteintes. En France, la prévalence du diabète diagnostiqué est de 3,3% soit environ 2 millions de patients traités. L'accroissement en France de la prévalence est de 3,2% par an (Ricci et al. 2010). Les départements et les territoires français d'outre mer ne sont pas épargnés bien au contraire. A la Réunion par exemple, l'enquête REDIA a revélé une prévalence supérieure à 17% chez les 30-69 ans (Favier et al. 2005). Les mécanismes en cause, l'insulinorésistance, l'insuffisance d'utilisation musculaire des acides gras et du glucose, sont associés au surpoids ou à l'obésité, à l'obésité abdominale, à l'hypertension artérielle et aux autres composantes du syndrome métabolique (Riccardi et al. 2004).

Les principaux facteurs de risques de DT2 mis en évidence par les études de cohorte sont les facteurs environnementaux avec une alimentation déséquilibrée riche en graisses saturées et en sucres rapides et pauvre en fruits et légumes, et la sédentarité. Le surpoids ou l'obésité qui en résultent induisent un dysfonctionnement des cellules adipeuses diminuant progressivement la sensibilité à l'insuline (Regensteiner et al. 1991, Kriska et al. 1993, Schulz et al. 2006).

L'obésité : définition et épidémiologie

Reconnue comme maladie chronique depuis 1997 par l'Organisation Mondiale de la Santé (OMS), la définition de l'obésité repose sur la mesure de l'indice de masse corporelle (IMC), calculé en divisant le poids en kilogrammes par le carré de la taille en mètre. A partir de cet indicateur, l'OMS a défini des seuils de surcharge pondérale (IMC compris entre 25 et 30 kg/m²) et d'obésité (IMC supérieur à 30 kg/m²).

Selon les résultats de l'enquête ObEpi (2009), la prévalence de l'obésité augmente en France et pourrait atteindre 20% en 2020. D'après cette enquête, la prévalence de l'obésité est plus importante chez les femmes (15,1 %) que chez les hommes (13,9 %). A la Réunion, l'enquête REDIA a montré une prévalence de l'obésité de 20% chez les femmes et de 10% chez les hommes expliquant vraisemblablement la prévalence élevée du DT2 (Favier et al. 2005).

Physiopathologie de l'obésité

L'excès d'apport énergétique entraîne un état de stress dans le tissu adipeux, qui aboutit à la dérégulation des fonctions de stockage et de sécrétions des cellules adipeuses. Ce dysfonctionnement est à l'origine de pathologies graves comme le DT2 et les maladies cardiovasculaires qui sont actuellement la première cause de mortalité en France (Després 2007, Arsenault et al. 2010). Ce dysfonctionnement se traduit par trois évènements majeurs :

1-une hypertrophie et une hyperplasie des cellules adipeuses (Van Harmelen et al. 2003, Drolet et al. 2008) qui s'observe en premier lieu dans le tissu adipeux sous-cutané puis au niveau viscéral (Drolet et al. 2008). C'est cet excès de masse viscérale qui est particulièrement délétère car il va perturber la signalisation intra-adipocytaire et diminuer la sensibilité à l'insuline (Houstis et al. 2006, Veilleux et al. 2011). L'insulino-résistance s'accompagne alors d'une profonde altération du stockage des graisses au niveau du tissu adipeux qui aboutit à des dépôts graisseux ectopiques d'une part au niveau des muscles squelettiques et du foie aggravant le statut hyperglycémique et hyperlipidique lié à l'obésité, et d'autre part au niveau du muscle cardiaque et des parois vasculaires, à l'origine des maladies cardiovasculaires (Després & Lemieux 2006, Arnlöv et al. 2010).

2-un stress oxydatif : l'excédent de substrats énergétiques va également provoquer une série de « stress » intracellulaires, en particulier un stress oxydatif au niveau mitochondrial (de Ferranti & Mozaffarian 2008).

Le stress oxydatif, défini comme un déséquilibre entre le niveau d'espèces réactives de l'oxygène et de l'azote (ERDONs) et le potentiel antioxydant d'une cellule, est généré de plusieurs façons dans l'adipocyte du sujet obèse : par l'accélération de l'oxydation des substrats énergétiques au niveau de la chaîne mitochondriale (Lin et al. 2005), par l'auto-oxydation du glucose en excès (Harding & Beswick 1988), l'hypoxie induite par l'hypertrophie (Carrière et al. 2004), l'activation d'enzymes pro-oxydants membranaires (Krieger-Brauer et al. 2000).
L'excès de molécules oxydantes qui en résulte, sous forme d'espèces réactives de l'oxygène (H_2O_2, OH°, O2°$^-$), provoque des dommages oxydatifs de cibles majeures comme les protéines, l'ADN, le glucose ou les lipides membranaires.
Ceci contribue notamment au dépôt lipidique de la plaque d'athérome des maladies cardiovasculaires et à la glycation de protéines telles l'hémoglobine et l'albumine, marqueurs oxydatifs associés au DT2 (Kennedy & Lyons1997 ; Chesne et al. 2006).
3-un état d'inflammation chronique : l'excès de lipides au niveau du tissu adipeux engendre également un stress au niveau du réticulum endoplasmique des adipocytes. Ceci se traduit par une altération des synthèses protéiques et la sécrétion d'adipokines par les adipocytes (Gregor & Hotamisligil 2007) avec diminution de la sécrétion de molécules anti-inflammatoires comme la leptine et l'adiponectine et l'augmentation de la sécrétion de molécules pro-inflammatoires. L'obésité s'accompagne d'une large réaction inflammatoire caractérisée par la surproduction de diverses molécules telles que l'Interleukine-6 (IL-6), le Tumor Necrosis Factor-alpha (TNF-alpha) et d'autres médiateurs tels que les endocannabinoïdes dérivant des lipides alimentaires, qui entretiennent de manière significative l'insulino-résistance, élément principal du DT2 (Kern et al. 1995, Matias et al. 2006, Gonthier et al. 2007, Boden 2011).

La sédentarité favorise le développement de l'obésité
La sédentarité, ou l'inactivité physique, correspond à moins de 30 minutes d'activité physique modérée par jour, c'est-à-dire une activité provoquant un essoufflement modéré (Carré F. 2005). D'après le rapport de l'INPES (Baromètre Santé 2005), en France, moins de la moitié des adultes déclarent avoir marché pendant au moins trente minutes, au moins cinq jours la semaine précédente. A la Réunion, seulement 5% des personnes interrogées lors de l'enquête RECONSAL, réalisée chez 1061 personnes, cumulent les activités sportives, professionnelles et domestiques (Favier et al. 2001). Cette enquête a mis en évidence une association significative entre l'obésité abdominale et l'absence d'activité physique. Les relations entre les comportements sédentaires et le développement de l'obésité sont multiples. Elles pourraient s'expliquer entre autre par une diminution de la dépense énergétique et une augmentation des apports alimentaires, entraînant un déséquilibre de la balance énergétique entre les apports et les dépenses (Harnack et al. 2000). En effet, les comportements sédentaires tels que regarder la télévision, sont associés à un risque significativement plus élevé de développer une obésité (Rey Lopez et al. 2008). Une étude portant sur 50 277 femmes suivies pendant 6 ans souligne que 2 heures par jour passées devant la télévision augmente le risque d'obésité de 23% (Hu et al. 2003). L'obésité, tout comme la sédentarité, contribuent indépendamment l'une de l'autre au développement du DT2. Dans la cohorte de la *Nurses'Health Study* où 68 907 femmes sans antécédent de diabète ont été suivies pendant 16 ans (Rana et al. 2007), le risque de DT2 augmente progressivement avec l'augmentation de l'IMC, du tour de taille et avec la diminution de l'activité physique. Le risque relatif est multiplié par 17 pour les femmes obèses et sédentaires, de 11 pour les femmes obèses ayant une activité physique régulière, et de 2 pour les femmes minces et sédentaires (par rapport à un groupe référent composé de femmes dont l'IMC est inférieur à 25 kg/m² et qui ont une activité physique régulière). Une activité physique suffisante est donc nécessaire pour rééquilibrer la balance énergétique chez le sujet obèse.

L'insuffisance d'apport en fruits et légumes favorise le stress oxydatif
La surproduction d'ERDONs dans l'obésité est en partie due à un manque d'apport en antioxydants phytochimiques (composés d'origine végétale comprenant entre autres les caroténoïdes, les flavonoïdes et les isoflavones) et en antioxydants vitaminiques (présents dans les fruits et légumes) (Pincemail et al. 2011). A la Réunion, l'enquête RECONSAL rapporte que moins de la moitié des sujets consomment des légumes tous les jours et seulement 36% des fruits. L'alimentation des personnes interrogées et en particulier des femmes est surtout carencée en minéraux, en vitamines et en fibres.

De plus, l'analyse multivariée descriptive montre que les personnes présentant une obésité abdominale sont plutôt des femmes dont l'apport en lipides correspond à 37% des apports énergétiques totaux journaliers. Les enquêtes alimentaires réalisées chez les sujets obèses (Maskarinec et al. 2000, Canoy et al. 2005, Lairon et al. 2005) montrent que les sujets obèses consomment moins d'antioxydants vitaminiques et d'aliments riches en antioxydants phytochimiques (fruits, végétaux, grains complets, légumes, vin, huile d'olive, grain, noix…) par rapport aux sujets non-obèses. Par exemple, chez des femmes obèses, Moor De Burgos et al. (1992) relève des concentrations en rétinol, vitamine C, vitamine E et β-carotène de 15 à 37 % plus basses comparées à leurs homologues non obèses. Cette déficience d'apport en antioxydants peut initier des désordres métaboliques.
En effet, chez des enfants obèses la diminution d'apport en antioxydants alimentaires (vitamine C, vitamine E et β-carotène) est associée à une élévation de la concentration en leptine et des marqueurs de l'inflammation (CRP, IL-6 et TNF-α) (Aeberli et coll. 2006). Un apport suffisant en antioxydants est donc nécessaire pour rééquilibrer la balance pro / anti-oxydants chez le sujet obèse.
En résumé, la sédentarité et les déséquilibres dans l'apport alimentaire favorisent le développement de l'obésité. L'excédent de substrats énergétiques de type glucose et lipide qui en résulte provoque une hypertrophie et une hyperplasie du tissu adipeux avec un stress oxydatif et un état inflammatoire augmenté. Ces phénomènes expliqueraient la relation obésité et pathologies majeures comme le diabète de type 2 et les maladies cardiovasculaires. Il est donc impératif de proposer des modalités de prévention basées sur une baisse de la masse grasse et une réduction du stress oxydatif et de l'état inflammatoire, par une augmentation de l'activité physique et de la consommation de fruits et légumes. Cela constitue la première étape de la prise en charge des sujets à risque (Haute Autorité de la Santé, Association Américaine pour le Diabète 2006).

2.1.2. SUR LES PROCEDURES DE REFERENCE ET A L'ETUDE

Selon les données de la littérature sur les stratégies de prévention du DT2 et/ou de traitement de l'obésité, une activité physique modérée régulière associée à une alimentation équilibrée, permet de lutter contre les complications biologiques liées à l'obésité en favorisant la perte de masse grasse (ALFEDIAM 1998, ADA 2002, ACSM/AHA 2007, ACSM 2009, PNNS). De nombreux essais de prévention visant à encourager l'activité physique et rééquilibrer l'alimentation des sujets à risque métabolique élevé vont dans ce sens et rapportent des résultats similaires avec une réduction de 28% à 67% de l'incidence du DT2 (Tuomilehto et al. 2001, Knowler et al. 2002, Kosaka et al. 2005, *The Indian Diabetes Prevention Program 2006*, *The China Da Qing Diabetes Prevention Study 2008*). L'étude chinoise (*Da Qing Diabetes Prevention Study*) est un suivi sur 20 ans de l'incidence du DT2 chez 577 sujets intolérants au glucose. La prévalence du diabète au bout de 6 ans était réduite de 42% dans le groupe exercice+diététique montrant ainsi que «Régime + Exercice retardent le diabète de 14 ans». A la Réunion, l'essai d'intervention, Rédia-prev1 (Favier et al. 2005) est un programme de prévention du DT2 basé sur des modifications du comportement alimentaire et une augmentation de l'activité physique (contrôlée mais non individualisée), réalisé en 2002-2003 dans deux quartiers de Saint Pierre. A 15 mois, le poids moyen a diminué de 1,2 kg et le pourcentage de masse grasse de 1,8%. Ces résultats sont plus marqués dans le sous groupe « très bons participants » avec une diminution de 5,1 kg et de 3,8% respectivement. Cet essai d'intervention montre la faisabilité d'un programme de perte de poids dans une population vulnérable, basé sur les modifications du comportement alimentaire et de l'activité physique. Un suivi à 7 ans des participants, pour évaluer la persistance de l'alimentation équilibrée et des activités physiques régulières, est en cours actuellement au CIC-EC (Rédia-prev1 cohorte).

Alimentation, Recommandations sur la consommation de fruits et légumes
Les recommandations du PNNS, dans le cadre de la prévention et du soin, sont de consommer 5 fruits ou légumes verts par jour, soit l'équivalent de 400 grammes (OMS, 2002). Ces recommandations reposent essentiellement sur des hypothèses physiopathologiques et des données transversales ou longitudinales montrant un excès de risque chez les personnes carencées en fruits et légumes (SUVIMAX, Hercberg et al. 2004), et à l'inverse un effet protecteur de cette consommation : apports de fibres, vitamines, oligo-éléments ; ratio satiété / apport calorique très en faveur des fruits et légumes, particulièrement intéressant lorsqu'on pratique une activité physique régulière qui peut ouvrir l'appétit et augmenter la prise alimentaire.

Cependant la consommation de fruits et légumes dans la population, particulièrement chez les personnes à risque de diabète ou de maladies cardio-vasculaires qui sont souvent les plus vulnérables sur le plan social, se heurte aux difficultés financières, aux pressions commerciales et aux habitudes alimentaires.
C'est pourquoi il est nécessaire, si l'on veut étudier l'observance des recommandations du PNNS et l'effet de la consommation de fruits et légumes sur les risques métaboliques et cardiovasculaires, en particulier sur le statut oxydatif et inflammatoires chez les sujets en surpoids ou obèses, de mettre en place un dispositif de fourniture de denrées.
Par ailleurs, le recueil de la consommation des fruits et légumes par les enquêtes alimentaires rencontre les limites qui sont celles des données déclaratives.
Aussi est-il préférable, dans un essai d'intervention basé sur la consommation de fruits et légumes, d'avoir un moyen biologique de contrôle de cette consommation.

Activité physique, Recommandations de Bonnes Pratiques médicales et Recommandations Internationales
Aujourd'hui il n'y a pas de consensus sur l'activité physique en prévention et en soin chez l'adulte obèse.
Cela va des Recommandations de Bonnes Pratiques médicales qui classiquement conseillent « au minimum 30 minutes d'activité physique modérée par jour », sans précisions sur l'intensité et non individualisées, aux recommandations plus élaborées qui tiennent compte des paramètres maximaux des épreuves d'effort (ALFEDIAM 1998, PNNS, ADA 2002, ACSM/AHA 2007, ACSM 2009). L'intensité prescrite est alors exprimée en pourcentage de la consommation maximale d'oxygène (VO_{2max}) ou en pourcentage de la fréquence cardiaque maximale (FC_{max}) mesurées lors d'une épreuve d'effort.

Plusieurs études ont montré l'efficacité, sur la perte de masse grasse, d'un programme de réentraînement à l'effort (RAE) à haute intensité (>60% du VO_{2max}) ou en « interval-training » (alternance entre des phases courtes à haute intensité et des phases d'intensité plus légère) (Tremblay et al. 1994, Irving et al. 2008, Boutcher S. 2011). D'autres, au contraire, privilégient des exercices à faible intensité (≈ 40% du VO_{2max}) (Van-Aggel et al. 2001, Lazzer et al. 2011, Brun et al. 2011). L'intensité optimale de l'exercice physique sur la perte de masse grasse semble donc très controversée (Ballor et al. 1990, Grediagin et al. 1995, Slentz et al. 2004, Nicklas et al. 2009).
Mis à part les résultats du RAE sur le plan physiologique chez le sujet obèse, peu de données existent sur la faisabilité, l'observance, ou encore la tolérance (à moyen et long terme) des séances d'exercice physique suivant l'intensité du programme. Perri et al. (2002) rapportent par exemple une diminution de l'adhérence des sujets lorsque l'intensité augmente mais plus récemment, Irving et al. (2008) ne rapportent aucune différence entre les deux groupes étudiés. On peut s'interroger aussi sur les effets secondaires de l'activité physique, suivant l'intensité, en termes de douleurs articulaires ou musculaires par exemple, et les conséquences sur l'observance à moyen ou long terme. Enfin, si tant est que l'exercice physique à haute intensité est efficace à court terme sur la perte de masse grasse, est-il le plus adapté chez le sujet obèse, sédentaire, pour débuter un programme de réentraînement à l'effort ?

Les limites des recommandations sont qu'elles recouvrent une large gamme d'intensité d'exercice (entre 40% et 60 % de la consommation maximale d'oxygène), et surtout qu'elles ne sont pas individualisées.
En effet, suivant les individus, un même programme d'entraînement peut se traduire par des adaptations physiologiques différentes (Scharhag-Rosenberger et al. 2010). Le projet HERITAGE a montré que les adaptations métaboliques à l'effort sont déterminées par des facteurs individuels qui expliqueraient les différences d'évolution des risques de diabète et des maladies cardiovasculaires, pour un entraînement au même pourcentage de VO_{2max} (Bouchard et al. 2001, Skinner et al. 2003).
Ainsi l'amélioration de l'efficacité de l'activité physique en prévention et en soin reposerait sur la prise en compte des caractéristiques individuelles, et l'on devrait en conséquence s'orienter vers une **prescription individualisée** de l'activité physique**.**

Dans cet esprit, certains auteurs recommandent d'utiliser des paramètres sous-maximaux et individuels comme les seuils ventilatoires et/ou lactiques dans la prescription de l'exercice physique (Mayer et al. 2005, Hofmann et al. 2010), comme c'est le cas dans certaines pathologies chroniques, maladies cardiovasculaires et maladies respiratoires (Tabet et al. 2006, Van Helvoort et al. 2011).

De la même manière chez les personnes obèses, Salvadego et al. (2010) recommandent que l'évaluation et la prescription de l'exercice physique tiennent compte de la réponse métabolique à l'effort, représentée par les cinétiques d'adaptation des différents paramètres d'une épreuve d'effort, et non en fonction du VO_{2max} ou de la FC_{max}.

Le LIPOXmax

Ces dernières années le groupe de Jacques Mercier (CERAMM-CHU Lapeyronie à Montpellier) a montré l'intérêt d'un programme de réentraînement à l'effort individualisé au LIPOXmax chez des sujets obèses et/ou diabétiques. Le LIPOXmax correspond au débit maximal d'oxydation des lipides. Au cours d'un exercice physique, l'oxydation des glucides augmente lorsque l'intensité de l'effort augmente, tandis que l'oxydation des lipides augmente jusqu'à une intensité appelée LIPOXmax puis décroît. Le LIPOXmax est mesuré par calorimétrie indirecte lors d'une épreuve d'effort. Il s'exprime généralement en $mg.min^{-1}$. Le LIPOXmax diffère d'un individu à l'autre, et sa mesure permet de déterminer, chez un individu donné, l'intensité optimale d'effort, pour une consommation maximale de lipides pendant l'exercice.
Ainsi, la mesure par calorimétrie indirecte permet de prescrire un programme de RAE à la fois individualisé et optimisé pour l'oxydation des lipides et la baisse de la masse grasse.

Enfin, en proposant une activité d'intensité légère à modérée, le réentraînement au LIPOXmax est susceptible d'une bonne faisabilité en population, avec une observance à long terme et peu ou pas d'effets secondaires.
Plusieurs études ont déjà mis en évidence l'efficacité d'un réentraînement à l'effort ciblé au LIPOXmax sur la perte de masse grasse, la sensibilité à l'insuline et le métabolisme musculaire chez des patients obèses ou diabétiques (Brandou et al. 2003, Dumortier et al. 2003, Brun et al. 2007-2011, Bordenave et al. 2008).
Les études incluant un entraînement individualisé au LIPOXmax combiné à une alimentation équilibrée montrent une diminution de la masse grasse de 5 à 12 kg en moyenne et une diminution du tour de taille de 7 à 12 cm en moyenne (Brandou et al. 2005, Elloumi et al. 2009, Ben Ounis et al. 2008-2009, Romain et al. 2010). Ces études ont été principalement réalisées chez des adolescents obèses.

Cependant aucune étude n'a encore comparé le réentraînement individualisé au LIPOXmax au réentraînement standardisé selon un pourcentage de VO_{2max}, ou selon les RBP chez l'adulte obèse.

2.2. JUSTIFICATION DES CHOIX METHODOLOGIQUES

Les prévalences élevées du DT2 et de l'obésité chez les femmes justifient de mettre en place un essai d'intervention les concernant ; dans notre expérience à la Réunion, l'enquête Rédia, l'essai Rédia-prev1, les femmes participent plus volontiers que les hommes à la recherche, et ont une meilleure observance ; de plus, dans l'optique de la valorisation des résultats de la recherche, les femmes jouent un rôle prépondérant dans la diffusion des messages de santé ; enfin, dans un essai d'intervention sur l'obésité chez l'adulte, basé sur l'activité physique, il est préférable d'inclure des patientes âgées de 20 à 40 ans, stables sur le plan anthropométrique (pas de variation du poids de plus de 5 kg dans les 6 mois précédant l'étude).
L'IMC est l'indice le plus couramment utilisé pour la mesure de la corpulence en population. Nous l'utiliserons pour la sélection initiale des participantes. Les seuils d'obésité et de surpoids étant classiquement fixés à 30 kg/m² et 25 kg/m² respectivement, nous avons choisi un seuil à 27 kg/m² afin d'avoir une probabilité plus forte d'inclure des femmes ayant un excès de masse grasse et présentant un risque métabolique élevé (Gomez et al. 2011). Dans l'enquête Rédia 2 réalisée en 2009 à la Réunion, 88 % des femmes entre 23 et 39 ans ayant un IMC > à 27 kg/m² présentaient une obésité abdominale (tour de taille > 88 cm, données non publiées).

La masse grasse abdominale est évaluée par la mesure du tour de taille (TT) et du tour de hanches (TH) et l'établissement du rapport TT/TH. Ce sont des mesures qui ont montré des liens très forts entre masse grasse viscérale et risque de DT2 ou de maladie cardio-vasculaire. Cependant des variations inter et intra observateur existent et ces mesures ne sont pas suffisamment précises pour évaluer les variations au cours du suivi.

De même pour l'IMC. S'il est utile pour l'inclusion et la randomisation des patients, il n'est pas suffisamment précis pour mesurer l'évolution de la masse grasse au cours du suivi, le poids d'une personne étant la résultante de sa masse grasse et de sa masse maigre.
A l'issue de l'intervention, s'il s'avérait que l'IMC est resté stable, cela ne signifierait pas forcément qu'il n'y a pas eu de diminution de la masse grasse, mais cela pourrait refléter une augmentation de la masse musculaire concomitante d'une baisse de la masse grasse.
Aussi faut-il utiliser une technique permettant la mesure spécifique de la masse grasse. Les balances à impédancemétrie, très faciles d'utilisation, permettent de calculer le % de masse grasse par une simple pesée. Nous avons prévu d'utiliser une telle balance de façon à disposer de différents types de mesures de la masse grasse. Cependant cette technique est dépendante du niveau d'hydratation du sujet et de l'heure de l'examen, ce qui diminue sa précision et sa reproductibilité.
C'est pourquoi nous avons choisi d'utiliser l'absorptiométrie biphotonique pour mesurer l'évolution de la masse grasse entre l'inclusion et la fin de l'intervention (M_0 et M_5).
Il s'agit d'une méthode de mesure de la composition corporelle (masse minérale, masse grasse et masse maigre) par rayons X, très faiblement irradiante, dont les performances en terme d'exactitude et de reproductibilité sont excellentes (Coefficient de Variation<1).
Concernant le contrôle de la consommation de fruits et légumes, le recueil de cette consommation par les enquêtes alimentaires rencontre les limites qui sont celles des données déclaratives. Aussi, est-il préférable, dans un essai d'intervention basé sur la consommation de fruits et légumes, d'avoir un moyen biologique de contrôle de cette consommation. Des analyses de sang (à M_0, M_3 et M_5) et des dosages inopinés de marqueurs urinaires permettront de contrôler cette consommation.

2.3. PROBLEMATIQUE

- il n'y a pas de consensus en matière d'activité physique pour la prise en charge du surpoids et de l'obésité dans le cadre de la prévention du DT2.
- les hautes intensités d'exercice ont montré leur efficacité sur la perte de poids, mais sans évaluation de la faisabilité en population, l'observance, les effets secondaires, la qualité de vie.
- les effets de l'activité physique sont liés au métabolisme à l'effort de chaque individu. Or les RBP ou les recommandations internationales ne s'appuient pas sur des données individuelles.
- ces recommandations ne ciblent pas une intensité d'effort prenant en compte les substrats et permettant l'utilisation préférentielle des graisses.
- le LIPOXmax combine : individualisation de la prescription d'activité physique ; optimisation de l'utilisation des substrats avec oxydation maximale des lipides ; intensité d'effort légère à modérée favorisant la faisabilité en population, l'observance à long terme et la qualité de vie.
- l'efficacité du réentraînement au LIPOXmax n'a pas été comparée, en population, chez des adultes en surpoids ou obèses, aux réentraînement selon un pourcentage de VO_{2max} ou selon les RBP.
- il existe une relation inverse entre la consommation de fruits et légumes et les risques métaboliques et cardiovasculaires et particulièrement le statut oxydatif et inflammatoire.
- les recommandations de consommer des fruits et légumes se heurtent à des obstacles divers dans la population, d'ordre financier et comportemental, et que l'évaluation de la consommation est rendue imprécise par les limites des données déclaratives.
- il n'y a pas, à notre connaissance, d'essai d'intervention où fruits et légumes sont fournis gratuitement, et leur consommation contrôlée par dosages sanguins et urinaires.

2.4. HYPOTHESES DE LA RECHERCHE ET RESULTATS ATTENDUS

Les hypothèses formulées sont :

a) à dépense énergétique égale, l'activité physique régulière et individualisée au LIPOXmax est plus efficace sur la perte de masse grasse (MG) qu'une activité physique régulière standardisée ou selon les RBP.
b) l'alimentation équilibrée en particulier, supplémentée en fruits et légumes, associée à l'activité physique régulière contribue à la perte de masse grasse et a un effet antioxydant et anti-inflammatoire chez l'adulte en surpoids ou obèse.

2.5. RAPPORT BENEFICE / RISQUE

Le risque lié à l'absorptiométrie est minime. L'absorptiométrie biphotonique utilise les rayons-X, comme la radiographie mais l'irradiation est très faible : 1 à 5 µSv (micro Sievert). Par comparaison, une radiographie des poumons représente une irradiation de 50 µSv. Les risques des prélèvements sanguins sont ceux de la piqûre au pli du coude.

Les bénéfices individuels sont multiples :
-examens biologiques et cliniques permettant de connaître son niveau de risque (statut glycémique, lipidique, inflammatoire, oxydatif ; masse grasse, IMC), connaissance de ses « performances » et aptitudes (épreuve d'effort maximale, LIPOXmax).
-accompagnement pour une pratique d'un réentraînement à l'effort.
-supplémentation gratuite en fruits et légumes pendant 5 mois.
-perte de masse grasse et amélioration des paramètres biologiques et cliniques.
-amélioration de la qualité de vie.
Les risques prévisibles en cours d'étude sont des douleurs articulaires ou musculaires passagères. Les contraintes liées à la recherche sont celles de la pratique régulière d'une activité physique.
Le rapport bénéfice / risque est nettement en faveur du bénéfice et permet de proposer ce protocole à l'étude.

2.6. RETOMBEES ATTENDUES

Les retombées attendues par cette recherche sont :
-la mise en place d'un programme de prévention pérenne visant à favoriser la consommation de fruits et légumes et une activité physique régulière individualisée, prescrite et contrôlée.
-la démonstration de l'intérêt et de la faisabilité de la prescription individualisée d'une activité physique.
Ce projet de recherche-action insiste, notamment au cours des 2 derniers mois, sur la transmission de connaissances et compétences relatives à la gestion de son activité physique (utilisation des cardiofréquencemètres, principes d'échauffement, d'hydratation…). De sorte que les sujets participant à l'étude soient autonomes en fin d'étude (M_5) et se fassent les relais d'information sur l'activité physique adaptée, dans leur entourage. Nous envisageons une diffusion au niveau régional des résultats de l'étude et du programme d'activité physique qui se montrera le plus efficace : médecins, institutions de santé publique, associations de lutte contre l'obésité, le diabète et les maladies cardio-vasculaires, réseaux de prévention …
-la démonstration de l'intérêt et de la possibilité de supplémenter en fruits et légumes l'alimentation des réunionnais, et la facilitation de l'observance de cette consommation lorsque la barrière financière tombe (fruits et légumes fournis gratuitement pendant l'essai). Les retombées sont pour la santé mais également sur le plan de l'économie locale : intérêt de développer l'agriculture vivrière, les circuits courts, les jardins familiaux, l'information sur les fruits et légumes.
Perspectives : Les sujets pourront éventuellement être revus à un an, dans le cadre d'un nouveau projet, pour évaluer la persistance de l'activité physique et de l'alimentation équilibrée, et l'évolution des données biologiques, anthropométriques et du métabolisme à l'effort, à distance de l'intervention.

3. OBJECTIFS DE LA RECHERCHE

3.1. OBJECTIF PRINCIPAL

Il s'agit d'évaluer l'efficacité à 5 mois de trois programmes de réentraînement à l'effort associés à une alimentation équilibrée supplémentée en fruits et légumes, sur la masse grasse chez des femmes de 20-40 ans en surpoids ou obèses.

3.2. OBJECTIFS SECONDAIRES

Il s'agit également d'étudier les effets sur le stress-oxydatif et le statut inflammatoire, comparer l'efficacité des 3 programmes de réentraînement sur les modifications de la composition corporelle, la sensibilité à l'insuline, l'utilisation des lipides à l'effort, la qualité de vie, décrire la faisabilité en population et l'observance.

4. CONCEPTION DE LA RECHERCHE

4.1. SCHEMA DE LA RECHERCHE

- essai d'intervention
- de supériorité
- sans insu
- randomisé en trois groupes parallèles : bras 1 intensité d'effort LIPOXmax, bras 2 intensité d'effort à 60% du VO_{2max}, bras 3 intensité d'effort selon les Recommandations des Bonnes Pratiques, avec alimentation équilibrée supplémentée en fruits et légumes dans les trois bras.
- ratio (1 : 1 : 1) : 126 patientes au total, 42 patientes par groupe
- les personnes des bras 1 et 2 pratiqueront l'activité physique en salle sous le contrôle d'un éducateur sportif. Les personnes du bras 3 pratiqueront « en autonomie » sur leur lieu de vie, sur un parcours de santé ou autre lieu de leur choix.
- monocentrique
- régionale

4.2. METHODES POUR LA RANDOMISATION

La randomisation par bloc est effectuée après stratification sur la classe d'IMC en inclusion :

Strate 1 : $27{,}0 \leq$ IMC $< 30{,}0$ kg/m²
Strate 2 : IMC $\geq 30{,}0$ kg/m²

Les effectifs des 3 groupes sont équilibrés avec un ratio 1:1:1. Les 2 listes de randomisation sont établies par le statisticien du CIC-EC avant le début de la recherche. Un document décrivant la procédure de randomisation (module ralloc de Stata®) est conservé de manière confidentielle au sein du CIC-EC.

5. CRITERES D'ÉLIGIBILITE

Les patientes éligibles pour l'étude « LIPOXmax- Réunion » devront répondre aux critères d'inclusion et de non-inclusion suivants :

5.1. CRITERES D'INCLUSION

- Femmes
- Agées de 20 à 40 ans
- $27 \leq$ IMC ≤ 40 kg/m²
- Glycémie < 1,26g/l
- HbA1c < 6,5%
- Considérées comme cliniquement stables
- Aptes à un programme de réentraînement à l'effort
- Sédentaires (moins de 2 heures d'activités physiques par semaine)
- Patientes ayant signé un consentement de participation à l'étude
- Sans activité professionnelle
- Affiliées à un régime de Sécurité Sociale

5.2. CRITERES DE NON INCLUSION

- HTA sévère : PAS ≥180 mmHg et/ou PAD ≥110 mmHg
- Patiente hypertendue (≥140/90 mmHg) non contrôlée par un traitement médical
- Patiente hypertendue dont le traitement médical comprend des bétabloquants ou des inhibiteurs calciques
- Contre-indication absolue et relative à l'épreuve d'effort (ACC/AHA, 2002) et/ou à l'entraînement physique
- Incapacité de réaliser l'épreuve d'effort maximale et/ou l'épreuve d'effort métabolique
- Pathologie cardiovasculaire et/ou respiratoire non équilibrée révélée par l'épreuve d'effort
- Port de stimulateur ou de défibrillateur cardiaque
- Troubles du rythme ventriculaire sévères non stabilisés sous traitement
- Evènements cardio-vasculaires récents :
  - Décompensation cardiaque ou traitement par drogues inotropes positives depuis moins de 10 jours
  - Angioplastie datant de moins de 10 jours
  - Chirurgie cardiaque datant de moins de 3 mois
  - Maladie valvulaire nécessitant une correction chirurgicale
  - Myopéricardite en évolution
- Myopathie connue et documentée
- Affection cancéreuse
- Maladie aiguë et chronique inflammatoire
- Insuffisance rénale chronique
- Patiente opérée du tube digestif
- Pathologie associée évolutive entraînant une altération importante de l'état général
- Patiente sous corticoïdes, hormones thyroïdiennes, antidépresseurs ou neuroleptiques
- Grossesse en cours ou désir de grossesse avant la visite de fin d'étude
- Déficit mental empêchant la compréhension du consentement éclairé et des protocoles
- Participation à un autre protocole de recherche
- Suivi d'une régime amaigrissant et/ou participation préalable à un programme de réentraînement dans le mois précédent

5.3. MODALITES DE RECRUTEMENT

Le recrutement est prévu via le Service d'endocrinologie - maladies métaboliques – diabétologie du Groupe Hospitalier Sud Réunion et par une annonce dans la presse (cf. annexe 3). Un premier filtre sera réalisé par les médecins du service (vérification de l'âge, du poids et de la taille (déclarés), absence de régime ou programme d'activité physique récents, absence de pathologie et contre indication à la pratique d'une activité physique, absence d'activité professionnelle, pas de projet de grossesse dans les 6 mois). Aux patientes intéressées de participer à ce projet, il sera donné un rendez-vous le matin à jeun pour la pré inclusion. Une quinzaine de patientes seront vues dans la matinée pour les premières mesures. Puis les personnes passeront l'épreuve d'effort cardio-respiratoire.

6. PROCEDURE DE LA RECHERCHE

Toutes les personnes participeront au départ à un atelier d'éducation nutritionnelle assuré par une diététicienne. Ces ateliers seront réalisés dans la cuisine du service d'endocrinologie – maladies métaboliques – diabétologie du GHSR par petits groupes de 10 personnes maximum. Au travers d'exemples concrets, les participantes recevront des informations sur les catégories d'aliments, sur les principes d'une alimentation équilibrée pauvre en huile, en graisse et en sucre rapide, sur les quantités à utiliser, sur la diversité et le rythme de la prise alimentaire.

Pendant toute la durée de l'étude, toutes recevront une supplémentation de 5 fruits et légumes par jour, distribués deux fois par semaine (les patientes viendront récupérer leur « panier »). La Société Promonet-Promédical mettra gratuitement à notre disposition une armoire réfrigérée pour une durée de 6 mois minimum, afin de conserver les fruits et légumes avant distribution.

6.1. PROCEDURE A L'ETUDE

Groupe 1 : groupe LIPOXmax. Activité physique sur ergomètre pendant 5 mois, 4x1heure par semaine à une intensité individualisée qui correspond au niveau optimal d'utilisation des lipides (LIPOXmax), mesuré lors d'une épreuve d'effort sous maximale avec analyse des échanges gazeux.

6.2. PROCEDURES DE COMPARAISON

Groupe 2 : groupe activité physique standard. Activité physique sur ergomètre pendant 5 mois, 4 fois par semaine à une intensité standardisée qui correspond à 60% du VO_{2max} mesuré par une épreuve d'effort cardiorespiratoire. Concernant la durée des séances, elle devra être ajustée de manière à ce que les groupes 1 et 2 aient la même dépense énergétique totale à chaque séance d'entraînement.
Le programme de réentraînement à l'effort durera 5 mois au total, et sera réalisé dans une salle dédiée, située à l'hôpital de St Pierre, sur ergomètre (vélo) par un éducateur sportif. Toutes les participantes seront équipées d'un cardiofréquencemètre. En cas de fatigue de la patiente ou de réserve médicale ou paramédicale, la séance pourra être allégée voire supprimée.
Groupe 3 : les sujets bénéficieront des recommandations de bonnes pratiques en matière d'activité physique (réaliser au minimum 3h30 min d'exercice par semaine à une intensité dite « modérée »), activité qu'ils effectueront en autonomie sur leur lieu de vie, sur un parcours sportif ou autre lieu de leur choix. Pendant 5 mois, un suivi téléphonique sera mis en place tous les mois et ils tiendront un « cahier de suivi d'activités physiques » où ils devront reporter la nature de l'activité (marche, vélo …), la fréquence des séances, la durée de chaque séance et l'intensité de l'effort en inscrivant leur niveau d'essoufflement suivant l'échelle de Borg et la fréquence cardiaque d'entraînement. Il leur sera demandé de porter un podomètre lors des séances de marche et de noter le nombre de pas par jour effectués. Les données seront recueillies tous les 15 jours. Elles permettront d'estimer la dépense énergétique du groupe 3.

Le tableau suivant résume les caractéristiques des trois groupes de notre étude :

	**Groupe 1**	**Groupe 2**	**Groupe 3**
**Durée du programme**	5 mois sur ergomètre		5 mois
**Durée d'une séance**	1 heure	Ajustée pour la même dépense calorique que G1	Recommandations de bonnes pratiques en activité physique et suivi téléphonique
**Fréquence des séances**	4 séances / semaine	4 séances / semaine	
**Intensité de l'exercice**	individualisée au $LIPOX_{max}$	standardisée à 60% du VO_{2max}	
**Diététique**	Education nutritionnelle + supplémentation en fruits et légumes		

6.3. PRODUITS ALIMENTAIRES (FRUITS ET LEGUMES)

L'observance de la consommation des fruits et légumes sera contrôlée par un questionnaire bimensuel. Sur le plan biologique, la consommation effective sera contrôlée par des marqueurs sanguins, lors des analyses prévues dans le protocole à M_0, M_3 et M_5 et par des marqueurs urinaires lors des dosages inopinés.

Les fruits et légumes seront fournis par la cuisine centrale du GHSR qui distribue quasi exclusivement les mêmes fruits et légumes qu'en métropole : orange, pomme, poire, banane pour les fruits ; tomate, concombre, carotte, choux, citrouille, aubergine, courgette, poivron, épinards, laitue, pour les légumes. La plupart des denrées sont des produits d'importation (métropole, Afrique du Sud, Australie), la production locale étant insuffisante et plus chère. Quelques produits typiques du terroir réunionnais peuvent selon la saison, de façon rare et épisodique, venir compléter ce tableau : ananas, mangue, papaye, letchis, christophine, brèdes…mais il a été démontré que leur activité antioxydante ne diffère pas des fruits et légumes courants. De ce fait, les fruits et légumes qui seront proposés au cours de cette étude ne seront pas spécifiques du lieu, ce qui écarte la question de son intérêt limité au département de La Réunion.

7. <u>CRITERES DE JUGEMENT</u>

7.1. CRITERE DE JUGEMENT PRINCIPAL

L'efficacité des programmes sera jugée sur l'évolution entre M_0 et M_5 de la masse grasse.
Elle est mesurée principalement par absorptiométrie biphotonique (ostéodensitomètre Lunar) ; et par impédancemétrie (balance Tanita), IMC, tour de taille et tour de hanches. Les mesures sont pratiquées par le médecin investigateur, qui recueille les données.
La masse grasse par absorptiométrie biphotonique est exprimée en kilogrammes (en % pour l'impédancemétrie, en kg/m² pour l'IMC, en cm pour TT et TH)
L'évolution de la masse grasse sera évaluée par des mesures à l'inclusion (M_0) par :

- Absorptiométrie, impédancemétrie, IMC, tour de taille et tour de hanches.

Au bout de 3 mois (M_3) par :

- Impédancemétrie, IMC, tour de taille et tour de hanches.

En fin d'étude, au bout de 5 mois (M_5) par :

- Absorptiométrie, impédancemétrie, IMC, tour de taille et tour de hanches.

7.2. CRITERES DE JUGEMENT SECONDAIRES

L'efficacité de l'intervention sera également jugée sur l'évolution entre M_0 et M_5 :

- du statut inflammatoire et antioxydant et des autres paramètres biologiques (CRP, albumine, TNFα, IL-6, leptine, adiponectine, test Folin et test DPPH, glycémie, insulinémie, % d'HbA1c, cholestérol total, HDL, LDL, triglycérides).
- de la composition corporelle (poids, IMC, tour de taille, tour de hanche, masse musculaire).
- de l'utilisation des lipides à l'effort : LIPOXmax mesuré par calorimétrie indirecte.
- de la qualité de vie.
- du recueil des effets secondaires (douleurs, fatigue…).
- de la faisabilité : évaluation de l'adhésion et de l'observance.

8. **<u>DEROULEMENT DE LA RECHERCHE</u>**

8.1. CALENDRIER DE LA RECHERCHE

La durée totale de l'étude est estimée à environ deux ans.

- Durée de la période d'inclusion : 2 mois
- Durée de participation de chaque patient : 5 mois
- Saisie des données, analyse et valorisation : 17 mois
- Durée totale de la recherche : 24 mois
- Début estimé de l'étude : 3ème trimestre 2011 et fin estimée : 2ème trimestre 2013

8.2. TABLEAU RECAPITULATIF DU SUIVI PATIENT

<table>
<tr><th></th><th>Pré-inclusion J-3 à J-21</th><th>Inclusion J_0</th><th>Visite J_1</th><th>Visite J_2</th><th>Visite J_3</th><th>Visite M_3</th><th>Visite M_5</th></tr>
<tr><td>Consentement éclairé</td><td>✓</td><td></td><td></td><td></td><td></td><td></td><td></td></tr>
<tr><td>Examen clinique et anthropométrique[1]</td><td>✓</td><td></td><td></td><td></td><td></td><td>✓</td><td>✓</td></tr>
<tr><td>βHCG</td><td>✓</td><td></td><td></td><td></td><td></td><td>✓</td><td>✓</td></tr>
<tr><td>Epreuve d'effort cardiorespiratoire maximale</td><td>✓</td><td></td><td></td><td></td><td></td><td></td><td></td></tr>
<tr><td>Randomisation</td><td></td><td>✓</td><td></td><td></td><td></td><td></td><td></td></tr>
<tr><td>Impédancemétrie</td><td></td><td>✓</td><td></td><td></td><td></td><td>✓</td><td>✓</td></tr>
<tr><td>Absorptiométrie</td><td></td><td>✓</td><td></td><td></td><td></td><td></td><td>✓</td></tr>
<tr><td>Bilan biologique[2]</td><td></td><td>✓</td><td></td><td></td><td></td><td>✓</td><td>✓</td></tr>
<tr><td>Plasmathèque[3]</td><td></td><td>✓</td><td></td><td></td><td></td><td>✓</td><td>✓</td></tr>
<tr><td>Bilan urinaire[4]</td><td></td><td>✓</td><td></td><td></td><td></td><td>✓</td><td>✓</td></tr>
<tr><td>Epreuve d'effort métabolique</td><td></td><td></td><td>✓</td><td></td><td></td><td>✓</td><td>✓</td></tr>
<tr><td>Questionnaire sociodémographique, alimentaire, activité physique</td><td></td><td>✓</td><td></td><td></td><td></td><td></td><td></td></tr>
<tr><td>Questionnaire de qualité de vie</td><td></td><td>✓</td><td></td><td></td><td></td><td>✓</td><td>✓</td></tr>
<tr><td>Education nutritionnelle</td><td></td><td></td><td></td><td>✓</td><td></td><td></td><td></td></tr>
<tr><td>Séances d'activité physique + Supplémentation de 5 fruits et légumes par jour[5]</td><td></td><td></td><td></td><td></td><td colspan="3">✓</td></tr>
<tr><td>Questionnaire sur les effets secondaires de l'activité physique et la consommation de F&L (bimensuel)</td><td></td><td></td><td></td><td></td><td colspan="3">✓</td></tr>
</table>

[1]Examen clinique et anthropométrique : antécédents médicaux et personnels, glycémie capillaire à jeun, HbA1c, TA, taille, poids, tour de taille et de hanches.
[2]Bilan biologique : glycémie veineuse, insulinémie, cholestérol total, HDL/LDL-Cholestérol, TG, CRP, albumine, créatinine,
[3]Plasmathèque : statut antioxydant, test Folin et DPPH, marqueurs inflammatoire, TNF-alpha, IL-6, marqueurs de consommation de F et L.
[4]Bilan urinaire : protéinurie, albuminurie, acides caféique, chlorogénique, férulique et gallique.
[5]A partir de J_3 et jusqu'à la visite de fin d'étude (M_5) les participantes des groupes 1 et 2 viendront 4 fois par semaine réaliser leurs séances d'activité physique dans une salle de l'hôpital. Les participantes du groupe 3 réaliseront leurs activités physiques chez elles.
Toutes les participantes viendront 2 fois par semaines chercher leurs rations de fruits et légumes.

L'épreuve d'effort cardiorespiratoire maximale (M_0 uniquement) sera réalisée par un cardiologue dans une salle dédiée sur cyclo-ergomètre. Après une phase de repos de 3 minutes assis sur le vélo et une phase d'échauffement pendant 2 minutes à 20% de la puissance maximale théorique, la charge de travail sera progressivement augmentée de 15 watts toutes les minutes jusqu'aux capacités maximales de la patiente selon les recommandations classiques de la littérature (ACC/AHA, 2002). La fréquence de pédalage imposée sera comprise entre 60 et 70 tours par minute. La durée totale de l'exercice devra être comprise entre 8 et 15 minutes. Une phase de récupération active (2 minutes à 30 watts) puis passive (4 minutes sans charge assis sur le vélo) terminera l'épreuve d'effort. Au cours de cette épreuve, les patientes seront surveillées en continu par un électrocardiogramme 12-dérivations et par la mesure de la pression artérielle sur un bras détendu toutes les 2 minutes. Les échanges gazeux pulmonaires et les réponses ventilatoires seront mesurés de façon continue par un système de mesure « en cycle à cycle » automatisé. La saturation artérielle en O_2 sera enregistrée tout au long de l'épreuve d'effort maximale par un saturomètre.
Les analyses biologiques des échantillons sanguins seront collectées par prélèvement veineux au pli du coude. 13 mL au total seront nécessaires pour chaque prélèvement. Les échantillons seront prélevés dans le service d'endocrinologie – maladie métabolique – diabétologie du GHSR. Les analyses « de routine » seront effectuées au Laboratoire de Biochimie du GHSR pour l'évaluation de l'insulinémie, glycémie, hémoglobine glycosylée ou HbA1c, lipidémie (cholestérol, HDL/LDL, triglycérides, lipides totaux), albuminémie et CRP.

La glycémie sera réalisée sur un tube contenant du fluorure; l'insuline sur un tube sans additif et le bilan lipidique, l'albuminémie et la CRP sur un tube avec activateur de coagulation et gel séparateur. 1mL pour chaque tube sera suffisant donc 3 mL au total seront nécessaires pour ces analyses.
Les analyses « plus sophistiquées » du statut oxydatif et inflammatoire seront réalisées par le Groupe d'Etude sur l'Inflammation Chronique et l'Obésité (GEICO) de l'Université de la Réunion, sous la responsabilité du Dr-HDR Marie-Paule Gonthier basée au Département Génie Biologique de l'IUT de Saint-Pierre qui se situe à proximité du GHSR (200 mètres). 2 tubes héparinés de 5 mL chacun seront nécessaires pour ces analyses. Les échantillons collectés seront tous transférés, via la navette du GHSR, sous conditionnement à température contrôlée (4°C) du GHSR au Département Génie Biologique de l'IUT. Dès l'arrivée au laboratoire, chaque échantillon sanguin sera immédiatement pris en charge afin d'isoler le plasma qui sera conservé à -80°C jusqu'à analyse et validation de toutes les données expérimentales pour la période de cinq mois d'étude.
Tous les échantillons sanguins seront détruits à la fin de l'étude.
En termes de manipulations, dès réception au laboratoire, le sang collecté sera en effet immédiatement centrifugé pour récupérer le plasma, qui sera acidifié avec 10 mM d'acide acétique afin d'éviter les pertes en antioxydants qui surviennent lors de l'alcalinisation du plasma. Les marqueurs moléculaires qui seront explorés pour évaluer l'état inflammatoire des sujets sont : le Tumor Necrosis Factor-α (TNF- α), l'Interleukine-6 (IL-6), la leptine et l'adiponectine. Ces analyses seront réalisées grâce à l'utilisation de kits immunologiques de type ELISA et d'un lecteur de microplaques de type FLUOSTAR. Le statut antioxydant sera quant à lui exploré au travers de la mesure de la capacité antioxydante totale du plasma par le test colorimétrique de Folin-Ciocalteu et le test anti-radicalaire vis-à-vis du Diphényl-pycril-hydrazil (DPPH).
Afin de suivre la consommation de produits végétaux chez les sujets, une recherche de biomarqueurs d'exposition aux composés phénoliques antioxydants d'origine végétale sera réalisée à M_0, M_3, M_5, grâce à l'analyse du plasma par la technique de Chromatographie Liquide Haute Performance couplée à une détection ultra-violet (HPLC-UV). Les principaux marqueurs moléculaires explorés seront les acides caféique, chlorogénique, férulique et gallique, correspondant à des acides phénoliques abondants dans les fruits et dont les travaux antérieurs de Gonthier et al. (2003, 2005, 2006) ont démontré la biodisponibilité chez l'homme. Des marqueurs moléculaires de la consommation de légumes de type épicatéchine et quercétine ainsi que deux principaux métabolites circulants (l'acide hippurique et l'acide 3-hydroxyphénylpropionique) seront également dosés. En outre des prélèvements d'urine (10 mL) inopinés seront pratiqués pour effectuer des dosages urinaires (protéinurie, albuminurie, acides caféique, chlorogénique, férulique et gallique).
L'épreuve d'effort métabolique (M_0, M_3, M_5) sera réalisée dans une salle dédiée sur ergocyle. Une phase de repos de 3 minutes assis sur le vélo et une phase d'échauffement de 3 minutes à 20% de la puissance maximale théorique (PMT) démarreront l'épreuve, suivies de 4 paliers de 6 minutes à 30, 40, 50, 60% de la PMT. La fréquence de pédalage imposée sera comprise entre 60 et 70 tours par minute. Une phase de récupération active (2 minutes à 20% de la PMT) puis passive (2 minutes sans charge assis sur le vélo) terminera l'épreuve d'effort. Les patientes seront surveillées en continu par un électrocardiogramme 12-dérivations et par la mesure de la pression artérielle toutes les 2 minutes.
Les échanges gazeux pulmonaires et les réponses ventilatoires seront mesurés de façon continue par un système de mesure « en cycle à cycle » automatisé. Durant les deux dernières minutes de chaque palier, les valeurs de VCO_2 et VO_2 seront enregistrées afin de calculer les débits d'oxydation des glucides et des lipides pour la mesure du LIPOXmax et du Point de Croisement des substrats.
L'enquête alimentaire consistera en un questionnaire de fréquence avec quantification par photos.
Le questionnaire d'activité physique (inspiré du questionnaire de Baecke traduit en français par Bigard et al. 1992) a été validé dans plusieurs études réalisées au CIC-EC (Réconsal, Rédia-prev1…). C'est un auto-questionnaire qui comporte 5 échelles : activités physiques liées au travail, aux déplacements, aux taches domestiques, aux activités de loisirs, aux activités sportives.
Le questionnaire bimensuel est un auto questionnaire portant sur la prise effective des fruits et légumes et sur les éventuels effets secondaires de l'activité physique (vécu et tolérance des séances d'exercice physique). Les éventuelles douleurs musculaires et/ou articulaires y seront répertoriées.
Le questionnaire de qualité de vie est un questionnaire générique : Medical Outcome Survey Short Form 36-items (MOS SF-36) (Ware et al. 1993). Il comprend 36 items répartis dans 8 échelles. Les échelles sont comprises entre 0 et 100 ; plus le score est élevé, meilleure est la valeur perçue dans l'échelle concernée. Les échelles sont réparties dans deux domaines : le score global physique (PCS) et le score global psychique

(MCS). Les deux domaines ont été normalisés à la population générale dont la valeur moyenne correspond à un score de 50/100 et un écart type de 10 (Ware et coll. 1993, 1994).

8.3. RECRUTEMENT DES PERSONNES

Les médecins du service d'endocrinologie - maladies métaboliques - diabétologie du GHSR, informent les patientes au sujet de l'étude *LIPOXmax-Réunion* et vérifient les premiers critères d'inclusion/non inclusion tels que : âge, poids et taille, absence de régime ou programme d'activité physique récents, absence de pathologie et contre indication à la pratique d'une activité physique, absence d'activité professionnelle, ou de désir de grossesse pendant la durée de participation de chaque personne.

8.4. VISITE DE PRE-INCLUSION

La visite de pré-inclusion à lieu dans le service d'endocrinologie – maladies métaboliques – diabétologie du GHSR par le médecin investigateur. Elle a lieu entre 3 jours et 3 semaines au plus tard avant la visite d'inclusion. Avant tout examen lié à la recherche, l'investigateur recueille le consentement libre, éclairé et écrit de la patiente (ou de son représentant légal le cas échéant).

RECUEIL DU CONSENTEMENT

Lors de la visite de pré-inclusion, le médecin investigateur informe la patiente et répond à toutes ses questions concernant l'objectif, la nature des contraintes, les risques prévisibles et les bénéfices attendus de la recherche. Il précise également les droits du patient dans le cadre d'une recherche biomédicale et vérifie les critères d'éligibilité. Un exemplaire de la note d'information et du formulaire de consentement est alors remis à la patiente par le médecin investigateur.

Après cette séance d'information, la patiente dispose d'un délai de réflexion. Le médecin investigateur est responsable de l'obtention du consentement éclairé écrit du patient. Le formulaire de consentement doit être signé AVANT LA REALISATION DE TOUT EXAMEN CLINIQUE OU PARACLINIQUE NECESSITE PAR LA RECHERCHE.

Si la patiente donne son accord de participation, cette dernière et l'investigateur inscrivent leurs noms et prénoms en clair, datent et signent le formulaire de consentement.

Les différents exemplaires de la note d'information et du formulaire de consentement sont alors repartis comme suit :

- Un exemplaire de la note d'information et du consentement signé est remis à la patiente.
- L'exemplaire original est conservé par le médecin investigateur (même en cas de déménagement de la patiente pendant la durée de la recherche) dans un lieu sûr inaccessible à des tiers.
- A la fin des inclusions ou au plus tard à la fin de la recherche, un exemplaire de chaque formulaire de consentement est transmis au promoteur ou à son représentant selon des modalités communiquées en temps utile aux investigateurs.

La visite de pré-inclusion comprend :

- un recueil des antécédents médicaux
- un recueil des paramètres glycémique et des mesures anthropométriques : glycémie capillaire, HbA1c, poids, taille, IMC, tour de taille et tour de hanches, tension artérielle
- le dosage du taux de βHCG urinaire
- une épreuve d'effort cardiorespiratoire maximale réalisée par un cardiologue

8.5. VISITE/DEMARCHE DE RANDOMISATION

Après la vérification des critères d'éligibilité, la demande de randomisation est transmise au statisticien du CIC-EC. Le statisticien renvoie à l'investigateur, à la coordinatrice d'enquête et au chargé de projet le bras de randomisation de la patiente.

8.6. VISITE D'INCLUSION

Jour 0 (minimum 3 jours après l'épreuve d'effort cardiorespiratoire maximale) **:**

- Bilan biologique : glycémie veineuse, insulinémie, cholestérol total, HDL-Cholestérol, LDL-Cholestérol, triglycérides, créatinine, CRP, albumine
- Constitution d'une plasmathèque afin de doser : le statut antioxydant (test Folin et DPPH), le TNF-alpha, IL6, adiponectine, leptine, marqueurs de consommation de fruits et légumes
- Bilan urinaire : protéinurie, albuminurie, acides caféique, chlorogénique, férulique et gallique
- Examens para-cliniques à réaliser : absorptiométrie biphotonique, impédancemétrie.
- Questionnaires (données sociodémographiques, enquête alimentaire, activité physique, qualité de vie).

Jour 1 :

- Epreuve d'effort métabolique avec mesure du LIPOXmax.

Jour 2 :

- Atelier d'éducation nutritionnelle.

A partir du Jour 3 et jusqu'à M5:

- Démarrage des séances d'activité physique et de la supplémentation en fruits et légumes.

  Recueil d'un questionnaire sur les effets secondaires de l'activité physique (de façon bimensuelle). L'ensemble des participantes remplira un auto-questionnaire bimensuel sur leur consommation de fruits et légumes effective et le vécu de leur activité physique, et les éventuels effets secondaires. Cet auto-questionnaire sera rempli à l'occasion de la venue des participantes pour récupérer les fruits et légumes (aide par enquêteur pour les personnes illettrées). Les personnes des groupes 1 et 2 signeront à chaque séance d'activité physique une liste d'émargement ; seront recueillis les paramètres suivants : durée exacte de la séance, calories dépensées, puissance, fréquence cardiaque. Le nombre total de séances par participant sera comptabilisé en fin d'essai et cette donnée sera prise en compte dans les analyses statistiques. Les personnes du groupe 3 (activité physique autonome selon les RBP) rempliront un cahier de suivi où seront consignées les modalités de pratique de leurs activités physiques (nature, fréquence, durée, intensité, déroulement). Les données mesurées pour chaque patiente seront colligées dans son cahier d'observation.

8.7. VISITES DE SUIVI M_3

Une visite de suivi est prévue au bout de 3 mois (M_3). Tous les examens d'inclusion seront reprogrammés (sauf l'absorptiométrie biphotonique et l'épreuve d'effort cardiorespiratoire maximale). Elle comprendra donc :

- un recueil des paramètres glycémique et des mesures anthropométriques : glycémie capillaire, HbA1c, poids, taille, IMC, tour de taille et tour de hanches, tension artérielle
- l'impédancemétrie

- bilan biologique : glycémie veineuse, insulinémie, cholestérol total, HDL-Cholestérol, LDL-Cholestérol, triglycérides, créatinine, CRP, albumine,
- bilan urinaire: protéinurie, albuminurie, acides caféique, chlorogénique, férulique et gallique
- le dosage du taux de βHCG urinaire
- Constitution d'une plasmathèque afin de doser : le statut antioxydant (test Folin et DPPH), le TNF-alpha, IL6, adiponectine, leptine, marqueurs de consommation de fruits et légumes
- l'épreuve d'effort métabolique
- le questionnaire de qualité de vie

Cette visite permettra :

- de réactualiser les programmes de réentraînement à l'effort des groupes 1 et 2. Les intensités d'exercices prescrites à M_0 auront vraisemblablement évolué au bout de 3 mois pour ces deux groupes. Ils convient de les réévaluer.
- de suivre l'évolution de tous les critères mesurés en milieu d'étude.

8.8. VISITE DE SUIVI M_5

La dernière visite de suivi (M_5) comporte les mêmes examens que la visite d'inclusion (sauf l'épreuve d'effort cardiorespiratoire maximale et les questionnaires portant sur les données sociodémographiques, l'alimentation et l'activité physique).

Elle comprendra donc :

- un recueil des paramètres glycémique et des mesures anthropométriques : glycémie capillaire, HbA1c, poids, taille, IMC, tour de taille et tour de hanches, tension artérielle
- l'absorptiométrie biphotonique
- l'impédancemétrie
- bilan biologique : glycémie veineuse, insulinémie, cholestérol total, HDL-Cholestérol, LDL-Cholestérol, triglycérides, créatinine, CRP, albumine,
- bilan urinaire: protéinurie, albuminurie, acides caféique, chlorogénique, férulique et gallique
- le dosage du taux de βHCG urinaire
- Constitution d'une plasmathèque afin de doser : le statut antioxydant (test Folin et DPPH), le TNF-alpha, IL6, adiponectine, leptine, marqueurs de consommation de fruits et légumes
- l'épreuve d'effort métabolique
- le questionnaire de qualité de vie

8.9. VISITE DE FIN DE LA RECHERCHE

La fin de la recherche correspond au terme de la participation de la dernière personne qui se prête à la recherche.

8.10. REGLES D'ARRET DE LA RECHERCHE

Les règles d'arrêt temporaire de la participation d'une personne à la recherche ou d'une partie ou de la totalité de la recherche :
1) toute personne est libre d'arrêter temporairement sa participation à la recherche à tout moment

2) toute personne peut demander d'interrompre temporairement sa participation en raison d'effets secondaires de l'activité physique (douleurs articulaires, musculaires…)
3) toute personne peut présenter une indisponibilité temporaire due à des problèmes personnels ou familiaux, une maladie aiguë…
Une personne des groupes 1 et 2 qui n'a pas manqué plus de 4 séances consécutives ou 9 séances en tout sur les 5 mois (soit 10% des séances) peut maintenir sa participation à la recherche si elle le souhaite.

Les règles d'arrêt définitif :
1) toute personne est libre d'arrêter définitivement sa participation à la recherche à tout moment sans avoir à donner de raison
2) toute personne présentant un problème de santé incapacitant lui interdisant la pratique d'une activité physique sera exclue de la recherche sur décision de l'Investigateur Principal qui en informera la coordinatrice, le chargé de projet et le Promoteur.
Il n'est pas prévu de remplacement des personnes arrêtant la recherche.

8.11. INDEMNISATION EVENTUELLE DES SUJETS

Les personnes recevront une indemnité pour les contraintes liées à leur participation. Une indemnisation de 100 euros par mois pendant 5 mois se fera au prorata de leur participation. Si on considère qu'un mois comporte 20 séances en moyenne, 5 euros seront déduit de l'indemnisation pour chaque absence.

8.12. COLLECTION BIOLOGIQUE

Deux tubes de 5 mL chacun, soit 10 mL au total, serviront à la constitution d'une plasmathèque pour l'analyse du statut oxydatif et inflammatoire qui sera réalisée par le Groupe d'Etude sur l'Inflammation Chronique et l'Obésité (GEICO) de l'Université de la Réunion. Les analyses seront effectuées au Département Génie Biologique de l'IUT de Saint-Pierre situé à proximité du GHSR (200 mètres). Les échantillons collectés seront tous transférés, par la navette du GHSR, sous conditionnement à température contrôlée (4°C) du GHSR au Département Génie Biologique de l'IUT. Dès l'arrivée au laboratoire, chaque échantillon sanguin sera immédiatement pris en charge afin d'isoler le plasma qui sera conservé à -80°C jusqu'à analyse et validation de toutes les données expérimentales pour la période de cinq mois d'étude.

A la fin de l'étude, tous les échantillons seront détruits.

9. GESTION DES ÉVÉNEMENTS INDÉSIRABLES ET DES FAITS NOUVEAUX

9.1. DEFINITIONS

Evénement indésirable (article R.1123-39 du code de la santé publique)
Toute manifestation nocive survenant chez une personne qui se prête à une recherche biomédicale, que cette manifestation soit liée ou non à la recherche ou au produit sur lequel porte cette recherche.

Evénement indésirable grave (article R.1123-39 du code de la santé publique et guide ICH E2B)
Tout événement indésirable qui :
- ✓ entraîne la mort,
- ✓ met en danger la vie de la personne qui se prête à la recherche,
- ✓ nécessite une hospitalisation ou la prolongation de l'hospitalisation,
- ✓ provoque une incapacité ou un handicap important(e) ou durable,
- ✓ se traduit par une anomalie ou une malformation congénitale,
- ✓ ou tout événement considéré médicalement grave.

Effet indésirable inattendu (article R.1123-39 du code de la santé publique)
Tout effet indésirable dont la nature, la sévérité ou l'évolution ne concorde pas avec les informations relatives aux actes pratiqués, et méthodes utilisées au cours de la recherche. L'évaluation du caractère inattendu d'un effet indésirable se fait sur la base des informations décrites dans le protocole ou la brochure pour l'investigateur, relatives notamment, le cas échéant, aux actes et méthodes pratiqués au cours de la recherche ou aux produits faisant l'objet de la recherche ou utilisés pour les besoins de la recherche.

Fait nouveau (arrêté du 24 mai 2006)
Nouvelle donnée de sécurité, pouvant conduire à une réévaluation du rapport des bénéfices et des risques de la recherche, ou qui pourrait être suffisante pour envisager des modifications des documents relatifs à la recherche, de la conduite de la recherche ainsi que, le cas échéant, dans l'utilisation du produit.

9.2. DESCRIPTION DES EVENEMENTS INDESIRABLES GRAVES ATTENDUS

Aucun événement indésirable grave n'est attendu dans le cadre du protocole. En effet, les activités physiques proposées seront de faible ou moyenne intensité, ne présentant pas de risque pour les personnes qui les pratiquent. Les critères d'inclusion (absence de maladie grave, diabète, HTA sévère et la sélection des participants par l'épreuve d'effort cardiorespiratoire) font que le risque d'EIG tend vers zéro.
La réalisation de l'épreuve d'effort fait partie de l'évaluation habituelle des patientes obèses étant donné qu'elles peuvent présenter des facteurs de risque cardiovasculaires associés. Ces personnes sont donc classiquement suivies par un cardiologue. Les risques de l'épreuve d'effort rapportés dans la littérature sont faibles (environ 1 décès par arythmie ventriculaire pour 75 000 à 100 000 examens) (Douard et coll., 1987). En accord avec les bonnes pratiques, une information sur l'épreuve d'effort, suivant le modèle proposé par la Société Française de Cardiologie sera donnée à la patiente.

9.3. CONDUITE A TENIR EN CAS D'EVENEMENT INDESIRABLE OU DE FAIT NOUVEAU

L'investigateur doit notifier au promoteur, sans délai à partir du jour où il en a connaissance, tout événement indésirable grave ou tout fait nouveau, s'il survient :
- à partir de la date de signature du consentement,
- pendant toute la durée de suivi du patient prévue par la recherche,
- jusqu'à 2 jours après la fin du suivi du participant prévue par la recherche, lorsqu'il est susceptible d'être dû à la recherche.

TYPE D'EVENEMENT	*MODALITES DE NOTIFICATION*	*DELAI DE NOTIFICATION AU PROMOTEUR*
EI non grave	*Dans le cahier d'observation (questionnaire sur les éventuels effets secondaires liés à l'exercice physique)*	*Pas de notification immédiate*
EIG attendu	*Formulaire de déclaration d'EIG initiale + rapport écrit si nécessaire*	***Notification immédiate au promoteur***
EIG inattendu	*Formulaire de déclaration d'EIG initiale + rapport écrit si nécessaire*	***Notification immédiate au promoteur***
Fait nouveau	*Formulaire de déclaration + rapport écrit si nécessaire*	***Notification immédiate au promoteur***
Grossesse	*Formulaire de déclaration d'une grossesse*	*Dès confirmation de la grossesse*

DRCI du CHR de La Réunion
Tél : 02 62 35 95 25
Fax : 02 62 35 97 21
Courriel : vanessa.basque@chr-reunion.fr

Le promoteur de la recherche transmet les EIG qui lui sont notifiés à l'unité de vigilance de la recherche clinique qui détermine l'imputabilité de l'événement à l'étude et la nécessité d'une déclaration aux autorités compétentes.
Tous ces événements devront être suivis jusqu'à la **complète résolution**. Un complément d'information (fiche de déclaration complémentaire) concernant l'évolution de l'événement, si elle n'est pas mentionnée dans le premier rapport, sera envoyé au promoteur par l'investigateur.
La survenue d'une grossesse dans la période de la recherche, ne constitue pas un EIG. Cependant, une grossesse doit être notifiée selon les mêmes modalités qu'un EIG car elle entraînera l'exclusion de la recherche.

9.4. DECLARATION ET ENREGISTREMENT DES EIG INATTENDUS ET DES FAITS NOUVEAUX

Le promoteur/l'unité de vigilance déclare sans délai les EIG inattendus et les faits nouveaux survenus au cours de la recherche :

- à l'Afssaps
- au Comité de Protection des Personnes compétent. Le comité s'assure, si nécessaire, que les sujets participant à la recherche ont été informés des effets indésirables et qu'ils confirment leur consentement.

10. ASPECTS STATISTIQUES

10.1. CALCUL DE LA TAILLE D'ETUDE

LIPOXmax-Réunion est un essai d'intervention randomisé permettant d'évaluer la baisse de la masse grasse et l'amélioration du statut oxydatif et inflammatoire dans le cadre de la prévention du diabète de type 2 chez les jeunes femmes en surpoids ou obèses, comparant trois groupes parallèles :

Groupe 1 : activité physique individualisée au LIPOXmax + supplémentation en fruits et légumes
Groupe 2 : activité physique à 60% du VO_{2max} + supplémentation en fruits et légumes
Groupe 3 : activité physique selon les RBP + supplémentation en fruits et légumes

La durée de suivi de chaque sujet est de 5 mois à partir de l'inclusion. Il s'agit d'un essai de supériorité.

L'évaluation de l'efficacité repose sur le critère de jugement principal suivant : évolution entre M_0 et M_5 de la masse grasse (kg) mesurée par absorption biphotonique (DXA).

Le bénéfice attendu est une diminution de la masse grasse à 5 mois, d'au moins :

- 1,5 kg entre le groupe 1 et le groupe 2.
- 2,0 kg entre le groupe 2 et le groupe 3.
- 4,0 kg entre le groupe 1 et le groupe 3.

Une différence de 1,5 kg à 5 mois entre les groupes 1 et 2 apparaît pertinente (Mougio et al. 2006, Romain et al. 2010, Brun et al. 2010, Lazzer et al. 2011).

Pour mettre en évidence une différence d'évolution moyenne de 1,5 kg de masse grasse, avec un écart-type de 2,0 kg, un risque de première espèce de 1,67 % (5 % / 3), une puissance de 80 %, il est nécessaire d'inclure en situation bilatérale 38 sujets par groupe.

Dans de telles conditions, il sera possible de mettre significativement en évidence une différence minimum théorique entre groupes de :

- 2,3 % sur l'évolution à 5 mois du pourcentage de masse grasse moyen (écart-type = 3 %).
- 3,8 kg sur l'évolution à 5 mois du poids moyen (écart-type = 5 kg).

En raison d'un taux de données manquantes (abandons, perdus de vue) estimé à 10 %, il est prévu d'inclure 42 sujets par groupe, soit 126 au total.

10.2. METHODES STATISTIQUES EMPLOYEES

Un plan d'analyse détaillé sera défini et fera l'objet d'une validation par le conseil scientifique de l'étude. Les modifications ultérieures seront systématiquement validées par le conseil scientifique.

Les analyses statistiques seront réalisées au CIC-EC de La Réunion, sous SAS® (version 9.2, SAS Institute Inc., Cary, NC, USA).

Proposition de plan d'analyse à faire valider par le conseil scientifique de l'étude :

1) Construction d'un diagramme des flux détaillant le nombre de sujets pré-inclus, le nombre de sujets inclus, le nombre de sujets pré-inclus non inclus dans chacun des trois groupes, le nombre de sujets suivis jusqu'à M_3 et M_5 dans chacun des trois groupes et le nombre de perdus de vue, abandons, décès.

2) Description des caractéristiques initiales des sujets inclus (sur l'ensemble de la population et par groupe) - vérification de la comparabilité initiale des trois groupes (effectif, pourcentage, moyenne ± écart-type ou médiane et étendue).

3) Description des écarts au protocole dans chacun des trois groupes :

    - Sujets inclus à tort (caractéristiques et fréquence).
    - Répartition des motifs d'absence de critères de jugement (perdu de vue, refus, décès, autres motifs) aux 3 temps d'évaluation (M_0, M_3, M_5).

4) Vérification de l'application des programmes d'activité physique et de l'intervention nutritionnelle. Une feuille d'émargement pour la participation aux séances d'activités physiques, le recueil des données d'activité physique, ainsi que la prise des denrées alimentaires, vécu et effets secondaires.

5) Evaluation de l'efficacité :

    Analyse unique, en intention de traiter (selon le groupe attribué par la randomisation).
    Risque global de première espèce = 5 %.
    Formulation bilatérale des tests statistiques.
    Analyse principale : comparaison globale de l'évolution moyenne de la masse grasse $M_5 - M_0$ entre les 3 groupes d'intervention (ANOVA à 1 facteur à 3 niveaux ; risque de première espèce = 5 %). Si au moins une des 3 moyennes diffère significativement des 2 autres, comparaison des groupes 2 à 2 au seuil de significativité 5 % / 3 = 1,67 % pour chacune des 3 comparaisons.
    Analyses secondaires : analyse des données longitudinales recueillies à M_0, M_3 et M_5 (statut glycémique, lipidique, oxydatif et inflammatoire, Lipoxmax) : représentation graphique des évolutions et utilisation des équations estimantes généralisées adaptées aux mesures répétées pour tester les tendances décrites sur la durée du suivi.

11. SURVEILLANCE DE LA RECHERCHE

11.1. CONSEIL SCIENTIFIQUE

11.1.1. Composition

Il est composé des personnes suivantes :

François Favier, Médecin coordinateur CIC-EC, attaché au service d'endocrinologie – maladies métaboliques – diabétologie du GHSR, investigateur principal (président)

Florent Besnier, chargé de projets CIC-EC
Karim Boussaïd, data manager CIC-EC
Jean-Frédéric Brun, PH CERAMM
Georges Dalleau, Pr. CURAPS Université de la Réunion
Xavier Debussche, PH CHR-CHD
Adrian Fianu, statisticien CIC-EC
Véronique Fontaine, diététicienne CHR-GHSR
Patrick Gérardin, PH CHR-GHSR/Délégué CIC-EC
Marie Paule Gonthier, MCF GEICO Université de la Réunion
Eric Jarlet, PH CHR-GHSR
Christian Lefebvre d'Hellencourt, MCF GEICO Université de la Réunion
Nathalie Le Moullec, PH CHR-GHSR
Nadège Naty, coordinatrice CIC-EC
Stéphane Schneebeli, PH CHR-GHSR
Jean Louis Solet, épidémiologiste CIRE Réunion-Mayotte
Chantal Verkindt MCF CURAPS Université de la Réunion

Les observateurs permanents sont : Fidéline Filleul représentant le promoteur.

11.1.2. Rythme des reunions

Le Conseil Scientifique de la recherche se réunit avant le démarrage de la recherche puis au moins une fois par an jusqu'à la clôture de la recherche, dont une fois entre un et deux mois après la date anniversaire de l'avis favorable donné à la recherche par l'autorité de santé pour validation du rapport annuel de sécurité.

11.1.3. Role

- Il a pour mission de prendre toute décision importante à la demande de l'investigateur principal concernant la bonne marche de la recherche et le respect du protocole.
- Il vérifie le respect de l'éthique.
- Il s'informe auprès du Centre de Méthodologie et de Gestion des données et du centre investigateur coordonnateur de la recherche de l'état d'avancement de la recherche, des problèmes éventuels et des résultats disponibles.
- Il décide de toute modification pertinente du protocole nécessaire à la poursuite de la recherche, notamment :
  - les mesures permettant de faciliter le recrutement dans la recherche,
  - les amendements au protocole avant leur présentation au CPP et à l'autorité de santé compétente,
  - les décisions d'ouvrir ou de fermer des sites participant à la recherche,
  - les mesures qui assurent aux personnes participant à la recherche la meilleure sécurité,
  - la discussion des résultats et la stratégie de publication de ces résultats.
- Il approuve la constitution et la composition du Comité Indépendant qui est proposée par l'investigateur principal au cours de ses premières réunions.

- Le Conseil Scientifique peut proposer (après avis du Comité Indépendant) de prolonger ou d'interrompre la recherche en cas de rythme d'inclusion trop lent, d'un trop grand nombre de perdus de vue, de violations majeures du protocole ou bien pour des raisons médicales et/ou administratives. Il précise les modalités éventuelles du suivi prolongé des patients inclus dans la recherche.
- S'il est proposé de réaliser de nouvelles recherches biologiques à partir du matériel de la recherche par les personnes y participant et lorsqu'elles n'ont pas été prévues par le protocole, le Conseil Scientifique les étudie et définit les conditions d'information des patients, d'accès aux données et les règles de publication des résultats.
- En matière de surveillance de la tolérance de la stratégie thérapeutique, le Conseil Scientifique prend connaissance du rapport de tolérance annuel destiné à l'Afssaps et au Comité de Protection des Personnes, le discute et le valide.
- A l'issue de la réunion, le président du Conseil Scientifique doit informer le promoteur des décisions arrêtées. Les décisions concernant un amendement majeur ou une modification de budget doivent être approuvées par le promoteur.

11.2. CENTRE DE METHODOLOGIE ET DE GESTION DES DONNEES

Le Centre de Méthodologie et de Gestion des données est le CIC-EC de la Réunion (méthodologiste de la recherche : François Favier).

11.2.1. COMPOSITION

L'équipe projet est composée d'un méthodologiste, d'un statisticien, d'un chargé d'étude, d'une coordinatrice d'enquête, d'un agent de saisie et d'un data manager.

11.2.2. RYTHME DES REUNIONS

Des réunions régulières de l'équipe projet permettent de suivre l'avancement de la recherche.

11.2.3. ROLE

Le Centre de Méthodologie et de Gestion des données :

- collabore à la conception du protocole avec l'investigateur principal et supervise la conception méthodologique de la recherche,
- finalise la rédaction du protocole, des notes d'information et formulaires de consentement et du cahier d'observation avant soumission au CPP et à l'Afssaps,
- réalise et gère la base de données informatique dédiée à la recherche,
- prépare en collaboration avec l'ARC du promoteur la mise en place et le suivi de la recherche, coordonne la logistique de la recherche,
- effectue l'analyse statistique des données,
- participe aux publications et autres valorisations des résultats de la recherche,

Le Centre de Méthodologie et de Gestion des données, participe à la présentation de l'état d'avancement de la recherche au Conseil Scientifique.

11.3. CENTRE INVESTIGATEUR COORDONNATEUR

Le centre investigateur coordonnateur est le Service d'endocrinologie – maladies métaboliques – diabétologie du Groupe Hospitalier Sud Réunion. Le centre coordonnateur est chargé de l'organisation et de la logistique du projet. Il informe le Conseil Scientifique du déroulement de la recherche, prépare les réunions du Conseil Scientifique en collaboration avec le Centre de Méthodologie et de Gestion des données (CIC-EC).

12. DROITS D'ACCES AUX DONNEES ET DOCUMENTS SOURCE

12.1. ACCES AUX DONNEES

Le promoteur est chargé d'obtenir l'accord de l'ensemble des parties impliquées dans la recherche afin de garantir l'accès direct à tous les lieux de déroulement de la recherche, aux données source, aux documents source et aux rapports dans un but de contrôle de qualité et d'audit par le promoteur.
Les investigateurs mettront à disposition les documents et données individuelles strictement nécessaires au suivi, au contrôle de qualité et à l'audit de la recherche biomédicale, à la disposition des personnes ayant un accès à ces documents conformément aux dispositions législatives et réglementaires en vigueur (articles L.1121-3 et R.5121-13 du code de la santé publique).

12.2. DONNEES SOURCE

Tout document ou objet original permettant de prouver l'existence ou l'exactitude d'une donnée ou d'un fait enregistrés au cours de la recherche est défini comme document source.

Documents source : original des résultats d'examens biologiques, compte-rendu d'épreuve d'effort, cahiers de suivi de l'activité physique.

12.3. CONFIDENTIALITE DES DONNEES

Conformément aux dispositions législatives en vigueur (articles L.1121-3 et R.5121-13 du code de la santé publique), les personnes ayant un accès direct aux données source prendront toutes les précautions nécessaires en vue d'assurer la confidentialité des informations relatives aux procédures expérimentales, aux recherches, aux personnes qui s'y prêtent et notamment en ce qui concerne leur identité ainsi qu'aux résultats obtenus. Ces personnes, au même titre que les investigateurs eux-mêmes, sont soumises au secret professionnel.
Pendant la recherche biomédicale ou à son issue, les données recueillies sur les personnes qui s'y prêtent et transmises au promoteur par les investigateurs (ou tous autres intervenants spécialisés) seront rendues anonymes. Elles ne doivent en aucun cas faire apparaître en clair les noms des personnes concernées ni leur adresse.
Modalités de codification des sujets : seules la première lettre du nom et du prénom du sujet seront enregistrées, accompagnées d'un numéro codé propre à la recherche indiquant l'ordre d'inclusion des sujets.
Le promoteur s'assurera que chaque personne qui se prête à la recherche a donné son accord par écrit pour l'accès aux données individuelles la concernant et strictement nécessaires au contrôle de qualité de la recherche.

13. CONTROLE ET ASSURANCE QUALITE

13.1. CONSIGNES POUR LE RECUEIL DES DONNEES

Toutes les informations requises par le protocole doivent être consignées sur les cahiers d'observation et une explication doit être apportée pour chaque donnée manquante. Les données devront être recueillies au fur et à mesure qu'elles sont obtenues, et transcrites dans ces cahiers de façon nette et lisible.
Les données erronées relevées sur les cahiers d'observation seront clairement barrées et les nouvelles données seront copiées, à côté de l'information barrée, accompagnées des initiales, de la date et éventuellement d'une justification par l'investigateur ou la personne autorisée qui aura fait la correction.
Les données seront recueillies sur un cahier d'observation papier.

13.2. SUIVI DE LA RECHERCHE

Le suivi de la recherche sera assuré par une coordinatrice d'enquête. Elle sera chargée, auprès de l'investigateur principal, de :

- la logistique et la surveillance de la recherche,
- l'établissement des rapports concernant son état d'avancement,
- la vérification de la mise à jour du cahier d'observation (demande d'informations complémentaires, corrections,…)
- la transmission des EIG au promoteur.

Elle travaillera conformément aux procédures opératoires standardisées, en collaboration avec l'attaché de recherche clinique délégué par le promoteur.

13.3. CONTROLE DE QUALITE

Un attaché de recherche clinique mandaté par le promoteur visite de façon régulière chaque centre investigateur, lors de la mise en place de la recherche, une ou plusieurs fois en cours de recherche selon le rythme des inclusions et en fin de recherche. Lors de ces visites, les éléments suivants seront revus :

- consentement éclairé,
- respect du protocole de la recherche et des procédures qui y sont définies,
- qualité des données recueillies dans le cahier d'observation : exactitude, données manquantes, cohérence des données avec les documents source (dossiers médicaux, carnets de rendez-vous, originaux des résultats de laboratoire, etc,…),
- gestion des produits éventuels.

Toute visite fera l'objet d'un rapport de monitorage par compte-rendu écrit.

13.4. GESTION DES DONNEES

Les données sont recueillies sur des cahiers d'observation papier qui sont colligés par la coordinatrice qui vérifiera la complétude des questionnaires et la cohérence.
- les modalités de saisie des données : simple saisie avec relecture et contrôle de qualité, par le CIC-EC, sur Epidata. Personnels responsables : agent de saisie, coordinatrice d'enquête, data manager.
- le processus de validation des données : vérifications logiques à la saisie (Epidata) par l'agent de saisie, vérifications aléatoires après saisie sur 10% de dossiers par le data manager. Si le % d'erreurs est >1%, une double saisie est réalisée. Exportation sous SAS et vérifications de la base de données par le data manager. Les données sont validées conformément au plan de data management défini conjointement entre l'investigateur principal et le Centre de Méthodologie et de Gestion des données (méthodologiste, data manager et statisticien).

Le gel de la base est décidé conjointement par le statisticien et l'investigateur principal. Le processus de gel/dégel des données est réalisé conformément à la procédure mise en place dans le Centre de Méthodologie et de Gestion des données (gel des données brutes au format XML et sous forme de table SAS).

Les données saisies seront sauvegardées quotidiennement dans l'ordinateur et sur disque dur externe. Les données informatiques seront conservées 15 ans.

13.5. AUDIT ET INSPECTION

Un audit peut être réalisé à tout moment par des personnes mandatées par le promoteur et indépendantes des responsables de la recherche. Il a pour objectif de s'assurer de la qualité de la recherche, de la validité de ses résultats et du respect de la loi et des réglementations en vigueur.
Les investigateurs acceptent de se conformer aux exigences du promoteur et à l'autorité compétente en ce qui concerne un audit ou une inspection de la recherche.
L'audit pourra s'appliquer à tous les stades de la recherche, du développement du protocole à la publication des résultats et au classement des données utilisées ou produites dans le cadre de la recherche.

14. CONSIDERATIONS ETHIQUES ET REGLEMENTAIRES

Le promoteur et les investigateurs s'engagent à ce que cette recherche soit réalisée en conformité avec la loi n°2004-806 du 9 août 2004, ainsi qu'en accord avec les Bonnes Pratiques Cliniques (I.C.H. version 4 du 1er mai 1996 et décision du 24 novembre 2006) et la déclaration d'Helsinki (qui peut être retrouvée dans sa version intégrale sur le site http://www.wma.net/f/policy/b3.htm).
La recherche est conduite conformément au présent protocole. Hormis dans les situations d'urgence nécessitant la mise en place d'actes thérapeutiques précis, les investigateurs s'engagent à respecter le protocole en tous points en particulier en ce qui concerne le recueil du consentement et la notification et le suivi des événements indésirables graves.

Cette recherche a reçu l'avis favorable du Comité de Protection des Personnes Sud-Ouest et Outre Mer III le 31.08.2011 (amendement N°1 le 26.10.2011) et l'autorisation de l'Afssaps le 15.06.2011.

Le CHR de la Réunion, promoteur de cette recherche, a souscrit un contrat d'assurance en responsabilité civile auprès de la SHAM conformément aux dispositions de l'article L1121-10 du code de la santé publique.

Les données enregistrées à l'occasion de cette recherche font l'objet d'un traitement informatisé au Centre d'Investigation Clinique - Epidémiologie Clinique de la Réunion dans le respect de la loi n°78-17 du 6 janvier 1978 relative à l'informatique, aux fichiers et aux libertés modifiée par la loi 2004-801 du 6 août 2004.
Cette recherche entre dans le cadre de la « Méthodologie de référence » (MR-001) en application des dispositions de l'article 54 alinéa 5 de la loi du 6 janvier 1978 modifiée relative à l'information, aux fichiers et aux libertés. Ce changement a été homologué par décision du 5 janvier 2006. Le CHR de la Réunion a signé un engagement de conformité à cette « Méthodologie de référence » n°1350038 en date du 06 mars 2009.

Cette recherche est enregistrée sur le site http://clinicaltrials.gov/ sous le n° NCT01464073

La collection d'échantillons biologiques réalisée dans le cadre de cette recherche a été déclarée à l'Afssaps en même temps que la demande d'autorisation de la recherche.

AMENDEMENT AU PROTOCOLE

Toute modification substantielle, c'est à dire toute modification de nature à avoir un impact significatif sur la protection des personnes, sur les conditions de validité et sur les résultats de la recherche, sur la qualité et la sécurité des produits expérimentés, sur l'interprétation des documents scientifiques qui viennent appuyer le déroulement de la recherche ou sur les modalités de conduite de celle-ci, fait l'objet d'un amendement écrit qui est soumis au promoteur ; celui-ci doit obtenir, préalablement à sa mise en œuvre, un avis favorable du CPP et une autorisation de l'Afssaps.
Les modifications non substantielles, c'est à dire celles n'ayant pas d'impact significatif sur quelque aspect de la recherche que ce soit, sont communiquées au CPP à titre d'information.
Tous les amendements au protocole doivent être portés à la connaissance de tous les investigateurs qui participent à la recherche. Les investigateurs s'engagent à en respecter le contenu.

Tout amendement qui modifie la prise en charge des patients ou les bénéfices, risques et contraintes de la recherche fait l'objet d'une nouvelle note d'information et d'un nouveau formulaire de consentement dont le recueil suit la même procédure que celle précitée.

15. CONSERVATION DES DOCUMENTS ET DES DONNEES RELATIVES A LA RECHERCHE

Les documents suivants relatifs à cette recherche sont archivés conformément aux Bonnes Pratiques Cliniques et à la réglementation en vigueur :

- Par les médecins investigateurs :
  - **pour une durée de 15 ans suivant la fin de la recherche** (recherches portant sur des médicaments, des dispositifs médicaux ou des dispositifs médicaux de diagnostic in vitro ou recherches ne portant pas sur un produit mentionné à l'article L.5311-1 du code de la santé publique)
    - Le protocole et les amendements éventuels au protocole
    - Les cahiers d'observation
    - Les dossiers source des participants ayant signé un consentement
    - Tous les autres documents et courriers relatifs à la recherche
  - **pour une durée de 30 ans suivant la fin de la recherche** (tous les autres types de recherches)
    - L'exemplaire original des consentements éclairés signés des participants

      Tous ces documents sont sous la responsabilité de l'investigateur pendant la durée réglementaire d'archivage.

- Par le promoteur :
  - **pour une durée de 15 ans suivant la fin de la recherche** (recherches portant sur des médicaments, des dispositifs médicaux ou des dispositifs médicaux de diagnostic in vitro ou recherches ne portant pas sur un produit mentionné à l'article L.5311-1 du code de la santé publique)
    - Le protocole et les amendements éventuels au protocole
    - L'original des cahiers d'observation
    - Tous les autres documents et courriers relatifs à la recherche
  - **pour une durée de 30 ans suivant la fin de la recherche** (tous les autres types de recherches)
    - Un exemplaire des consentements éclairés signés des participants
    - Les documents relatifs aux événements indésirables graves

Tous ces documents sont sous la responsabilité du promoteur pendant la durée réglementaire d'archivage.
Aucun déplacement ou destruction ne pourra être effectué sans l'accord du promoteur. Au terme de la durée réglementaire d'archivage, le promoteur sera consulté pour destruction. Toutes les données, tous les documents et rapports pourront faire l'objet d'audit ou d'inspection.

16. REGLES RELATIVES A LA PUBLICATION

16.1. COMMUNICATIONS SCIENTIFIQUES

L'analyse des données est réalisée par le Centre d'Investigation Clinique - Epidémiologie Clinique de la Réunion. Cette analyse donne lieu à un rapport écrit qui est soumis au promoteur, qui transmettra au Comité de Protection des Personnes et à l'autorité compétente.

Toute communication écrite ou orale des résultats de la recherche doit recevoir l'accord préalable de l'investigateur principal et, le cas échéant, du Conseil Scientifique.

La publication des résultats principaux mentionne le nom du promoteur, de tous les investigateurs ayant inclus ou suivi des patients dans la recherche, des personnes ayant réalisé la recherche, des membres du Conseil Scientifique et la source de financement. Il sera tenu compte des règles internationales d'écriture et de publication (Convention de Vancouver, février 2006).

16.2. COMMUNICATION DES RESULTATS AUX PATIENTS

Conformément à la loi n°2002-303 du 4 mars 2002, les patients sont informés, à leur demande, des résultats globaux de la recherche. Dès la fin de l'intervention, l'analyse des données sur le critère de jugement principal sera mise en route afin de déterminer quelle est, des trois groupes étudiés, celui où l'activité physique a été la plus efficace pour la perte de masse grasse. Ces résultats seront présentés à l'ensemble des participants et un accompagnement par l'éducateur sportif sera effectué afin de permettre à tous de poursuivre les activités physiques en autonomie, selon le programme du groupe qui aura donné les meilleurs résultats (1, 2 ou 3).

16.3. CESSION DES DONNEES

Le recueil et la gestion des données sont assurés par le Centre d'Investigation Clinique - Epidémiologie Clinique de la Réunion. Les conditions de cession de tout ou partie de la base de données de la recherche sont décidées par le promoteur de la recherche et font l'objet d'un contrat écrit.

RÉFÉRENCES BIBLIOGRAPHIQUES

ACC/AHA (2002) Guideline Update for Exercise Testing : Summary Article : A report of the American College of Cardiology / American Heart Association Task Force on Practice Guidelines (Committee to Update the 1997 Exercise testing Guidelines). Circulation 106: 1883-1892.

ACSM/AHA. Haskell WL, Lee IM, Pate RR, Powell KE, Blair SN, Franklin BA, et al. (2007) Physical activity and public health: updated recommendations for adults from the American College of Sports Medicine and the American Heart Association. Circulation.;116(9):1081–93.

Aeberli I, Molinari L, Spinas G, et al. (2006) Dietary intakes of fat and antioxidant vitamins are predictors of subclinical inflammation in overweight Swiss children. Am J Clin Nutr. Oct;84(4):748-55.

ALFEDIAM, Recommandations (1998) Jean-François Gautier, Catherine Berne, Jean-Jacques Grimm, Beatrice Lobel, Vincent Coliche, Etienne Mollet.

American College of Sports Medicine (2009) ACSM'S Exercise Management for persons with chronic diseases and disabilities. Third Edition. Pages 182-200.

Arnlöv J, Ingelsson E, Sundström J, Lind L (2010) Impact of body mass index and the metabolic syndrome on the risk of cardiovascular disease and death in middle-aged men. Circulation,121(2):230-6.

Arsenault BJ, Després JP, Stroes ES, et al. (2010) Lipid assessment, metabolic syndrome and coronary heart disease risk. Eur J Clin Invest.,40(12):1081-93.

Ballor DL, McCarthy JP, Wilterdink EJ. (1990) Exercise intensity does not affect the composition of diet- and exercise-induced body mass loss. Am J Clin Nutr. Feb;51(2):142-6.

Baromètre Santé (2005), INPES, Attitudes et comportements de santé.

Ben Ounis O, Elloumi M, Amri M et al. (2009) Impact of training and hypocaloric diet on fat oxidation and body composition in obese adolescents. Science & Sports 24 :178–185.

Ben Ounis O, Elloumi M, Ben Chiek I et al. (2008) Effect of two month physical endurance and diet-restriction programmes on lipid profiles and insulin resistance in obese adolescent boys. Diabetes met 34:595-600.

Bigard A, Duforez F, Portero P, Guezennec C. (1992) Détermination de l'activité physique par questionnaire : validation du questionnaire autoadministrable de Baecke. Sci Sport;7:215–21.

Boden G. (2011) Obesity, insulin resistance and free fatty acids. Curr Opin Endocrinol Diabetes Obes,18(2):139-43.

Bordenave S, Metz L, Flavier S al. (2008) Training-induced improvement in lipid oxidation in type 2 diabetes mellitus is related to alterations in muscle mitochondrial activity. Effect of endurance training in type 2 diabetes, Diabetes Metab. 34 162-168.

Bouchard C et Rankinen T (2001) Individual differences in response to regular physical activity. Medicine and Science in Sports and Exercise, vol. 33, no. 6, pp. S446–S451.

Boutcher SH. (2011) High-intensity intermittent exercise and fat loss. J Obes. 2011;2011:868305.

Brandou F, Dumortier M, Garandeau P et al. (2003) Effects of two month rehabilitation program on substrate utilization during exercise in obese adolescents. Diabetes metab 29:20-7.

Brandou F, Savy-Pacaux AM, Marie J et al. (2005) Impact of high- and low-intensity targeted exercise training on the type of substrate utilization in obese boys submitted to a hypocaloric diet, Diabetes Metab. 31:327-335.

Bruun JM et al. (2005) Diet and exercise reduce low-grade inflammation and macrophage infiltration in adipose tissue but not in skeletal muscle in severely obese subjects. Am. J. Physiol. Endocrinol. Metab. 290: E961-E967.

Brun JF, Jeana E, Ghanassia E, Flaviera S, Mercier J (2007) Metabolic training: new paradigms of exercise training for metabolic diseases with exercise calorimetry targeting individuals. Annales de réadaptation et de médecine physique. 50 :528–534.

Brun J, Maurie J, Jean E, et al. (2010) Comparison of Square-Wave Endurance Exercise Test (SWEET) training with endurance training targeted at the level of maximal lipid oxidation in type 2 diabetics, Diabetologia. 53.

Brun JF, Romain AJ, Mercier J (2011) Maximal lipid oxydation during exercise (LIPOXmax): from physiological measurements to clinical applications. Facts and uncertainties. Science and Sports 26;57-71.

Canoy D, Wareham N, Welch A, et al. (2005) Plasma ascorbic acid concentrations and fat distribution in 19,068 British men and women in the European Prospective Investigation into Cancer and Nutrition Norfolk cohort study. Am J Clin Nutr. 2005 Dec;82(6):1203-9.

Carré F. (2005) Obésité abdominale, syndrome métabolique et risque cardiovasculaire. L'engrenage. Cardinale, revue de cardiologie. Collection interdisciplinaire n°8.

Carrière A. Carmona MC, Fernandez Y et al. (2004) Mitochondrial reactive oxygen species control the transcription factor CHOP-10/GADD 153 and adipocyte differentiation: a mechanism for hypoxia-dependent effect. J. Biol. Chem. 279(39): 40462-40469.

Charles LE et al. (2008) Adiposity measures and oxidative stress among Police officers. Obesity, 16: 2489-2497.

Chesnes S. et al. (2006) Effects of oxidative modifications induced by the glycation of bovin serum albumin on its structure and on cultured adipose cells. Biochimie, 88(10): 1467-1477.

De Ferranti S, Mozaffarian D. (2008) The perfect storm: obesity, adipocyte dysfunction and metabolic consequences. Clin. Chem., 54(6): 945-955.

Després JP, Lemieux I. (2006) Abdominal obesity and metabolic syndrome. Nature, 444 (7121): 881-887.

Després JP (2007) L'obésité abdominal, une maladie métabolique. John Libbey Eurotext Edition.

Douard H, Mora B, Broustet JP. (1987) Epreuve d'effort et tachycardie ventriculaire : l'expérience française. Arch Mal Cœur Vais;80: 263-270.

Drolet R. et al. (2008) Hypertrophy and hyperplasia of abdominal adipose tissues in women. Int. J. Obes. (London). 32(2): 283-291.

Dumortier M, Brandou F, Perez-Martin A et al. (2003) Low intensity endurance exercise targeted for lipid oxidation improves body composition and insulin sensitivity in patients with the metabolic syndrome. Diabetes Metab;29:509–18.

Elloumi M, Ben Ounis O, Makni E et al. (2009) Effect of individualized weight-loss programmes on adiponectin, leptin and resistin levels in obese adolescent boys, Acta Paediatr. 98 1487-1493.

Favier F, Rachou E, Ricquebourg M, Fianu A. (2002) Comportements alimentaires et activité physique des Réunionnais. Etude RECONSAL. Saint-Denis de La Réunion, INSERM-ORS.

Favier F, Jaussent I, Le Moullec N, Debussche X, Boyer MC, Schwager JC, Papoz L and the REDIA Study Group. (2005) Prevalence of Type 2 Diabetes and central adiposity in Reunion island, a population in epidemiologic transition: The REDIA Study. Diabetes Res Clin Pract, 67,234-42.

Favier F, Fianu A, Naty N et al. (2005) Trial for the primary prevention of type-II diabetes mellitus and the metabolic syndrome in la Réunion. Rev Med Ass Maladie;36(1):5-13.

Fisher G, Hyatt TC, Hunter GR et al. (2010) Effect of Diet With and Without Exercise Training on Markers of Inflammation and Fat Distribution in Overweight Women. Obesity (Silver Spring). Dec 23.

Fito M, Guxens M, Corella D, et al. (2007) Effect of a traditional Mediterranean diet on lipoprotein oxidation : a randomized controlled trial. Arch Intern Med. 167(11):1195-203.

Gómez-Ambrosi J, Silva C, Galofré JC, Escalada J, Santos S, et al. (2011) Body mass index classification misses subjects with increased cardiometabolic risk factors related to elevated adiposity. Int J Obes (Lond). 2011 May 17. [Epub ahead of print].

Gomez-Cabrera MC, Domenech E, Viña J. (2008) Moderate exercise is an antioxidant: upregulation of antioxidant genes by training. Free Radic Biol Med. Jan 15;44(2):126-31. Epub 2007 Feb 9.

Gonthier MP, Verny MA, Besson C, et al. (2003) Chlorogenic acid bioavailability largely depends on its metabolism by the gut microflora in rats. J Nutr.,133(6):1853-9.

Ito H, Gonthier MP, Manach C, et al. (2005) Polyphenol levels in human urine after intake of six different polyphenol-rich beverages. Br J Nutr.,94(4):500-9.

Gonthier MP, Remesy C, Scalbert A, et al. (2006) Microbial metabolism of caffeic acid and its esters chlorogenic and caftaric acids by human faecal microbiota in vitro. Biomed Pharmacother.,60(9):536-40.

Gonthier MP. et al. (2007) Identification of endocannabinoids and related compounds in human fat cells. Obesity, 15(4): 837-845.

Gordon NF, Scott CB. Exercise intensity prescription in cardiovascular disease. Theoretical basis for anaerobic threshold determination. J Cardiopulm Rehabil 1995;15(3):193–6.

Grediagin A, Cody M, Rupp J, (1995) Exercise intensity does not effect body composition change in untrained, moderately overfat women. J Am Diet Assoc.;95(6):661-5.

Gregor M,Hotamisligil GS. (2007) Thematic review series: adipocyte biology. Adipocyte stress: the endoplasmic reticulum and metabolic disease. J. Lipid Res., 48(9): 1905-1914.

Harding JJ,Beswick HT. (1988) The possible contribution of glucose autoxidation to protein modification of diabetes. Biochem. J., 249(2): 617-618.

Harnack LJ, Jeffery RW, Boutelle KN. (2000) Temporal trends in energy intake in the United States: an ecologic perspective. Am J Clin Nutr.;71(6):1478-84.

Hercberg S, Galan P et al. (2004) The SUVIMAX study, a randomized placebo controlled trial of the health effects of antioxidant vitamins and minerals. Arch Intern Med 164(21): 2335-42.

Hind K, Oldroyd B, Truscott JG (2010) In vivo precision of the GE Lunar iDXA densitometer for the measurement of total body composition and fat distribution in adults. European Journal of Clinical Nutrition 1–3.

Hofmann P et Tschakert G (2010) Special Needs to Prescribe Exercise Intensity for Scientific Studies. Cardiology Research and Practice. Volume 2011, Article ID 209302, 10 pages doi:10.4061/2011/209302

Houstis N. et al. (2006) Reactive oxygen species have a causal role in multiple forms of insulino-resistance. Nature, 440 (7086): 944-948.

Hu FB, Li TY, Colditz GA, et al. (2003) Television watching and other sedentary behaviors in relation to risk of obesity and type 2 diabetes mellitus in women. JAMA;289(14):1785-91.

Irving BA, Davis CK, Brock DW, et al. (2008) Effect of exercise training intensity on abdominal visceral fat and body composition. Med Sci Sports Exerc.;40(11):1863-72.

Kennedy AL, Lyons TJ. (1997) Glycation, oxidation, and lipoxidation in the development of diabetic complications. Metabolism, 46(12 Suppl. 1): 14-21.

Kern P.A. et al. (1995) The expression of tumor necrosis factor in human adipose tissue. Regulation by obesity, weight loss, and relationship to lipoprotein lipase. J Clin Invest. 95, 2111-9.

Knowler WC, Barett-Connor E, Fowler SE et al. (2002) Reduction in the incidence of type 2 diabetes with lifestyle intervention or metformin. N Engl J Med. 346:393-403.

Kosaka K, Noda M, Kuzuya T (2005) Prevention of type 2 diabetes by lifestyle intervention : a Japanese trial in IGT males. Diabetes Res Clin Pract. 67:152-162.

Krieger-Brauer HI. et al. (2000) Inhibitory effect of isoproterenol on NADPH-dependent H(2)O(2) generation in human adipocyte plasma membranes is mediated by betagamma-subunits derived from G(s). J. Biol. Chem., 275(4): 2486-2490.

Kriska AM, La Porte RE, Pettitt DJ et al. (1993) The association of physical activity with obesity, fat distribution and glucose intolerance in Pima Indians. Diabetologia, 36, 863-9.

Lairon D, Arnault N, Bertrais S, et al. (2005) Dietary fiber intake and risk factors for cardiovascular disease in French adults. Am J Clin Nutr. Dec;82(6):1185-94.

Lazzer S, Lafortuna C, Busti C, et al. (2011) Effects of Low and High intensity exercise training on body composition and substrate metabolism in obese adolescents. J. Endocrinol. Invest. 34, 45-52, 2011.

Li G, Zhang P, Wang J, Gregg EW et al. (2008) The long-term effect of lifestyle interventions to prevent diabetes in the China Da Qing Diabetes Prevention Study: a 20-year follow-up study. The Lancet. 371:1783-1789.

Lin Y. et al. (2005) The hyperglycemia-induced inflammatory response in adipocytes: the role of reactive oxygen species. J. Biol. Chem., 280(6): 4617-4626.

Lwow F, Dunajska K, Tworowska U et al. (2007) Postexercise oxidative stress and obesity in postmenopausal women: the role of beta3-adrenergic receptor polymorphism. Gynecol Endocrinol. 23(10):597-603.

Maskarinec G, Novotny R, Tasaki K. (2000) Dietary patterns are associated with body mass index in multiethnic women. J Nutr. 2000 Dec;130(12):3068-72.

Matias I. et al. (2006) Regulation, function and dysregulation of endocannabinoids in models of adipose and beta-pancreatic cells in obesity and hyperglycemia. J. Clin. Endocr. Metab., 91(8): 3171-3180.

Meyer T, Lucia A, Earnest CP et Kindermann W (2005) A conceptual framework for performance diagnosis and training prescription from submaximal gas exchange parameters - theory and application," International Journal of Sports Medicine, vol. 26, no. 1, pp. S38–S48.

Mougios V, Kazaki M, Christoulas K, et al. (2006) Does the Intensity of an Exercise Programme Modulate Body Composition Changes? Int J Sports Med. 2006 Mar;27(3):178-81.

Moor de Burgos A, Wartanowicz M, Ziemlański S. (1992) Blood vitamin and lipid levels in overweight and obese women. Eur J Clin Nutr. Nov;46(11):803-8.

Nassis et al. (2005) Aerobic exercise training improves insulin sensitivity without changes in body weight, body fat, adiponectin, and inflammatory markers in overweight and obese girls. Metab. Clin. Exper., 54: 1472-1479.

Nathan DM, Buse JB, Davidson MB et al. (2006) Management of hyperglycemia in type 2 diabetes : a consensus algorithm for the initiation and adjustement of therapy: a consensus statement from the American Diabetes Association and the European Association for the Study of Diabètes. Diabetes Care, 29:1963-1972.

Nicklas BJ, Wang X, You T, et al. (2009) Effect of exercise intensity on abdominal fat loss during calorie restriction in overweight and obese postmenopausal women: a randomized, controlled trial. Am J Clin Nutr.;89(4):1043-52.

ObEpi (2009) 5ème Enquête épidémiologique nationale sur le surpoids et l'obésité

Organisation Mondiale de la Santé. Diet, nutrition and the prevention of chronic diseases. Report of a joint FAO/WHO Expert Consultation 2003, Technical Report Series. N°916.

Organisation Mondiale de la Santé : Le diabète, aide mémoire N°312, Janvier 2011

Pedersen PK et al. (2001) Muscle-derived interleukin-6: possible biological effects. J. Physiol. 536: 329-337.

Perez-Martin A, Dumortier M, Raynaud E et al. (2001) Balance of substrate oxidation during submaximal exercise in lean and obese people. Diabetes Metab;27:466–74.

Perri MG, Anton SD, Durning PE, et al. (2002) Adherence to exercise prescriptions: effects of prescribing moderate versus higher levels of intensity and frequency. Health Psychol. 2002 Sep;21(5):452-8.

Pincemail J, Vanbelle S, Degrune F, et al. (2011) Lifestyle Behaviours and Plasma Vitamin C and β-Carotene Levels from the ELAN Population (Liège, Belgium). J Nutr Metab.;2011:494370. Epub 2011 Mar 6.

Programme National Nutrition Santé (PNNS) Oppert JM, Simon S, Rivière D, Guezennec CY. Activité physique et Santé, arguments scientifiques, pistes pratiques.

Ramachandran A, Snehalatha C, Mary S et al. (2006) The Indian Diabetes Prevention Programme shows that lifestyle modification and metformin prevent type 2 diabetes in Asian Indian subjects with impaired glucose tolerance (IDPP-1). Diabetologia. 49:289-297.

Rana JS, Li TY, Manson JE and Hu FB. (2007) Adiposity compared with physical inactivity and risk of type 2 diabetes in women. Diabetes Care 30: 53-58.

Regensteiner JG, Mayer EJ, Sheterly SM et al. (1991) Relation between habitual physical activity and hyperinsulinemia among non diabetic men and women. The San Luis Valley diabetes study. Diabetes care, 14, 1066-74.

Rey-López JP, Vicente-Rodríguez G, Biosca M, et al. (2008) Sedentary behaviour and obesity development in children and adolescents. Nutr Metab Cardiovasc Dis.;18(3):242-51.

Riccardi G, Giacco R, Rivellese AA. (2004) Dietary fat, insulin sensivity and the metabolic syndrome. Clin Nutr, 23, 447-56.

Ricci P, Boltière PO, Weill A, et al. (2010) Diabète traité en France : quelles évolutions entre 2000 et 2009. Bull Epidemiol Hebd;42-43:425-31.

Romain A, Fedou C, Mercier J, et al. (2010) exercise targeted at the level of maximal lipid oxidation in overweight and obesity: a meta-analysis, Obes Rev. 11:229.

Salvadego D, Lazzer S, Busti C et al. (2010) Gas exchange kinetics in obese adolescents. Inferences on exercise tolerance and prescription. American Journal of Physiology, vol. 299, no. 5, pp. R1298–R1305.

Scharhag-Rosenberger F, Meyer T, G¨aßler N et al. (2010) Exercise at given percentages of VO_{2max}: heterogeneous metabolic responses between individuals. Journal of Science andMedicine in Sport, vol. 13, no. 1, pp. 74–79.

Schulz LO, Bennett PH, Ravussin E, Kidd JR et al. (2006) Effects of traditional and western environnements on prevalence of type 2 diabetes in Pima Indians in Mexico and the US. Diabetes Care. 29, 1866-1871.

Silverman NE et al. (2009) Addition of aerobic exercise to a weight loss program increases BMD, with an associated reduction in inflammation in overweight postmenopausal women. Calcif. Tissue Int., 84(4): 257-265.

Skinner JS, Gaskill SE, Rankinen T et al. (2003) Heart rate versus %VO_{2max}: age, sex, race, initial fitness, and training response - HERITAGE. Medicine and Science in Sports and Exercise, vol. 35, no. 11, pp. 1908–1913.

Slentz CA, Duscha BD, Johnson JL, et al. (2004) Effects of the amount of exercise on body weight, body composition, and measures of central obesity: STRRIDE--a randomized controlled study. Arch Intern Med.;164(1):31-9.

Tabet JY, Meurin P, Ben Driss A, Thabut G, Weber H, Renaud N, Odjinkem N, Solal AC (2006) Determination of exercise training heart rate in patients on beta-blockers after myocardial infarction. Eur J Cardiovasc Prev Rehabil. 2006 Aug;13(4):538-43.

Tremblay A, Simoneau JA, Bouchard C. Impact of exercise intensity on body fatness and skeletal muscle metabolism. Metabolism. 1994 Jul;43(7):814-8.

Tuomilehto J, Lindstrom J, Eriksson JG et al. (2001) Prevention of type 2 diabetes mellitus by changes in lifestyle among subjects with impaired glucose tolerance. N Engl J Med. 344:1343-1350.

Van Aggel-Leijssen DP, Saris WH, Wagenmakers AJ, et al. (2001) The effect of low-intensity exercise training on fat metabolism of obese women. Obes Res. Feb;9(2):86-96.

Van Harmelen V. et al. (2003) Effect of BMI and age on adipose tissue cellularity and differentiation capacity in women. Int. J. Obes. Relat. Metab. Disord. 27(8): 89-895.

Van Helvoort HA, De Boer RC, Van de Broek L, et al. (2011) Exercises commonly used in rehabilitation of patients with chronic obstructive pulmonary disease: cardiopulmonary responses and effect over time. Arch Phys Med Rehabil. Jan;92(1):111-7. Epub 2010 Nov 18.

Veilleux A, Caron-Jobin M, Noël S, et al. (2011) Visceral Adipocyte Hypertrophy is Associated With Dyslipidemia Independent of Body Composition and Fat Distribution in Women. Diabetes, 60(5):1504-11.

Venables MC, Jeukendrup AE. (2008) Endurance training and obesity: effect on substrate metabolism and insulin sensitivity. Med Sci Sports Exerc. Mar;40(3):495-502.

Vincent HK, Morgan JW, Vincent KR. (2004) Obesity exacerbates oxidative stress levels after acute exercise. Med Sci Sports Exerc. ;36(5):772-9.

You T et al. (2004) Effect of hypocaloric diet and exercise training on inflammation and adipocyte lipolysis in obese postmenopausal women. J. Clin. Endocrinol. Metab. 89(4):1739-46.

Ware J (1993) SF-36 Health Survey: Manual and Interpretation Guide. Boston, MA, Health Institute.

Ware JE, Kosinski M, Keller SD (1994) SF-36 Physical and Mental Health Summary Scales: A User's Manual (ed 2). Boston, MA: Health Institute.

Youssef H, Groussard C, Pincemail J, et al. (2009) Exercise-induced oxidative stress in overweight adolescent girls: roles of basal insulin resistance and inflammation and oxygen overconsumption. Int J Obes (Lond). 2009 Apr;33(4):447-55.

ANNEXES

ANNEXE 1 : CENTRE INVESTIGATEUR

Version 2.1 du 10.10.2011

CENTRE		LISTE DES INVESTIGATEURS	
N°	NOM ET ADRESSE COMPLETE		
1	**Service d'endocrinologie – maladies métaboliques - diabétologie**<br>CHR site du GHSR – 97448 Saint Pierre Cedex	**Investigateur principal :**<br>Dr. François Favier	Mail : francois.favier@chr-reunion.fr<br>Tel : 0262 71 98 29
		**Co-investigateurs :**<br>Dr. Stéphane Schneebeli<br>Dr. Eric Jarlet<br>Dr. Patrick Gérardin	Mail : stephane.schneebeli@chr-reunion.fr<br>Mail : eric.jarlet@chr-reunion.fr<br>Tel : 0262 35 90 00 poste : 5 59 52<br>Mail : patrick.gerardin@chr-reunion.fr<br>Tel : 0262 71 98 29

ANNEXE 2 : NOTE D'INFORMATION ET FORMULAIRE DE CONSENTEMENT

NOTE D'INFORMATION

« Evaluation de l'efficacité d'un programme d'activité physique individualisé, associé à une alimentation équilibrée enrichie en fruits et légumes, sur l'évolution de la masse grasse chez des femmes en surpoids ou obèses, dans le cadre de la prévention du diabète de type 2 à la Réunion. Etude LIPOXmax-Réunion.»

CODE PROMOTEUR : 2011/CHR/01

Amendement n°1, version n°2.1 du 10/10/2011

Promoteur de la recherche : **CHR de La Réunion – Groupe Hospitalier Sud Réunion**

Investigateur principal : **Dr François Favier**

Madame,

Le Docteur François FAVIER médecin investigateur, Centre d'Investigation Clinique – Epidémiologie Clinique de la Réunion (CIC-EC) et médecin attaché au service d'endocrinologie – maladies métaboliques – diabétologie du GHSR, vous propose de participer à la recherche biomédicale intitulée : « Evaluation de l'efficacité d'un programme d'activité physique individualisé, associé à une alimentation équilibrée enrichie en fruits et légumes, sur l'évolution de la masse grasse chez des femmes en surpoids ou obèses, dans le cadre de la prévention du diabète de type 2 à la Réunion. Etude LIPOXmax-Réunion.» dont le CHR de La Réunion est le promoteur.

Avant de prendre une décision, il est important que vous lisiez attentivement ces pages qui vous apporteront les informations nécessaires concernant les différents aspects de cette recherche. N'hésitez pas à poser toutes les questions que vous jugerez utiles à votre médecin.

Votre participation est entièrement volontaire. Si vous ne désirez pas prendre part à cette recherche, vous continuerez à bénéficier de la meilleure prise en charge médicale possible, conformément aux connaissances actuelles.

Pourquoi cette recherche?

Le diabète de type 2 est une maladie très répandue à la Réunion. Ce diabète apparaît le plus souvent chez des personnes en surpoids ou obèses, chez qui l'excès de masse grasse peut avoir différents effets biologiques : une baisse de la sensibilité à l'insuline entraînant une utilisation insuffisante du sucre par l'organisme ; un état d'inflammation chronique ; une baisse des défenses contre certains effets de l'environnement (en particulier l'oxydation). C'est pourquoi il est recommandé à ces personnes de perdre de la masse grasse corporelle pour combattre ces effets et prévenir l'apparition du diabète et de ses complications, en consommant quotidiennement des fruits et légumes et en pratiquant une activité physique modérée régulière. Cependant, des questions se posent sur la meilleure façon de pratiquer l'activité physique et sur son efficacité lorsqu'elle n'est pas prescrite selon les caractéristiques de chaque individu. Nous vous proposons donc de participer à un protocole de recherche visant à étudier les effets de différents types d'activités physiques associés à une alimentation riche en fruits et légumes.

Quel est l'objectif de cette recherche?

L'objectif de cette étude est d'évaluer l'efficacité de trois programmes d'activités physiques associés à une alimentation riche en fruits et légumes, sur la masse grasse chez femmes en surpoids ou obèses.

Comment se déroule cette recherche?

L'étude dure 2 ans au total avec une période d'inclusion de 2 mois et 5 mois de suivi. Il est prévu d'inclure au total 126 patientes dans cette recherche. Un tirage au sort (appelé randomisation) permettra de constituer 3 groupes de 42 patientes chacun. La réalisation de ce tirage au sort implique que ni vous ni les responsables de l'étude ne choisissent le groupe dans lequel vous êtes incluse et désigne celles qui auront :

- 4 séances hebdomadaires de vélo dans une salle de l'hôpital, sous le contrôle d'un éducateur sportif, pendant 5 mois. Chaque séance est réalisée pendant 1 heure à une intensité légère, individualisée en fonction du métabolisme à l'effort **(groupe 1).**
- le même programme avec des séances de vélo qui vont durer environ 40 minutes à une intensité supérieure standardisée **(groupe 2).**
- le conseil de pratiquer 30 minutes par jour d'activité physique de leur choix (marche, vélo, natation…) à une intensité dite « modérée », sur leur lieu de vie, un parcours sportif etc. **(groupe 3).** Les personnes de ce groupe ont un suivi téléphonique mensuel et tiennent un « cahier d'activité physique » où ils devront reporter la nature de l'activité (marche, vélo …), la fréquence des séances, la durée de chaque séance et l'intensité de l'effort en inscrivant leur niveau d'essoufflement suivant l'échelle de Borg et la fréquence cardiaque d'entraînement. Ce cahier devra être rapporté à l'éducateur sportif tous les 15 jours, lors de leur passage pour venir chercher les fruits et légumes, afin d'archiver les caractéristiques de chaque séance; il leur est proposé de porter un podomètre lors des séances de marche.

Qui peut participer ?

Ce projet d'intervention s'adresse exclusivement aux **patientes** :

- âgées de 20 à 40 ans
- ayant un Indice de Masse Corporelle compris entre 27 et 40 kg/m²
- non diabétique (glycémie < 1,26 g/L et HbA1c < 6,5%)
- considérées comme cliniquement stables
- pouvant participer à un programme de réentrainement à l'effort
- sédentaires (moins de 2 heures d'activité physique par semaine)
- patientes ayant signé un consentement de participation à l'étude
- sans activité professionnelle
- affiliées à un régime de Sécurité Sociale

Qui ne peut pas participer ? Patientes :

- Présentant une hypertension artérielle sévère (Pression Artérielle Systolique ≥180 mmHg et/ou Pression artérielle Diastolique ≥110 mmHg)
- Présentant une hypertension artérielle (≥140/90 mmHg) non contrôlée par un traitement médical ou dont le traitement médical comprend des bétabloquants ou des inhibiteurs calciques
- Ayant une contre-indication absolue et relative à l'épreuve d'effort (ACC/AHA, 2002) et/ou à l'entraînement physique
- Dans l'incapacité de réaliser l'épreuve d'effort maximale et/ou l'épreuve d'effort métabolique
- Ayant une pathologie cardiovasculaire et/ou respiratoire non équilibrée révélée par l'épreuve d'effort
- Portant un stimulateur ou un défibrillateur cardiaque
- Ayant des troubles du rythme ventriculaire sévères non stabilisés sous traitement
- Ayant présenté des évènements cardio-vasculaires récents (décompensation cardiaque ou traitement par drogues inotropes positives ou angioplastie, datant de moins de 10 jours, chirurgie cardiaque datant de moins de 3 mois, maladie valvulaire nécessitant une correction chirurgicale, myopéricardite en évolution, troubles du rythme ventriculaire sévères non stabilisés sous traitement)
- Ayant une myopathie connue et documentée

- Ayant une affection cancéreuse
- Ayant une maladie aiguë et chronique inflammatoire
- Ayant une insuffisance rénale chronique
- Opérées du tube digestif
- Sous corticoïdes, hormones thyroïdiennes, antidépresseurs ou neuroleptiques
- Ayant une pathologie associée évolutive entraînant une altération importante de l'état général
- Ayant une grossesse en cours ou un désir de grossesse avant la visite de fin d'étude.
- Ayant un déficit mental empêchant la compréhension du consentement éclairé et des protocoles
- Participant à un autre protocole de recherche
- Ayant participé dans le mois précédent à un programme de réentraînement à l'effort ou suivi d'un régime amaigrissant.

Que vous demandera-t-on ?

1-Vous avez été informée de la mise en place de cette recherche par le Dr. Favier, médecin du service d'endocrinologie – maladie métabolique – diabétologie du GHSR.
Si vous remplissez les premiers critères d'inclusion, un rendez-vous est pris avec le médecin investigateur qui vous explique cette recherche et vous propose d'y participer. Vous lui posez toutes les questions que vous jugez utile et prenez le temps de réfléchir à votre participation.

Si vous êtes d'accord pour participer à cette étude, après signature du consentement de participation, lors de la visite de pré-inclusion pour laquelle vous devrez être **à jeun**, seront réalisés les examens permettant de vérifier si vous pouvez être inclus : examen clinique et anthropométrique (poids, taille, tour de taille et tour de hanches), mesure de la glycémie capillaire et de Hb1Ac, dosage urinaire du taux de βHCG, épreuve d'effort cardiorespiratoire maximale, réalisée par un cardiologue (consiste à pédaler sur un vélo pendant 15 minutes).

Si les critères d'éligibilité sont vérifiés, vous serez alors inclus et pourrez alors commencer:

2- Visites J_0, J_1 et J_2 (dans le service d'endocrinologie – maladie métabolique – diabétologie du GHSR)

A J0 :
-Une prise de sang **à jeun** (depuis 11h) permettant de prélever 13 ml est réalisée. Divers paramètres sanguins tels que la glycémie veineuse, l'insulinémie, le cholestérol total, le HDL/LDL-Cholestérol, les triglycérides, la CRP, l'albumine et la créatinine seront mesurés (3 ml sont nécessaires pour ces analyses). La quantité de sang restante (2 tubes de 5 ml chacun) sera conservée afin de pouvoir mesurer le statut oxydatif et inflammatoire.
-Les urines sont également recueillies (10 ml) pour l'analyse de la consommation effective de fruits et légumes (protéinurie, albuminurie, acides caféique, chlorogénique, férulique et gallique).
-Vous avez une mesure de masse grasse corporelle par un appareil de radiologie à très faible émission, appelé absorptiomètre biphotonique et par une balance impédancemètre. Ces deux mesures sont rapides (10 minutes) indolores et sans danger.
-Vous répondez à un questionnaire sur votre activité physique habituelle, votre consommation alimentaire, vos données socio - démographiques, votre qualité de vie.

Le lendemain, vous reviendrez au service d'endocrinologie – maladie métabolique – diabétologie du GHSR pour pratiquer une autre épreuve d'effort sur vélo, moins intense, et un peu plus longue (30 minutes environ) afin d'évaluer votre métabolisme à l'effort.

Enfin, le jour suivant, vous participez à un atelier nutritionnel organisé par une diététicienne, qui vous informe sur la composition et les modes de préparation d'une alimentation équilibrée.

3- Visites J_3 à M_5.

A partir de ce moment là, vous aurez, une activité physique régulière pendant 5 mois, selon le programme qui correspond au groupe (1, 2 ou 3) dans lequel vous avez été randomisée.

Par ailleurs, quel que soit le groupe auquel vous participez, vous recevrez gratuitement une ration de 5 fruits et légumes par jour pendant les 5 mois de la recherche. Deux fois par semaine vous viendrez récupérer votre « panier » de fruits et légumes stocké par la cuisine du GHSR. Vous vous engagez par la présente à consommer personnellement tous les fruits et légumes qui vous seront donnés. Cependant, afin de vérifier que vous consommez bien ces fruits et légumes, des dosages seront effectués lors des prises de sang et des prélèvements d'urines réguliers seront pratiqués lors de votre passage pour venir chercher vos rations de fruits et légumes, sans vous en avertir à l'avance, afin de doser les composés urinaires liés à la consommation de fruits et légumes.

Deux fois par mois un auto questionnaire (avec aide de l'enquêteur si vous avez des difficultés de lecture) vous est proposé, portant sur votre ressenti sur ces activités physiques et ce mode d'alimentation supplémenté en fruits et légumes.

4- Visites M_3 et M_5 (au service d'endocrinologie – maladie métabolique – diabétologie du GHSR)
- un examen clinique et anthropométrique (poids, taille, tour de taille et tour de hanches) est effectué
- une nouvelle prise de sang **à jeun** (depuis 11h) permettant de prélever à nouveau 13 ml est réalisée pour effectuer les mêmes dosages qu'à J_0.
- les urines sont également recueillies (10 ml) pour l'analyse de la consommation effective de fruits et légumes (protéinurie, albuminurie, acides caféique, chlorogénique, férulique et gallique) et le dosage urinaire du taux de βHCG.
- vous pratiquez une épreuve d'effort sur vélo, de 30 minutes, afin d'évaluer votre métabolisme à l'effort.
- vous répondez à un questionnaire sur votre qualité de vie.
- vous avez une mesure de masse grasse corporelle par un appareil de radiologie à très faible émission, appelé absorptiomètre biphotonique (à la visite M_5 uniquement) et par une balance impédancemètre.

Tous les échantillons sanguins prélevés au cours de l'étude, seront détruits, après dosage, à la fin de l'étude.

Pour résumer, on vous demande :

- De pratiquer les tests et analyses qui vous sont proposés et de répondre aux questionnaires.

  Les tests, les analyses et les questionnaires seront réalisés, au service d'endocrinologie – maladie métabolique – diabétologie du GHSR, sur 3 matinées **à jeun**. 13 ml de sang et 10 ml d'urines seront nécessaires. Des analyses d'urines supplémentaires (en plus de celles prévues par le protocole à J_0, M_3 et M_5) seront effectuées de manière inopinée pour contrôler la consommation de fruits et légumes.
- De respecter le programme d'activité physique qui vous est attribué. En cas de problème médical ou de fatigue importante la séance peut être allégée voire supprimée. Si une personne ne se montre pas suffisamment assidue, soit 4 absences consécutives ou 9 absences au total, il lui sera demandé d'arrêter sa participation à la recherche.
- De venir chercher vos rations de fruits et légumes 2 fois par semaine et de les consommer à raison de 5 par jours.

Afin d'estimer au mieux les effets de l'activité physique associée à une supplémentation en fruits et légumes, nous vous demanderons de ne pas changer vos habitudes, en particulier votre activité physique quotidienne.

Indemnisation : toutes les participantes reçoivent une indemnité de 100 euros par mois pendant 5 mois pour les contraintes liées à leur participation (déplacements, garde d'enfants etc.). Cette indemnité vous est versée mensuellement, en totalité ou partiellement en fonction de votre participation, totale ou partielle, aux séances d'activités physiques. Ces 100 euros par mois correspondent à environ 20 séances d'activité physique par mois (4 par semaine), soit 5 euros par séance qui seront déduits de l'indemnisation mensuelle, pour chaque absence.

Quels sont les bénéfices attendus?

Quel que soit votre groupe (1, 2 ou 3) d'activité physique, les bénéfices attendus sont une diminution de la masse grasse, une meilleure tolérance à l'effort, l'apprentissage de connaissances et compétences relatives à la gestion de son activité physique (utilisation des cardiofréquencemètres, des niveaux d'essoufflements, des règles de "bonnes pratiques"…), une amélioration des données biologiques et de la qualité de vie.
Au bout des 5 mois, les résultats sur l'efficacité des différents programmes d'activité physique seront présentés à l'ensemble des participantes. S'ils mettent en évidence l'efficacité supérieure d'un programme par rapport aux autres, les personnes des 2 autres groupes bénéficieront pendant un mois d'un encadrement pour un apprentissage du contrôle de l'activité physique à l'aide des cardiofréquencemètres.

Quels sont vos droits ?

Dans le cadre de la recherche biomédicale à laquelle le CHR de La Réunion vous propose de participer, un traitement informatique de vos données personnelles va être mis en œuvre pour permettre d'analyser les résultats de la recherche au regard de l'objectif de cette dernière qui vous a été présenté. A cette fin, les données médicales vous concernant seront transmises au promoteur de la recherche ou aux personnes ou sociétés agissant pour son compte, en France ou à l'étranger. Ces données seront identifiées par un code. Ces données pourront également, dans des conditions assurant leur confidentialité, être transmises aux autorités de santé françaises ou étrangères et à d'autres entités du CHR de La Réunion.

Conformément aux dispositions de la loi relative à l'informatique, aux fichiers et aux libertés, vous disposez à tout moment d'un droit d'accès et de rectification des données informatisées vous concernant (loi n° 2004-801 du 6 août 2004 modifiant la loi n° 78-17 du 6 janvier 1978 relative à l'informatique, aux fichiers et aux libertés). Vous disposez également d'un droit d'opposition à la transmission des données couvertes par le secret professionnel susceptibles d'être utilisées dans le cadre de cette recherche et d'être traitées. Vous pouvez également accéder directement ou par l'intermédiaire du médecin de votre choix à l'ensemble de vos données médicales en application des dispositions de l'article L1111-7 du code de la santé publique. Ces droits s'exercent auprès du médecin qui vous suit dans le cadre de la recherche et qui connaît votre identité.

Conformément à la loi n°2004-806 du 9 août 2004 relative à la Politique de Santé Publique :
- cette recherche a obtenu un avis favorable du Comité de Protection des Personnes Sud-Ouest et Outre Mer III le 26.10.2011 et l'autorisation de l'Agence Française Sécurité Sanitaire des Produits de Santé (Afssaps) le 15.06.2011 (art L1121-4 du code de la santé publique),
- le promoteur de cette recherche, CHR de La Réunion (Site du Groupe Hospitalier Sud Réunion, avenue François Mitterrand BP350 – 97 448 Saint Pierre Cedex), a souscrit une assurance de responsabilité civile auprès de la SHAM (n° 110424) (art L1121-10 du code de la santé publique),
- les personnes ayant subi un préjudice après participation à une recherche biomédicale peuvent faire valoir leurs droits auprès des commissions régionales de conciliation et d'indemnisation des accidents médicaux (art L1121-10 et L1142-3 du code de la santé publique),
- lorsque cette recherche sera terminée, vous serez tenus informés personnellement des résultats globaux par votre médecin dès que ceux-ci seront disponibles, si vous le souhaitez (art L1122-1 du code de la santé publique).

Après avoir lu cette note d'information, n'hésitez pas à poser à votre médecin toutes les questions que vous désirez. Après un délai de réflexion, si vous acceptez de participer à cette recherche, vous devez compléter et signer le formulaire de consentement de participation. Un exemplaire du document complet vous sera remis.

D'avance nous vous remercions pour votre participation à cette étude.

Docteur FAVIER
Investigateur principal

« Evaluation de l'efficacité d'un programme d'activité physique individualisé, associé à une alimentation équilibrée enrichie en fruits et légumes, sur l'évolution de la masse grasse chez des femmes en surpoids ou obèses, dans le cadre de la prévention du diabète de type 2 à la Réunion»
Etude : LIPOXmax-Réunion

Promoteur de la recherche : **CHR de La Réunion**
Investigateur principal : ***Docteur François FAVIER***
Amendement n°1, version n°2.1 du 10/10/2011

Je soussignée...*(nom, prénom)* certifie avoir lu et compris la note d'information qui m'a été remise.

J'ai eu la possibilité de poser toutes les questions que je souhaitais au Dr François FAVIER qui m'a expliqué la nature, les objectifs, les risques potentiels et les contraintes liées à ma participation à cette recherche.

Avant de participer à cette recherche, j'ai bénéficié de tests sanguins et d'examens médicaux dont les résultats m'ont été communiqués et me permettent de participer.

Je connais la possibilité qui m'est réservée d'interrompre ma participation à cette recherche à tout moment sans avoir à justifier ma décision et je ferai mon possible pour en informer le médecin qui me suit dans la recherche. Cela ne remettra naturellement pas en cause la qualité des soins ultérieurs.

J'ai eu l'assurance que les décisions qui s'imposent pour ma santé seront prises à tout moment, conformément à l'état actuel des connaissances médicales.

J'ai bien noté que cette recherche est menée conformément aux Articles L1121-1 et suivants du Code de la Santé Publique, relatifs à la protection des personnes qui se prêtent à des recherches biomédicales et conformément aux Bonnes Pratiques Cliniques.

J'ai pris connaissance que cette recherche a reçu l'avis favorable du Comité de Protection des Personnes Sud-Ouest et Outremer III le 31.08.2011 (amendement N°1 le 26.10.2011) et l'autorisation de l'Afssaps le 15.06.2011 et a fait l'objet d'une déclaration à la Commission Nationale Informatique et Libertés (CNIL).

Le promoteur de la recherche (CHR de La Réunion, Site du Groupe Hospitalier Sud Réunion, avenue François Mitterrand BP350 – 97 448 Saint Pierre Cedex) a souscrit une assurance de responsabilité civile en cas de préjudice auprès de la SHAM (*n° 110424*).

J'accepte que seules les personnes qui collaborent à cette recherche ou qui sont mandatées par le promoteur, ainsi qu'éventuellement le représentant des Autorités de Santé, aient accès à l'information dans le respect le plus strict de la confidentialité.

J'ai bien noté que, conformément aux dispositions de la loi relative à l'informatique, aux fichiers et aux libertés, je dispose d'un droit d'accès et de rectification. Je dispose également d'un droit d'opposition à la transmission des données couvertes par le secret professionnel susceptibles d'être utilisées dans le cadre de cette recherche et d'être traitées. Ces droits s'exercent auprès du médecin qui me suit dans le cadre de cette recherche et qui connaît mon identité.

Mon consentement ne décharge en rien l'investigateur et le promoteur de la recherche de leurs responsabilités à mon égard. Je conserve tous les droits garantis par la loi.
Les résultats globaux de la recherche me seront communiqués directement, si je le souhaite, conformément à la loi du 4 mars 2002 relative aux droits des malades et à la qualité du système de santé.

Ayant disposé d'un temps de réflexion suffisant avant de prendre ma décision, j'accepte librement et volontairement de participer à la recherche LIPOXmax-Réunion : ☐ oui ☐ non
Je pourrai à tout moment demander des informations complémentaires au médecin qui m'a proposé de participer à cette recherche, n° téléphone : François Favier : 0262 71 98 29

Fait à……………………….le : |__|__|__|__|__|__|__|__|__|	Fait à……………………….le : |__|__|__|__|__|__|__|__|__|
Signature de la patiente :	Signature du médecin :

- 1er feuillet (original) : à conserver à part par l'investigateur pendant 30 ans dans un lieu sûr fermant à clé
- 2ème feuillet : à remettre au patient/sujet après signatures
- 3ème feuillet : à ranger dans le classeur investigateur, il sera demandé ultérieurement par le promoteur

ANNEXE 3 : INFORMATION DANS LA PRESSE

Le Centre Hospitalier Régional, site du Groupe Hospitalier Sud Réunion, RECHERCHE

Des patientes âgées de 20 à 40 ans, résidant à Saint Pierre, pour participer pendant 5 mois à une recherche biomédicale intitulée : « Evaluation de l'efficacité d'un programme d'activité physique individualisé associé à une supplémentation équilibrée enrichie en fruits et légumes sur l'évolution de la masse grasse chez des femmes en surpoids ou obèses à l'île de la Réunion. Etude : LIPOXmax-Réunion »

Une indemnité de frais de déplacements sera versée aux participantes
