# Supplementary material for: Individualized Exercise Training at Maximal Fat Oxidation Combined with Fruit and Vegetable-Rich Diet in Overweight or Obese Women: The LIPOXmax-Réunion Randomized Controlled Trial
Source: PLoS One. 2015 Nov 10;10(11):e0139246. doi: 10.1371/journal.pone.0139246 (PMC4640859; doi:10.1371/journal.pone.0139246)
Supplement: S2 Protocol — (PDF) [file pone.0139246.s003.pdf]

Individualized exercise training at maximal fat oxidation combined with fruit and vegetable-rich diet in overweight or obese women: The LIPOX-max Réunion randomized controlled trial.

This research has been registered in http://www.clinicaltrials.gov/ under the n° NCT01464073.

Background: On La Réunion Island, in year 2000, the REDIA study revealed a type-2 diabetes prevalence among the 30-69 years old people above 17%, a prevalence of obesity among women of 20% and 10% for men. According to the literature dedicated to preventive strategies and/or treatment of metabolic diseases, regular physical activity associated with a balanced diet and an intake of fruits and vegetables, can reduce body fat mass and prevent the complications related to obesity. These complications are partly related to the inflammatory and oxidative stress that accompanies obesity. A diet rich in fruits and vegetables, may improve the inflammatory and oxidative status. However there is no consensus on the intensity of regular physical activity in the practices of prevention and care. These parameters are generally not individualized. International recommendations cover a wide range of exercise intensity (between 40% and 60% of maximal oxygen uptake). The research group of Jacques Mercier (CERAMM Lapeyronie-CHU Montpellier) showed the benefit of the training conducted at a relatively low intensity of effort, corresponding to the maximum rate of lipid oxidation (LIPOXmax), and its effects on the fat mass, the insulin sensitivity and the muscle metabolism, in overweight or obese people.

Main objective: to study the effects of three programs of rehabilitation training associated with a diet supplemented with fruits and vegetables, on reducing body fat in overweight or obese people.

Secondary objectives: to study the effects on the oxidative stress and the inflammatory status, compare the efficacy of these 3 programs of physical training on body composition modifications, insulin sensitivity, lipid utilization during exercise and quality of life, describe feasibility in population (compliance, motivation).

Abstract: According to data from the literature, a balanced diet combined with moderate exercise prevents biological complications related to obesity and reduce the incidence of type-2 diabetes by promoting fat loss. However, there is no consensus on the intensity of physical activity prescribed. Of note, the benefits of individualized training sessions performed at low-intensity eliciting the maximum rate of lipid oxidation measured by indirect calorimeter (LIPOXmax) on fat loss, sensitivity to insulin and muscle metabolism in obese or diabetic patients. However, an individualized LIPOXmax exercise training has not yet been compared to a standardized training conventionally used in the treatment of obese subjects. Furthermore, no study has yet evaluated the impact of LIPOXmax training on inflammatory and antioxidant status among overweight and obese patients. We propose to compare among overweight or obese patients, the effects of an individualized training at an intensity corresponding to LIPOXmax, the effects of a standardized training to 60% of VO_{2max}, and the effects of a moderate and regular physical activity prescribed according to guidelines of Good Medical Practices.

At M0 participants will have anthropometric and functional examinations and laboratory tests, and will follow nutritional education sessions. They will be then randomized into three arms: arm 1 LIPOXmax physical activity, arm 2 60% VO_{2max} physical activity, arm 3 physical activity according to guidelines of Good Medical Practices. Participants of the arms 1 and 2 will practice under the control of a sports instructor; participants of the arm 3 will be regularly monitored by telephone. The procedure will last 5 months. At M3 and M5 anthropometric and biological examinations will be replicated and participants will answer to questionnaires on quality of life and experience of physical activity during and after the intervention.

Primary outcome: body fat mass reduction (M_5-M_0), in kilograms measured by Dual Energy X-Ray Absorptiometry.

Secondary outcomes: improvement of biological parameters (blood glucose, HbA1c, insulin, cholesterol, HDL / LDL, triglycerides); improvement in body composition (weight, BMI, waist circumference, hip circumference, muscle mass), and in lipid utilization during exercise; improvement of the quality of life; demonstration of the feasibility in population (adherence, compliance).

Study design: controlled, randomized, of superiority, not blind trial in 126 people divided into three arms of 42 people per arm: arm 1 LIPOXmax intensity of effort, arm 2 60% of VO_{2max} intensity of effort, arm 3 intensity of effort according to the Guidelines of Good Medical Practices.
Inclusion criteria: women, 20-40 years old, 27 ≤ BMI ≤40 kg / m², sedentary, considered clinically stable, able to rehabilitation training, having signed an informed consent, without professional activity.
Exclusion criteria: blood glucose ≥ 1.26 g / l, HbA1c ≥ 6.5%, severe hypertension (SBP ≥ 180 mmHg and / or DBP ≥ 110 mmHg), hypertension (≥140/90 mmHg) untreated or treated by beta blocker or calcium blocker, absolute and relative contraindication to the maximal exercise test (ACC / AHA 2002) and / or physical training, inability to achieve the maximum exercise test and / or the metabolic exercise test by indirect calorimeter, uncompensated cardiovascular and / or respiratory disease revealed by exercise test, pacemaker or defibrillator, recent cardiovascular events (heart failure, treated by positive inotropic drugs, angioplasty within the last 10 days, cardiac surgery within the last 3 months, valvular disease requiring surgical correction, evolving myopericarditis, severe ventricular arrhythmias non stabilized under treatment), known and documented myopathy, cancer, acute and chronic inflammatory disease, end stage renal disease, digestive system operation, treated by corticoids, thyroid hormone, antidepressant or neuroleptics, pregnancy, mental deficiency that prevents the understanding of informed consent and protocol, participation to another research protocol, attendance in the previous month to a program of rehabilitation training or a diet, associated evolutionary disease causing significant impairment of general condition.
Intervention: the 126 subjects will undergo the following exams: weight, height, waist and hip circumference, blood pressure, body fat measurement by dual energy X-ray absorptiometry (M_0 and M_5) and by impedancemetry, maximal cardiopulmonary exercise and effort metabolic test (by indirect calorimeter to determine the LIPOXmax and the crossing points of the substrates), laboratory tests (blood glucose, HbA1c, insulin, cholesterol total, HDL, LDL, triglycerides, CRP, albumin, creatinine, urinary parameters, antioxidant status, inflammatory markers); questionnaires about medical history and sociodemographic data; food survey (frequency questionnaire, quantization by photos), questionnaires on physical activity (Baecke amended), quality of life (SF-36), experience of physical activity. All the participants will have an initial nutritional education session and will receive a free supplement of 5 fruits and vegetables per day during all the intervention, and random urine assays will be performed to monitor the consumption of fruits and vegetables. Participants will be randomized into three arms. Arm 1: LIPOXmax arm. Subjects will perform a physical activity 4x1 hour per week at the optimal intensity for fat oxidation (LIPOXmax) measured by indirect calorimeter. Arm 2: physical activity standard arm (60% of VO_{2max} measured by cardio respiratory exercise test). Subjects will realize 4 sessions per week and duration of sessions will be adjusted so that arms 1 and 2 have the same total energy expenditure by session. Arm 3: control arm. The subjects initially receive the recommendations of good medical practice for physical activity (achieving at least 3h30min per week of "moderate" exercise). The intervention will last 5 months in total. For arms 1 and 2: physical activity on ergo meter bicycle controlled by a sports trainer, with the goal of gradual empowerment. For arm 3: self-directed activities with regular telephone follow-up, holding of a book on physical activities and use of a pedometer during walking sessions. Indemnization: all the participants will receive a monthly allowance for the constraints due to their participation.
Number of subjects: to highlight a difference in average change of 1.5 kg fat mass, with a standard deviation of 2.0 kg, a first species risk of 1.67% (5% / 3), a power of 80%, it is necessary to include 38 subjects. In such circumstances it will be possible to demonstrate a theoretical minimum significant difference between arms of: - 2.3% on changes at 5 month of average percentage of body fat (SD = 3%) and - 3.8 kg on changes at 5 months of average weight (SD = 5 kg). Due to missing data (dropouts, lost) rate estimated at 10%, it is planned to include 42 subjects per arm, or 126 in total.
Statistical analysis: single analysis in intention to treat (arms assigned by the randomization). Overall first species risk = 5%. Bilateral formulation of statistical tests. Statistical methods used: bivariate analysis (one factor ANOVA, Pearson Chi2 test or Fisher test), multivariate analysis (ANCOVA, logistic regression). Taking into account the longitudinal data (M0-M3-M5) with generalized estimating equations (GEE). Graphic representation of the evolutions over the study.
