# Supplementary material for: Individualized Exercise Training at Maximal Fat Oxidation Combined with Fruit and Vegetable-Rich Diet in Overweight or Obese Women: The LIPOXmax-Réunion Randomized Controlled Trial
Source: PLoS One. 2015 Nov 10;10(11):e0139246. doi: 10.1371/journal.pone.0139246 (PMC4640859; doi:10.1371/journal.pone.0139246)
Supplement: S1 Table — (DOC) [file pone.0139246.s004.doc]

**Table S1: Analysis of bias of selection for baseline inclusion characteristics among participants and non-participants at M5**

|  | | **All randomized participants** | | **Randomized participants without**  **participation at M5** | | **Randomized participants with**  **participation at M5** | | **p value** |
| --- | --- | --- | --- | --- | --- | --- | --- | --- |
|  | | n = 136 | | n = 27 | | n = 109 | |  |
| **Socio-demographic characteristics** | |  |  |  |  |  |  |  |
| Age (years) | | 30.1 | ± 5.6 | 29.2 | ± 4.4 | 30.3 | ± 5.8 | 0.335* |
| School level : | |  |  |  |  |  |  |  |
| Secondary school (pupils from 10 to 15) | | 8.8% | | 7.4% | | 9.2% | | 0.475* |
| Upper forms (pupils from 15 to 18) | | 46.3% | | 37.0% | | 48.6% | |  |
| A-level or Higher school | | 44.9% | | 55.6% | | 42.2% | |  |
| Recipient of CMU | | 64.0% | | 63.0% | | 64.2% | | 0.903 |
| **Anthropometric characteristics** | |  |  |  |  |  |  |  |
| BMI (kg/m²) | | 33.1 | ± 3.5 | 33.2 | ± 3.1 | 33.1 | ± 3.6 | 0.915* |
|  | < 30 kg/m² | 24.3% | | 14.8% | | 26.6% | | 0.315* |
|  | ≥ 30 kg/m² | 75.7% | | 85.2% | | 73.4% | |  |
| Waist size (cm) | | 97.9 | ± 9.0 | 99.0 | ± 10.0 | 97.7 | ± 8.7 | 0.491 |
|  | < 88 cm | 12.5% | | 7.4% | | 13.8% | | 0.524* |
|  | ≥ 88 cm | 87.5% | | 92.6% | | 86.2% | |  |
| Fat mass (kg) | | 39.9 | ± 7.9 | 40.0 | ± 7.2 | 39.8 | ± 8.1 | 0.934 |
| Fat mass (%) | | 47.1 | ± 3.8 | 46.5 | ± 3.6 | 47.2 | ± 3.9 | 0.377 |
| Fat Free Mass (kg) | | 44.2 | ± 4.8 | 45.6 | ± 5.5 | 43.9 | ± 4.6 | 0.091 |
| Fat Free Mass (%) | | 51.3 | ± 3.6 | 52.0 | ± 3.4 | 51.2 | ± 3.7 | 0.328 |
| **Blood pressure** | |  |  |  |  |  |  |  |
| Systolic blood pressure (mm Hg) | | 117.0 | ± 9.9 | 116.8 | ± 9.1 | 117.0 | ± 10.1 | 0.914 |
| Diastolic blood pressure (mm Hg) | | 74.4 | ± 8.5 | 73.9 | ± 7.4 | 74.6 | ± 8.7 | 0.729 |
| **Lipid profile** | |  |  |  |  |  |  |  |
| Total Cholesterol (mmol/L) | | 4.5 | ± 0.8 | 4.6 | ± 0.8 | 4.5 | ± 0.8 | 0.900 |
| LDL Cholesterol (mmol/L) | | 2.8 | ± 0.7 | 2.8 | ± 0.7 | 2.8 | ± 0.7 | 0.748 |
| HDL Cholesterol (mmol/L) | | 1.2 | ± 0.3 | 1.3 | ± 0.4 | 1.2 | ± 0.3 | 0.698* |
| HDL-C/LDL-C ratio | | 0.48 | ± 0.21 | 0.51 | ± 0.29 | 0.48 | ± 0.18 | 0.816* |
| Triglycerides (mmol/L) | | 1.1 | ± 0.5 | 1.1 | ± 0.6 | 1.1 | ± 0.5 | 0.490* |
| **Glucose profile** | |  |  |  |  |  |  |  |
| HbA1c (%) | | 5.5 | ± 0.3 | 5.4 | ± 0.3 | 5.5 | ± 0.3 | 0.373* |
| Fasting plasma glucose (mmol/L) | | 5.0 | ± 0.5 | 4.9 | ± 0.6 | 5.0 | ± 0.4 | 0.844* |
| Insulin (m UI/L) | | 20.0 | ± 11.3 | 18.7 | ± 11.3 | 20.3 | ± 11.3 | 0.422* |
| HOMA-IR index | | 4.5 | ± 2.8 | 4.2 | ± 2.6 | 4.5 | ± 2.8 | 0.438* |
| Insulin resistant (HOMA-IR index > 2.5) | | 80.0% | | 77.8% | | 80.6% | | 0.747 |

^† ‡^ Data are medians and interquartile range. Otherwise, data are means ± SD or percentages. CMU: universal health coverage.

HOMA-IR index: Homeostasis Model Assessment estimated insulin resistance index.

*p value* refers to comparison between participants and non-participants at M5 by Student’s test or Wilcoxon rank-sum test (*) for quantitative variables, and by Chi2 test or Fisher exact test (*) for qualitative variables.
