# Supplementary material for: Individualized Exercise Training at Maximal Fat Oxidation Combined with Fruit and Vegetable-Rich Diet in Overweight or Obese Women: The LIPOXmax-Réunion Randomized Controlled Trial
Source: PLoS One. 2015 Nov 10;10(11):e0139246. doi: 10.1371/journal.pone.0139246 (PMC4640859; doi:10.1371/journal.pone.0139246)
Supplement: S2 Table — (DOC) [file pone.0139246.s005.doc]

**Table S2: Analysis of bias of selection for exercise tests at inclusion among participants and non-participants at M5**

|  | **All randomized participants** | | **Randomized participants without**  **participation at M5** | | **Randomized participants with**  **participation at M5** | | **p**  **value** |
| --- | --- | --- | --- | --- | --- | --- | --- |
|  | n = 136 | | n = 27 | | n = 109 | |  |
| **Maximal exercise test** |  |  |  |  |  |  |  |
| VO2max (L/min) | 1.9 | ± 0.3 | 1.9 | ± 0.4 | 1.9 | ± 0.3 | 0.571* |
| VO2max (mL/min/kg FFM ) | 42.5 | ± 6.3 | 41.4 | ± 7.6 | 42.8 | ± 6.0 | 0.126* |
| HRmax (bpm) | 173.0 | ± 11.3 | 170.8 | ± 12.5 | 173.6 | ± 11.0 | 0.273 |
| RER | 1.1 | ± 0.1 | 1.1 | ± 0.1 | 1.1 | ± 0.1 | 0.605* |
| Wmax (W) | 136.0 | ± 19.9 | 136.3 | ± 21.6 | 136.0 | ± 19.5 | 0.923* |
| **Metabolic exercise test** |  |  |  |  |  |  |  |
| HR at LIPOXmax (bpm) | 120.9 | ± 13.6 | 120.1 | ± 14.1 | 121.1 | ± 13.6 | 0.749 |
| W at LIPOXmax (W) | 43.4 | ± 12.1 | 43.5 | ± 9.9 | 43.4 | ± 12.7 | 0.970* |
| W at LIPOXmax (% Wmax) | 32.2 | ± 8.3 | 33.0 | ± 8.9 | 31.9 | ± 8.1 | 0.736* |
| Borg at LIPOXmax (6-20) | 11.2 | ± 1.1 | 11.3 | ± 1.2 | 11.2 | ± 1.1 | 0.612* |
| VO2 at LIPOXmax (ml/min) | 843.5 | ± 142.3 | 850.1 | ± 121.7 | 841.9 | ± 147.5 | 0.790 |
| VO2 at LIPOXmax (% VO2max) | 45.7 | ± 8.3 | 46.5 | ± 9.0 | 45.5 | ± 8.2 | 0.588 |
| **Maximal Lipid Oxidation** |  |  |  |  |  |  |  |
| MFO (mg/min) | 153.4 | ± 42.9 | 162.4 | ± 37.5 | 151.1 | ± 44.0 | 0.117* |
| MFO (mg/min/kg FFM) | 3.5 | ± 1.0 | 3.6 | ± 1.0 | 3.5 | ± 1.0 | 0.456* |

Data are means ± SD. FFM: Fat Free Mass. HR: Heart Rate. RER: Respiratory Exchange Ratio. W: Power. MFO: Maximal Fat Oxidation.

*P value* refers to comparison between participants and non-participants at M5 by Student’s test or Wilcoxon rank-sum test (*).
